# Supplementary material for: Respiratory supercomplexes act as a platform for complex III‐mediated maturation of human mitochondrial complexes I and IV
Source: EMBO J. 2020 Jan 8;39(3):e102817. doi: 10.15252/embj.2019102817 (PMC6996572; doi:10.15252/embj.2019102817)
Supplement: Supplementary file 1 — Expanded View Figures PDF [file EMBJ-39-e102817-s001.pdf]

Expanded View Figures

Figure EV1. Complexome profiling of cIII2-containing structures in samples from in WT and Δ4-CYB cells solubilized with DDM (related to Figs 2–4).

A Heatmaps of cIII2 structural subunits and assembly factors derived from the DDM-solubilized samples in the experiment where Δ4-CYB cells were labeled with the heavy (H) amino acids. Black = 0; yellow = 0.5; red = 1 relative peptide intensities of the most frequent peptide found in each of the samples individually.

B Complexome profiles of the cIII2 structural subunits found in both cell lines in the two reciprocal labeling experiments. The graphs plot the relative peptide peak intensities along the lane, setting the maximum to 1.0 versus the molecular mass, calculated using the individual complexes as the standards to generate a calibration curve. The relative amounts of the proteins between the two cell lines were determined by calculating the H/L ratios of peptides that were present in both WT (blue traces) and Δ4-CYB samples (red traces). The represented values are the mean ± SEM of the two reciprocal labeling experiments.

C Heatmaps of cIII2 structural subunits and assembly factors derived from the digitonin-solubilized samples in the experiment where WT cells were labeled with the heavy (H) amino acids. Black = 0; yellow = 0.5; red = 1 relative peptide intensities of the most frequent peptide found in each of the samples individually.

D Profiles of the subunits only found in the WT samples in the two reciprocal labeling experiments. The graphs plot the relative peptide peak intensities along the lane, setting the maximum to 1 versus the molecular mass, calculated using the individual complexes and supercomplexes as the standards to generate a calibration curve. The represented values are the mean ± SEM of the two reciprocal labeling experiments.

© 2019 The Authors

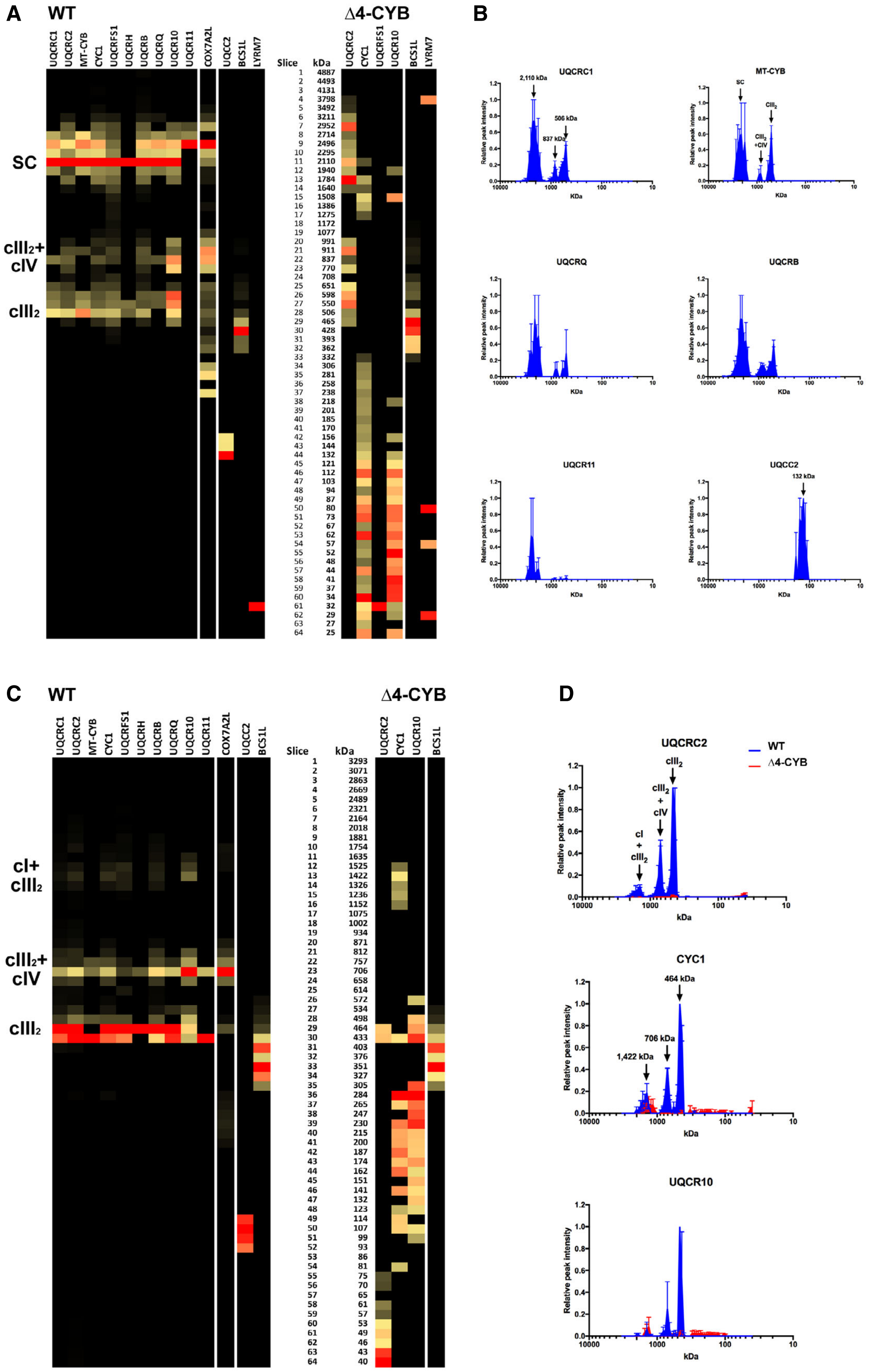
A
WT
Δ4-CYB
UQCRC1
UQCRC2
MT-CYB
CYC1
UQCRFS1
UQCRH
UQCRB
UQCRQ
UQCR10
UQCR11
COX7A2L
UQCC2
BCS1L
LYRM7
Slice
kDa
SC
cIII2+
cIV
cIII2
B
UQCRC1
2,110 kDa
837 kDa
506 kDa
MT-CYB
SC
CIII2
+CIV
CIII2
UQCRQ
UQCRB
UQCR11
UQCC2
132 kDa
Relative peak intensity
KDa
C
WT
Δ4-CYB
cI+
cIII2
cIII2+
cIV
cIII2
D
UQCRC2
WT
Δ4-CYB
cI
+
cIII2
cIII2
+
cIV
cIII2
CYC1
1,422 kDa
706 kDa
464 kDa
UQCR10
Relative peak intensity
kDa

Figure EV1.

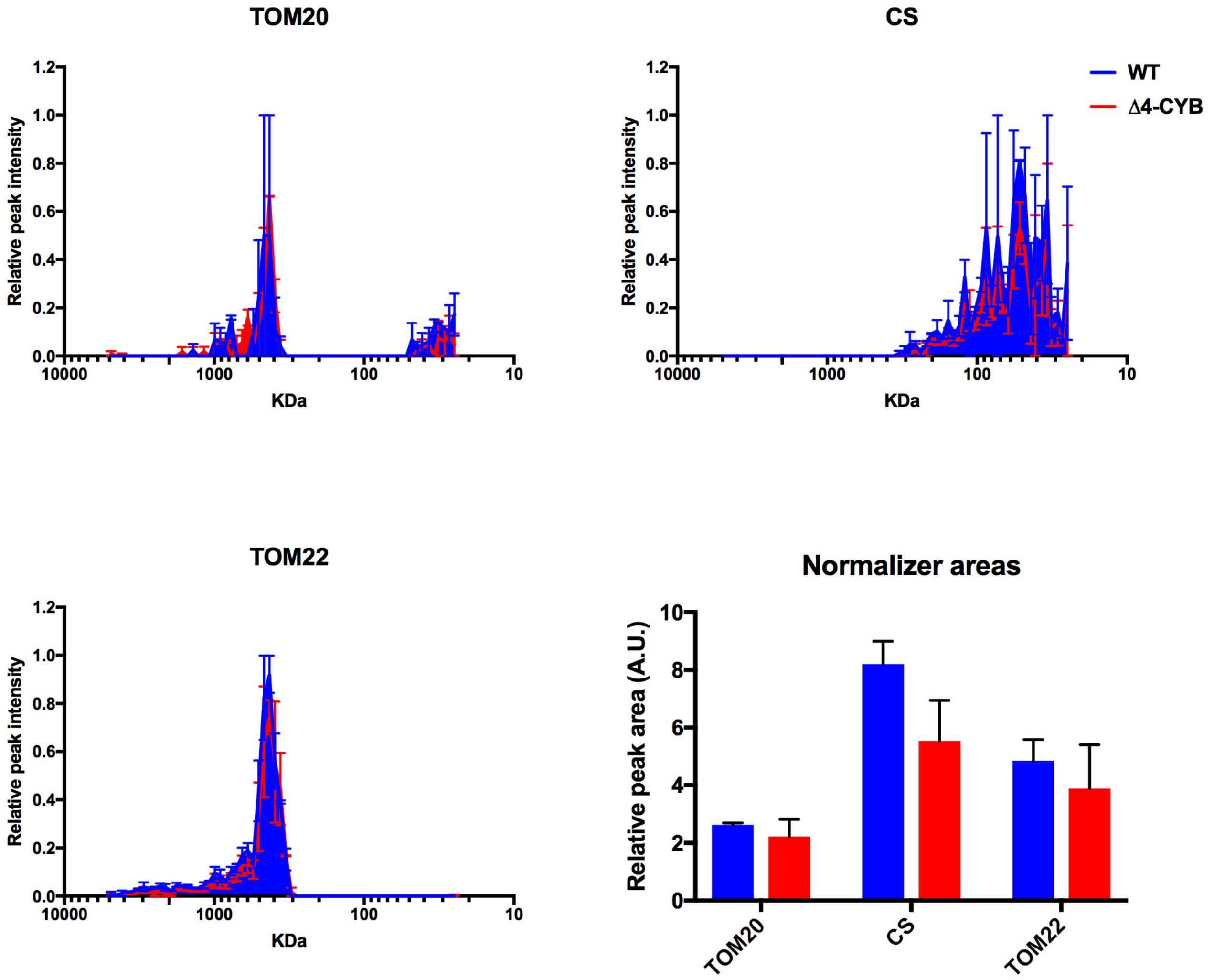

Figure EV2. Complexome profiling and area quantification of internal control proteins (related to Fig 3).
The complexome profiles of the three chosen proteins, citrate synthase (CS), and two members of the translocase of the outer membrane family (TOM20 and TOM22) were generated as in the main Fig 3. The graphs plot the relative peptide peak intensities along the lane, setting the maximum to 1 versus the molecular mass, calculated using the individual complexes and supercomplexes as the standards to generate a calibration curve. The relative amounts of the proteins between the two cell lines were determined by calculating the H/L ratios of peptides that were present in both WT (blue traces) and Δ4-CYB samples (red traces). The represented values are the mean ± SEM of the two reciprocal labeling experiments. The bar graph represents the quantification of the total peak area under the curves (AUC) defined by the peptide intensity peaks for the indicated proteins. The *x*-axis values were the slice number (1–64), and the *y*-axis values were the relative peptide intensity. The plotted values are mean ± SD (n = 2). The differences were not significant according to 2-way ANOVA with Sidak's multiple comparisons test.

© 2019 The Authors

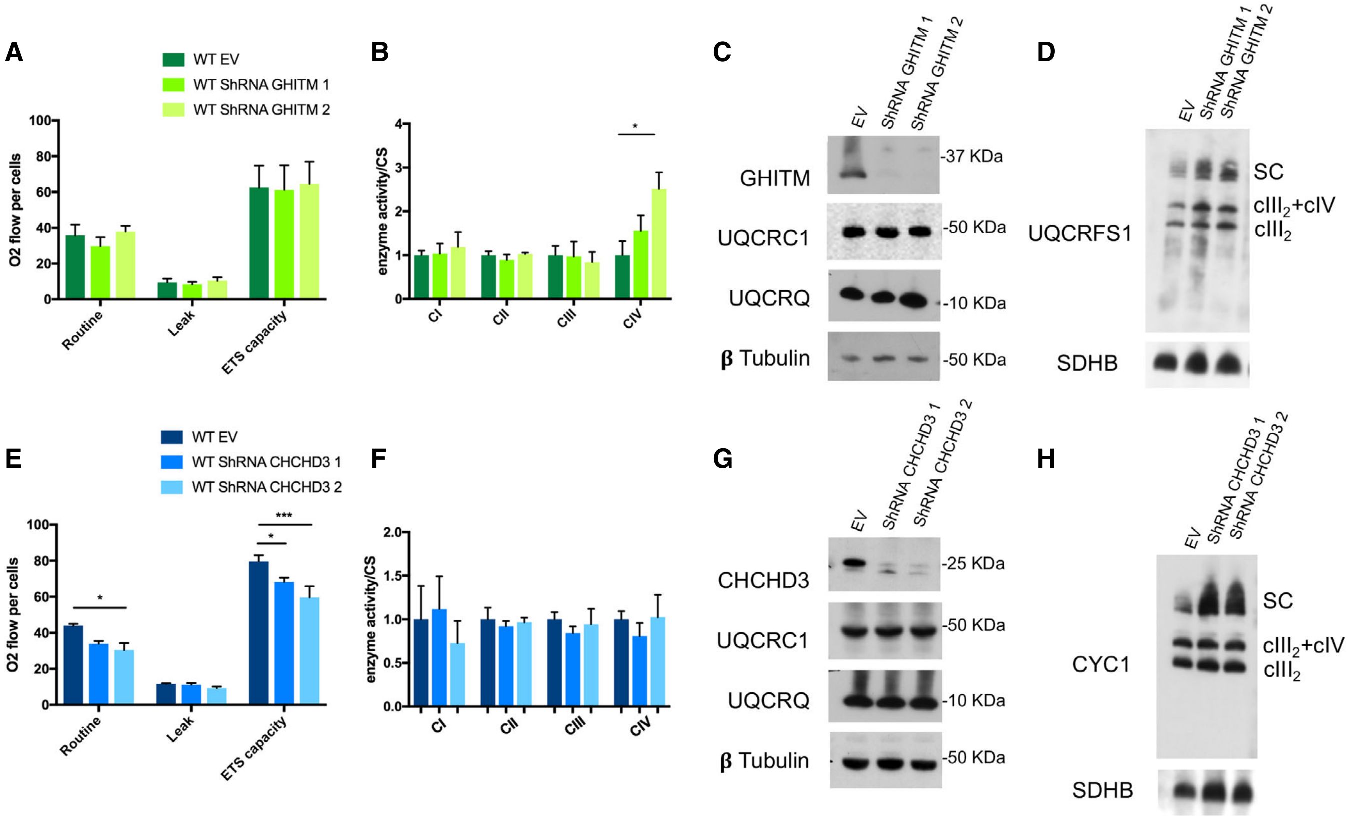

Figure EV3. Knock-down of either *GHITM* or *CHCHD3* does not produce cIII2 functional nor assembly defects (related to Fig 4).

A Oxygen consumption rates measured in WT cells transduced with lentiviral vectors encoding two different shRNAs specific for GHITM mRNA (shRNA GHITM 1 and shRNA GHITM 2) and with pLKO.1 without any shRNA insert (empty vector, EV). Respiration was measured in whole cells in the basal state (Routine), in the presence of oligomycin (Leak) and uncoupled with CCCP (ETS capacity), using a O2K high-resolution respirometer (Oroboros instruments). The plotted values are the mean ± SD (n = 4 for WT EV and WT shRNA GHITM 1 and n = 3 for WT shRNA GHITM 2).

B MRC enzyme activities normalized to the activity of citrate synthase (CS) measured in the same cell lines shown in (A). The plotted values are the mean ± SD (n = 2 biological replicates). Two-way ANOVA Tukey's multiple comparisons test *P = 0.0462 (CIV).

C SDS–PAGE, Western blot, and immunodetection analysis with the indicated specific antibodies.

D 1D BNGE, Western blot, and immunodetection analysis of digitonin-solubilized samples from the same three cell lines shown in (A).

E Oxygen consumption rates measured in 143B WT cells transduced with lentiviral vectors encoding two different shRNAs specific for CHCHD3 mRNA (shRNA CHCHD3 1 and shRNA CHCHD3 2) and with pLKO.1 without any shRNA insert (empty vector, EV). Respiration was measured in whole cells in the basal state (Routine), in the presence of oligomycin (Leak) and uncoupled with CCCP (ETS capacity), using a O2K high-resolution respirometer (Oroboros instruments). The plotted values are the mean ± SD (n = 4). Two-way ANOVA Tukey's multiple comparisons test *P = 0.0126 (Routine shRNA 2); *P = 0.0386 (ETS capacity shRNA 1); ***P = 0.0002 (ETS capacity shRNA 2).

F MRC enzyme activities normalized to the activity of citrate synthase (CS) measured in the same cell lines shown in (E). The plotted values are the mean ± SD (n = 4 biological replicates).

G SDS–PAGE, Western blot, and immunodetection analysis with the indicated specific antibodies.

H 1D BNGE, Western blot, and immunodetection analysis of digitonin-solubilized samples from the same three cell lines shown in (E).

Source data are available online for this figure.

© 2019 The Authors

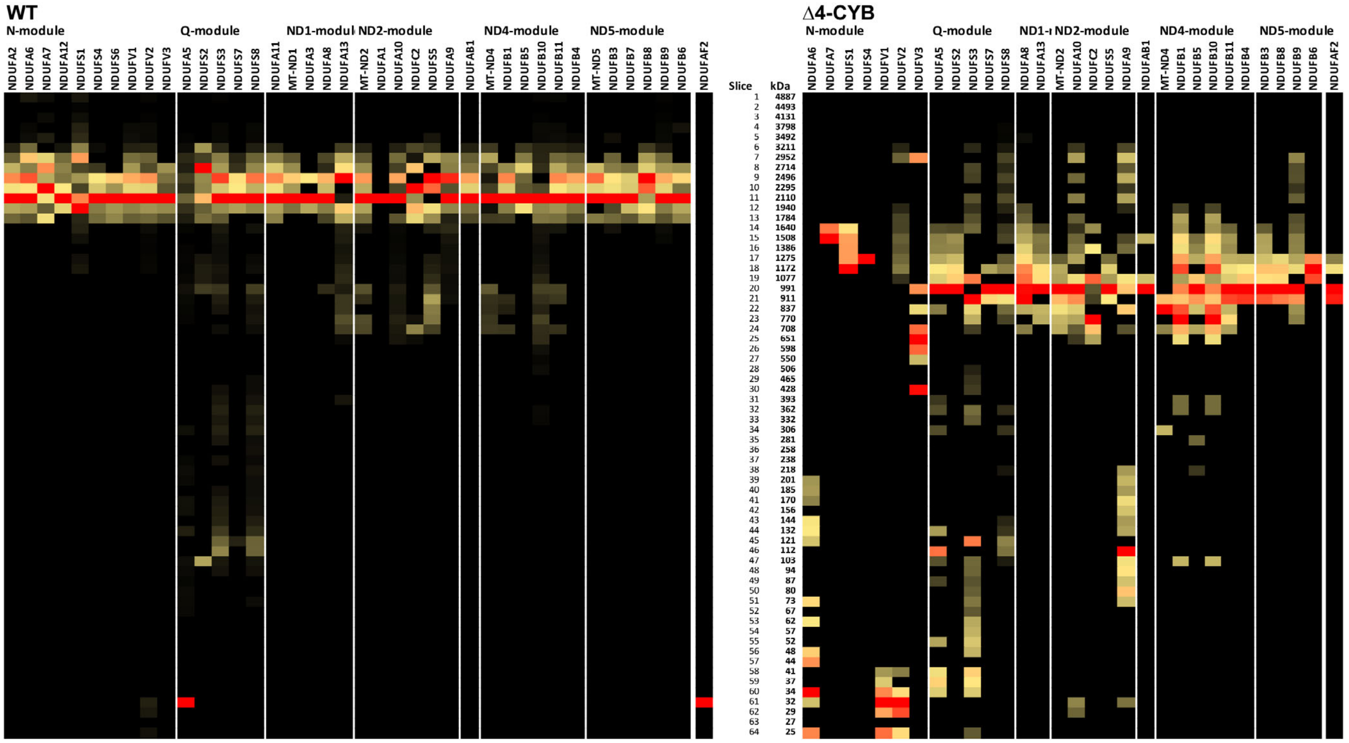

Figure EV4. Complexome profiling of cI-containing structures in WT and Δ4-CYB cells (related to Fig 5).

Heatmaps of the individual cI structural subunits and the NDUFAF2 assembly factor derived from the digitonin-solubilized samples in the experiment where WT cells were labeled with the heavy (H) amino acids. Black = 0; yellow = 0.5; red = 1 relative peptide intensities of the most frequent peptide found in each of the samples individually.

Figure EV5. CI assembly in WT and Δ4-CYB cells solubilized with DDM and in modulated NDUFAF2 levels (related to Fig 5).

A Complexome profiles of the cI structural modules found in both cell lines in the two reciprocal labeling experiments. The graphs plot the average of relative peptide peak intensities along the lane corresponding to the subunits of each module, setting the maximum to 1 versus the molecular mass, calculated using the individual complexes as the standards to generate a calibration curve. The relative amounts of the proteins between the two cell lines were determined by calculating the H/L ratios of peptides that were present in both WT (blue traces) and Δ4-CYB samples (red traces). The represented values are the mean ± SEM of the two reciprocal labeling experiments. The bar graph represents the quantification of the total peak area under the curves (AUC) defined by the peptide intensity peaks for the indicated cI modules. The *x*-axis values were the slice number (1-64), and the *y*-axis values were the relative peptide intensity. The plotted values are mean ± SD (n = 2). Two-way ANOVA with Sidak's multiple comparisons test $^{**}P$ = 0.0072 (Q-module); $^{**}P$ = 0.0029 (N-module); $^{*}P$ = 0.0102.

B A Myc-DDK (FLAG) tagged version of NDUFAF2 ($AF2^{Myc\text{-}DDK}$) was stably expressed in both WT and Δ4-CYB cybrids by transfection of the cloned cDNA in the pCMV6-Entry mammalian expression vector (Origene Cat#: RC207387). As negative controls, the two cell lines were also transfected with the empty pCMV6-Entry vector (EV), providing the resistance to neomycin but no expression of other relevant proteins.

C Complex I-IGA assay performed in the NDUAF2-overexpressing ($AF2^{Myc\text{-}DDK}$) and negative control (EV) cell lines after solubilizing the samples with digitonin and separating them on BNGE.

D Complex I enzymatic activity assay performed in the NDUAF2-overexpressing ($AF2^{Myc\text{-}DDK}$) and negative control (EV) cell lines. Results are expressed as mean ± SD (n = 3 biological replicates). No significant differences (ns) in cI activity were found between the NDUFAF2-overexpressing WT or Δ4-CYB cells and their corresponding negative control (one-way ANOVA with Tukey's multiple comparisons test).

E NDUFAF2 expression was knocked down both WT and Δ4-CYB cybrids by transfection of two different siRNAs targeting the NDUFAF2 transcript (AF2 siRNA1 and AF2 siRNA2, Sigma-Aldrich). Sigma's siRNA Universal Negative Control #1 was used in the experiments as well.

F Complex I-IGA assay performed in the cells transfected with both siRNAs and the negative control (CT(-) siRNA), after solubilizing the samples with digitonin and separating them on BNGE.

G Complex I enzymatic activity assay performed in the cells transfected with both siRNAs and the negative control cell lines. Results are expressed as mean ± SD (n = 3 biological replicates). No significant differences (ns) in cI activity were found between the silenced WT or Δ4-CYB cells and their corresponding negative control (one-way ANOVA with Tukey's multiple comparisons test).

Source data are available online for this figure.

 © 2019 The Authors

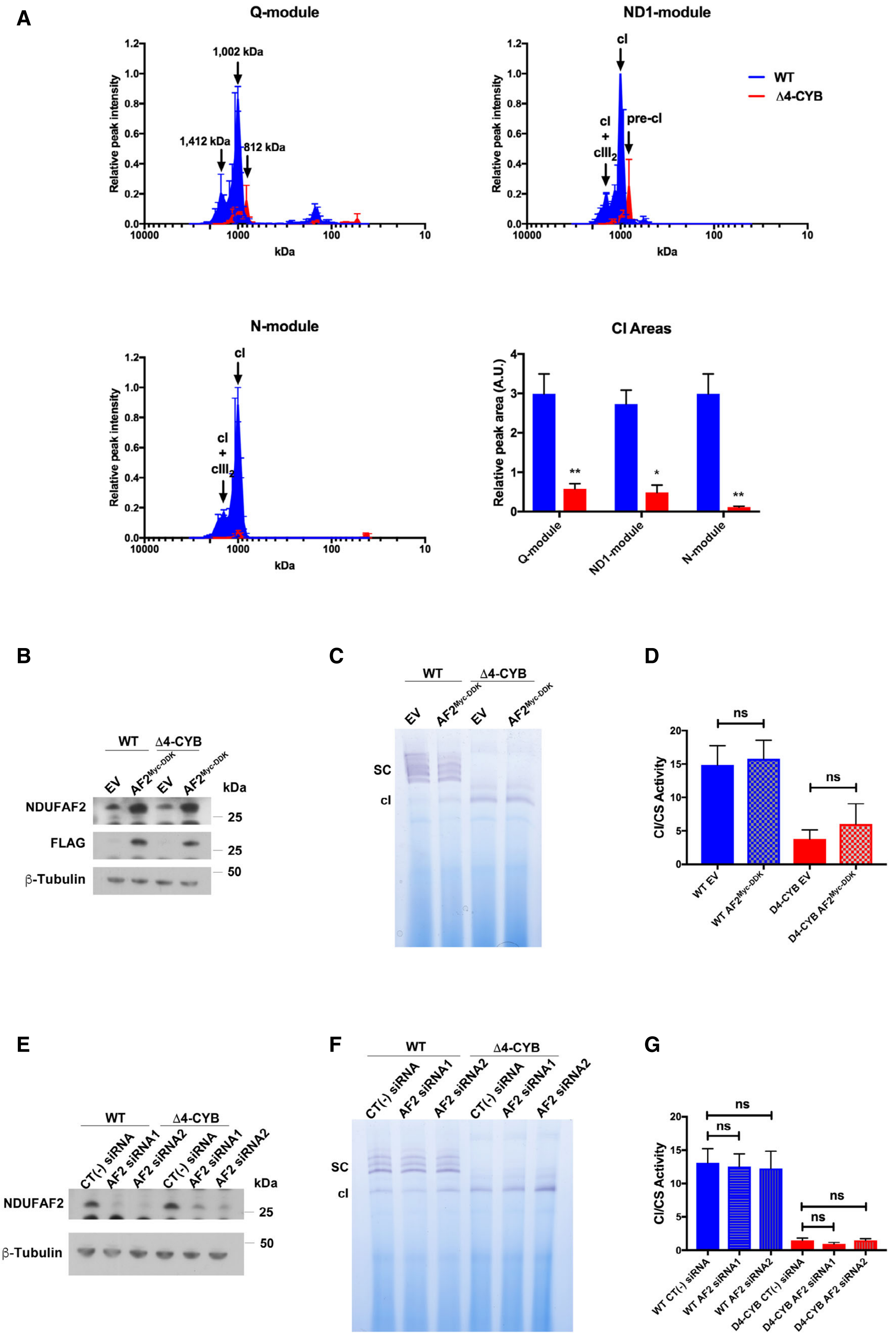
A
Q-module
1,002 kDa
1,412 kDa
812 kDa
Relative peak intensity
kDa
ND1-module
cI
cI + cIII2
pre-cI
WT
Δ4-CYB
N-module
CI Areas
Relative peak area (A.U.)
Q-module
ND1-module
N-module
B
WT
Δ4-CYB
EV
AF2Myc-DDK
kDa
NDUFAF2
FLAG
β-Tubulin
C
SC
cI
D
CI/CS Activity
ns
WT EV
WT AF2Myc-DDK
D4-CYB EV
D4-CYB AF2Myc-DDK
E
CT(-) siRNA
AF2 siRNA1
AF2 siRNA2
F
G
WT CT(-) siRNA
WT AF2 siRNA1
WT AF2 siRNA2
D4-CYB CT(-) siRNA
D4-CYB AF2 siRNA1
D4-CYB AF2 siRNA2

Figure EV5.
