# Supplementary material for: Respiratory supercomplexes act as a platform for complex III‐mediated maturation of human mitochondrial complexes I and IV
Source: EMBO J. 2020 Jan 8;39(3):e102817. doi: 10.15252/embj.2019102817 (PMC6996572; doi:10.15252/embj.2019102817)
Supplement: Supplementary file 2 — Source Data for Expanded View [file EMBJ-39-e102817-s009.zip › Figure_EV3_Source_Data.pdf]

Figure EV3C – Anti-GHITM and Anti-UQCRQ

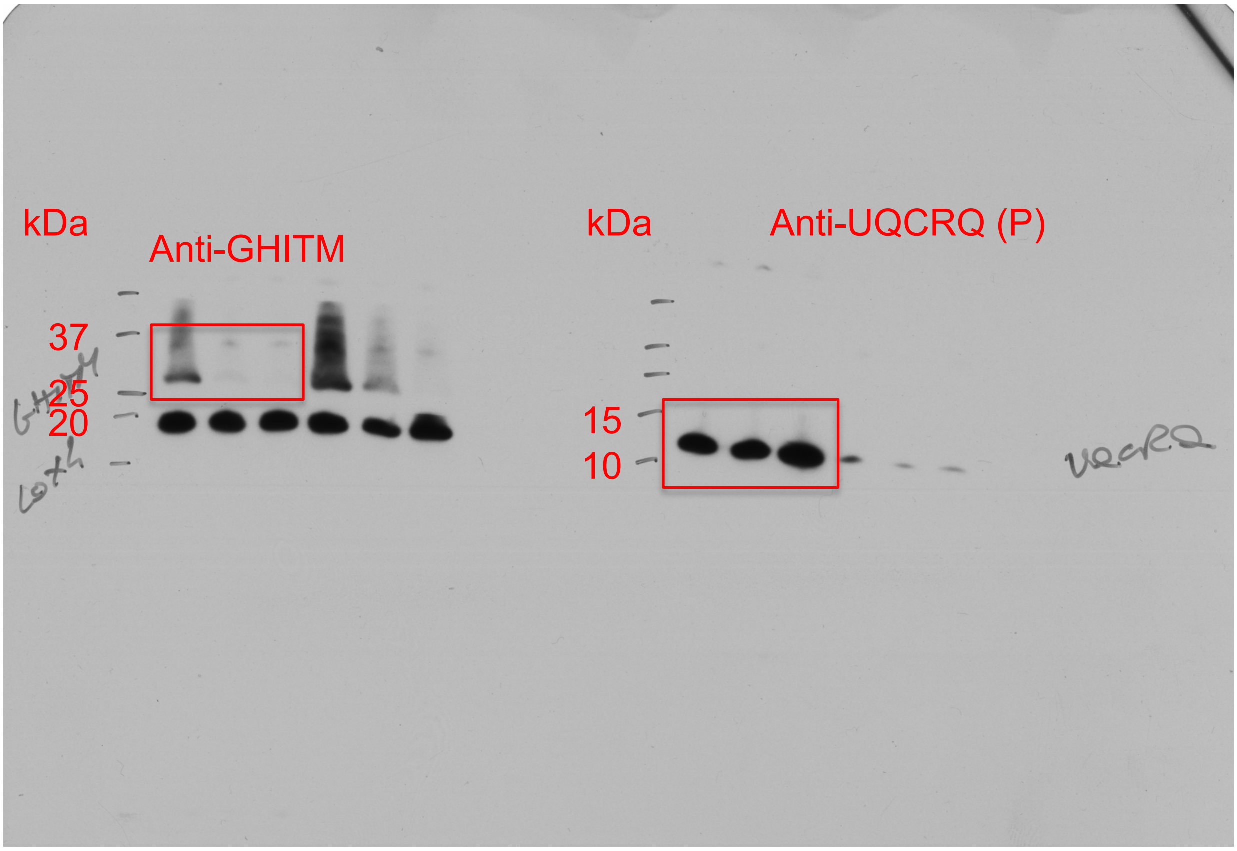

Figure EV3C – Anti-β-Tubulin

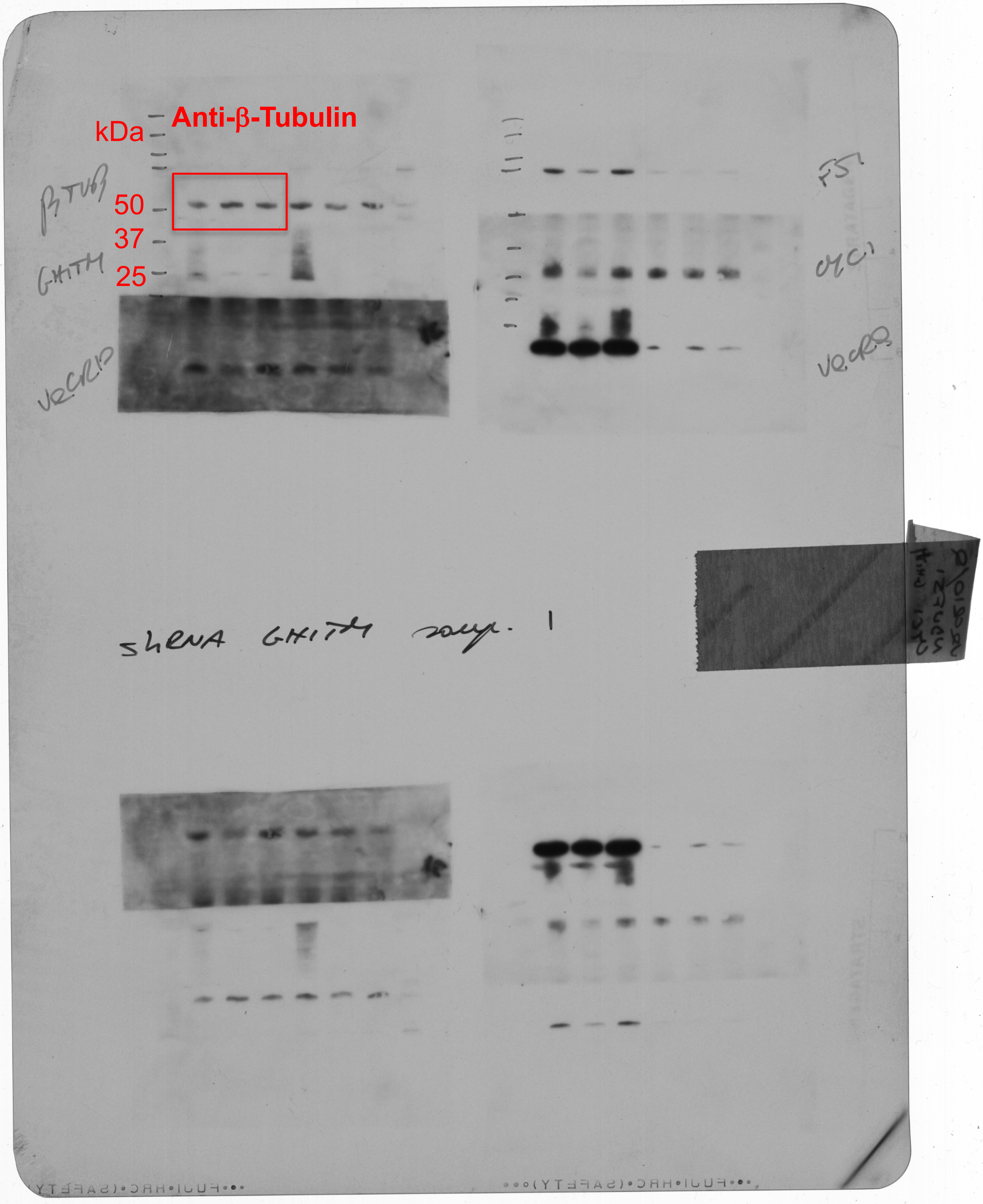

Figure EV3D

Anti-UQCRFS1

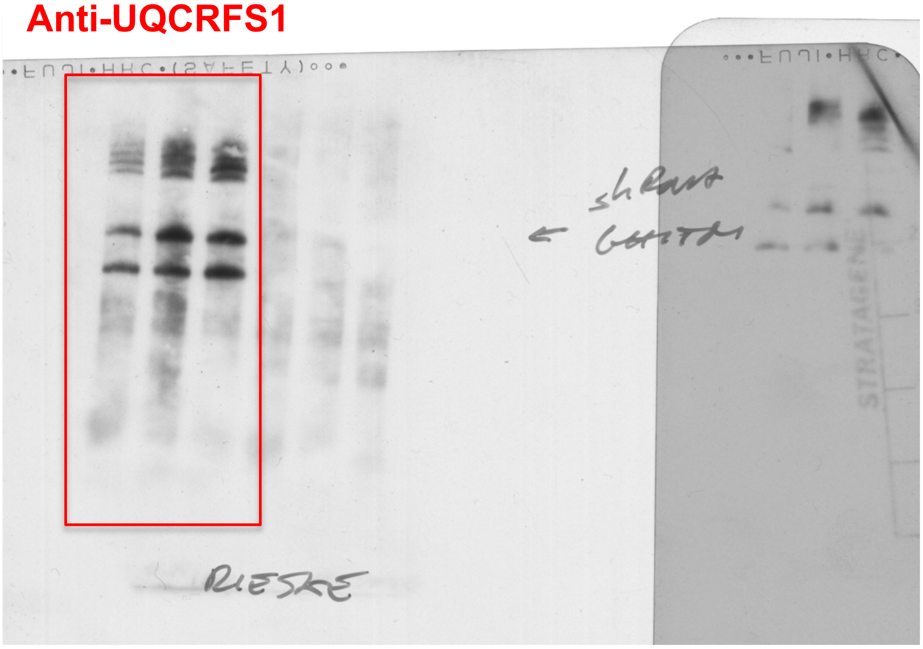

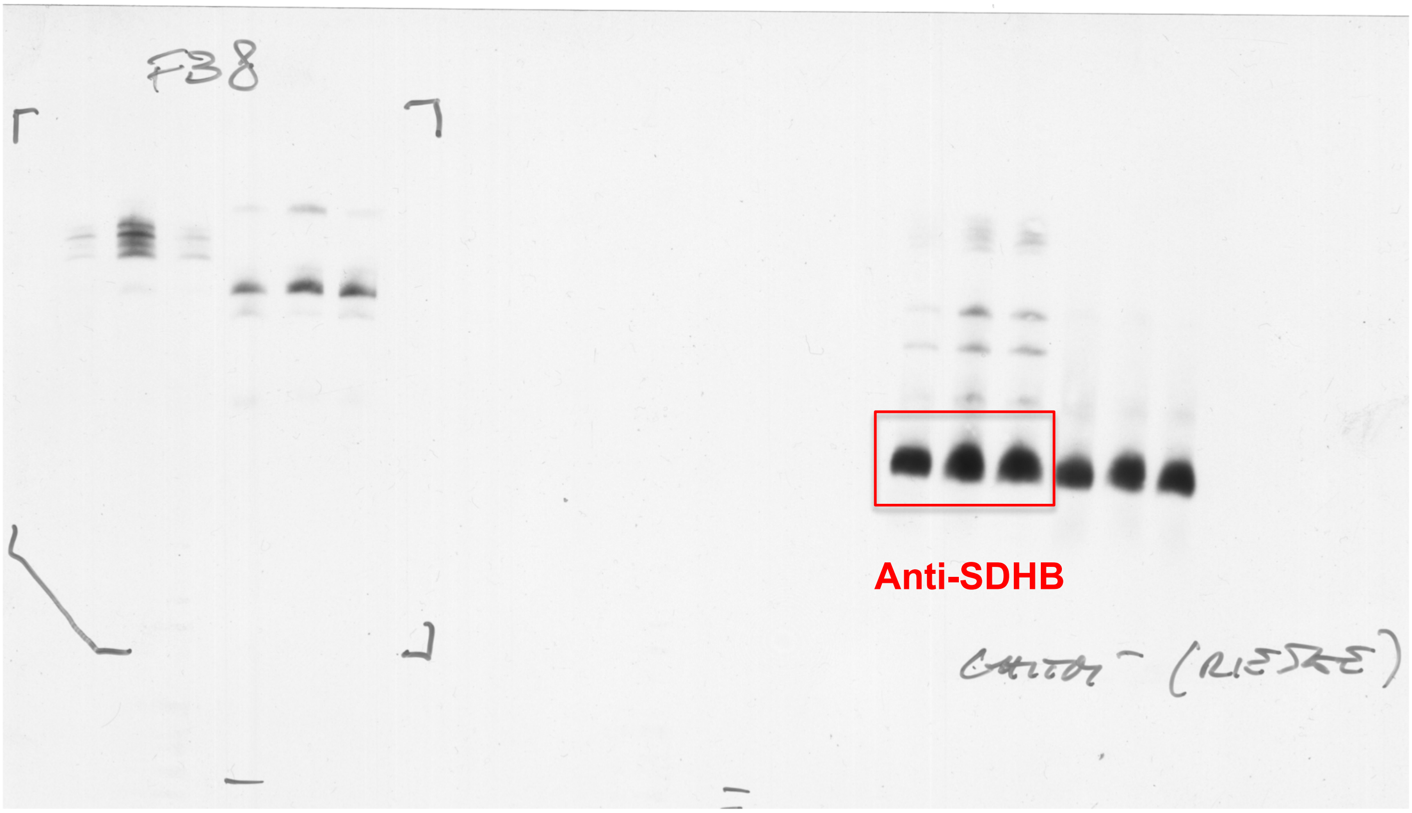

Figure EV3G – Anti-CHCHD3

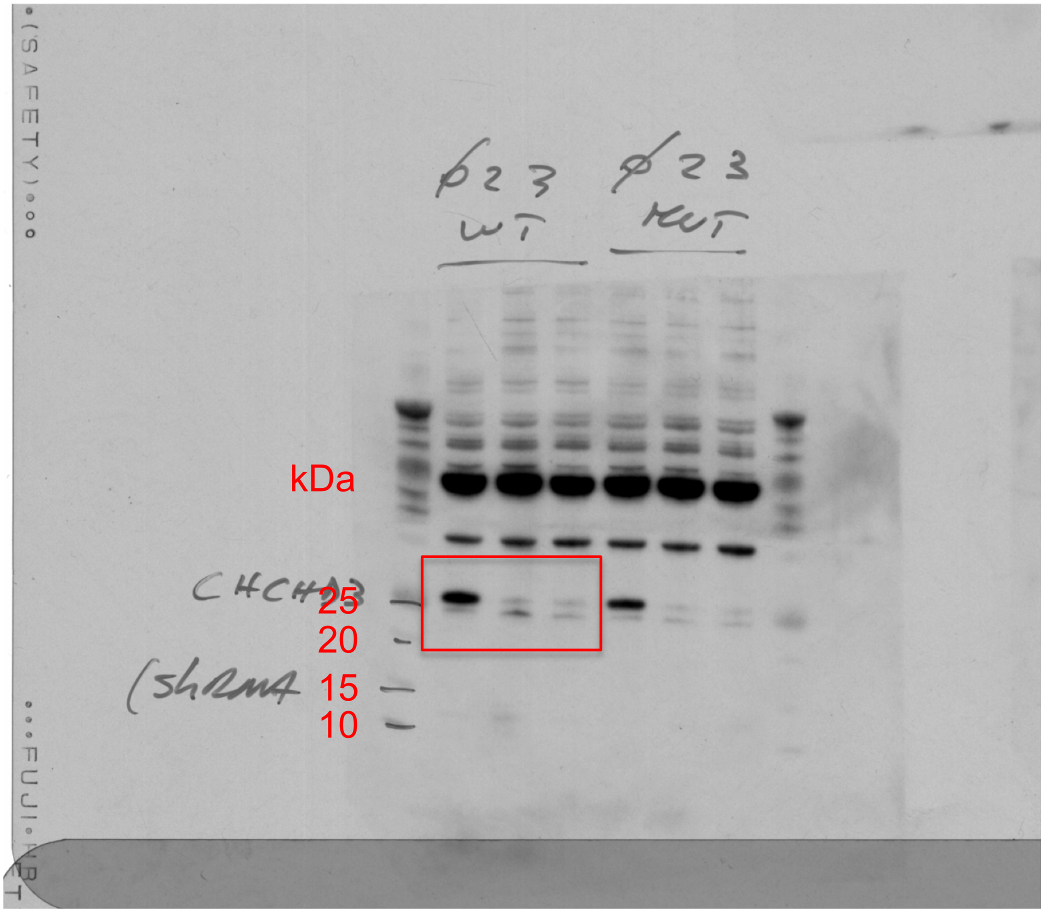

Figure EV3G – Anti-UQCRC1, Anti-UQCRQ & Anti-β-Tubulin

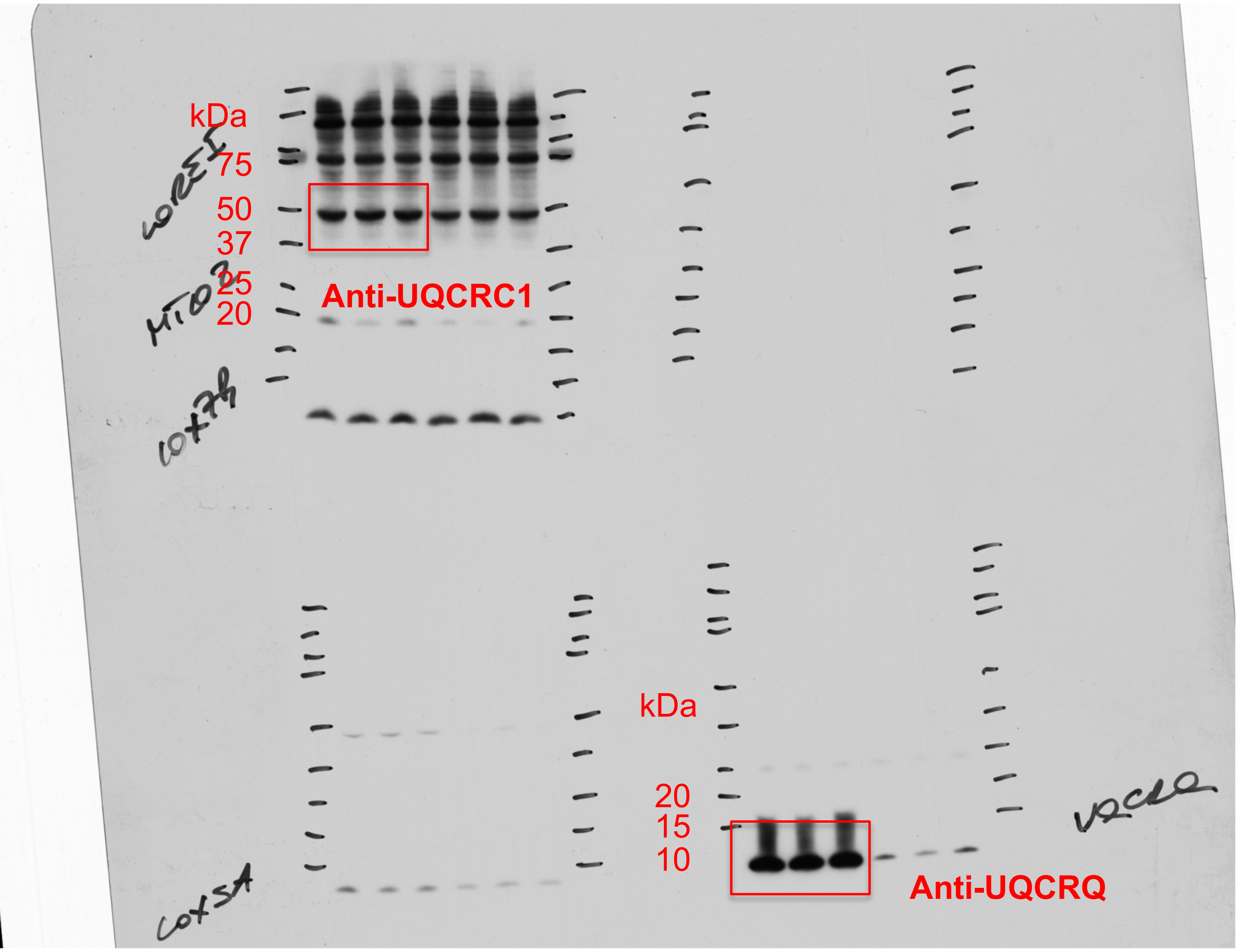

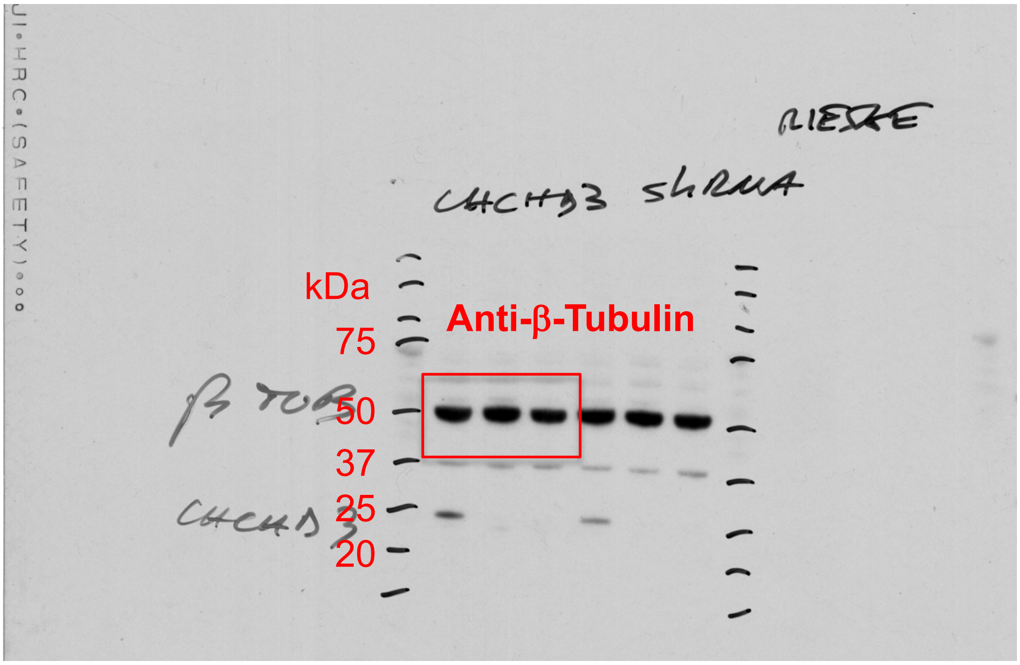

Figure EV3H

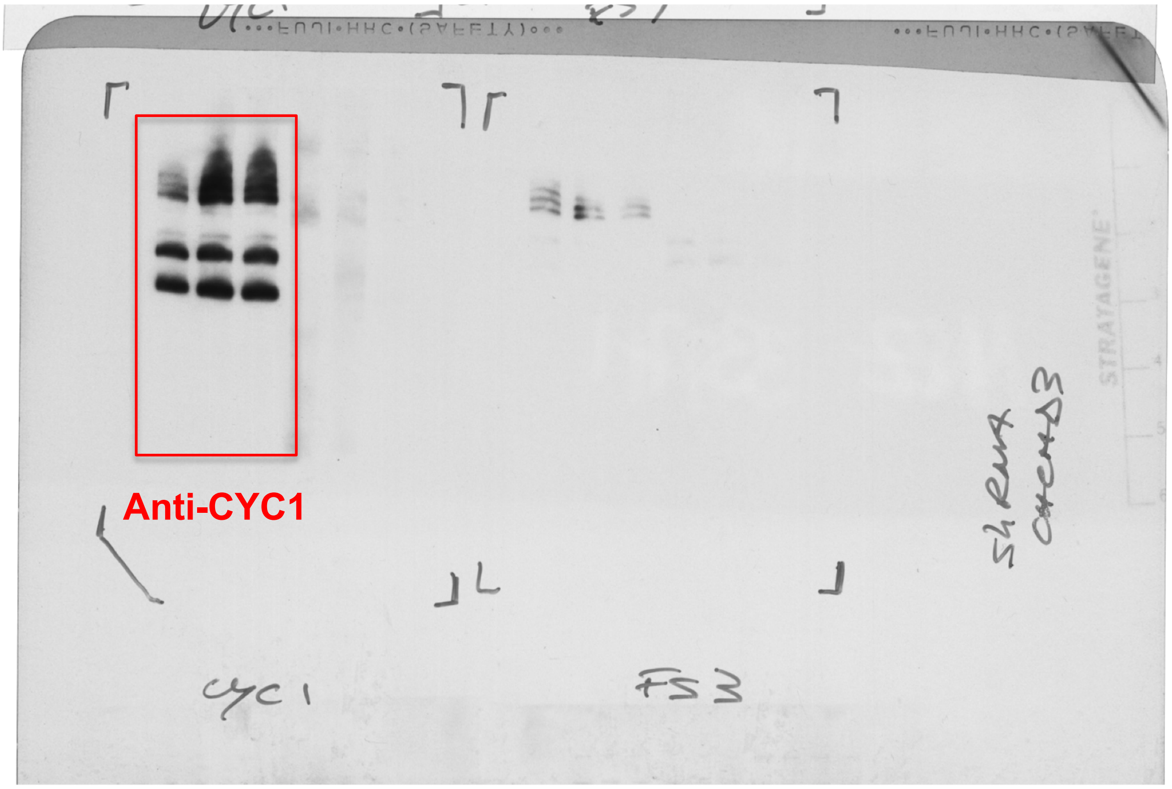
Anti-CYC1
CYC1
FS3
sh RNA CHCHD3
STRATAGENE

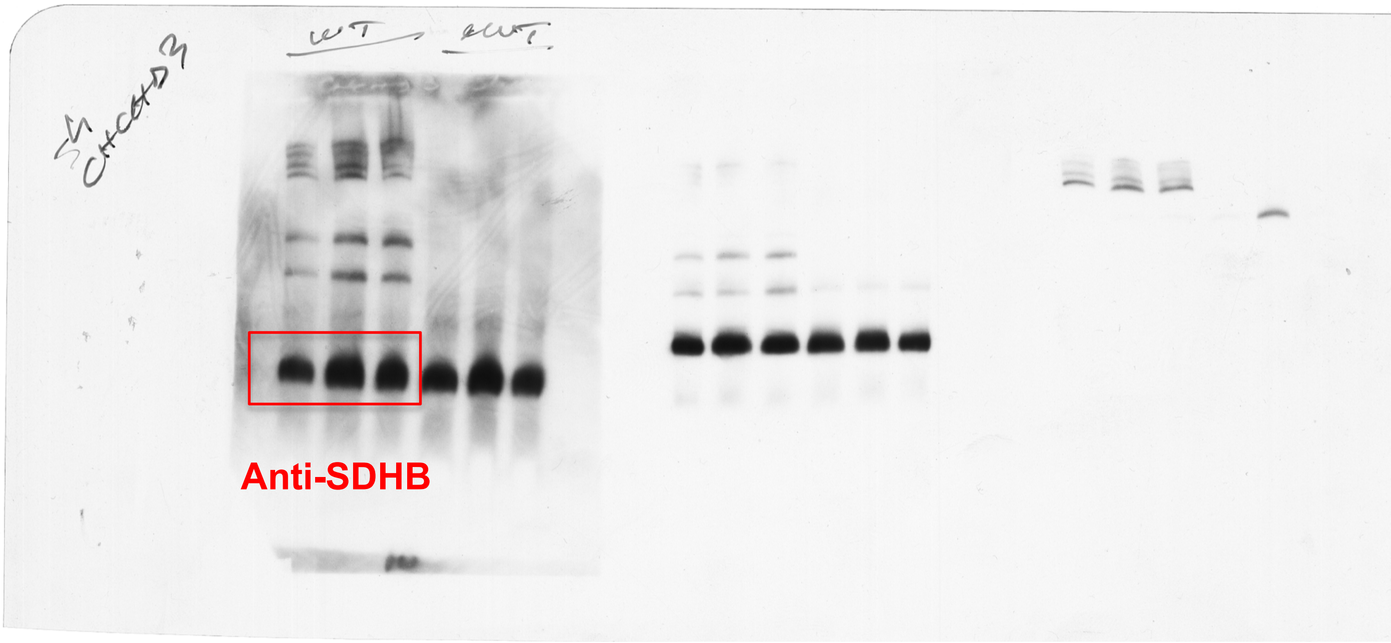
sh CHCHD3
WT
Anti-SDHB
