# Supplementary figures and images for: Respiratory supercomplexes act as a platform for complex III‐mediated maturation of human mitochondrial complexes I and IV
Source: EMBO J. 2020 Jan 8;39(3):e102817. doi: 10.15252/embj.2019102817 (PMC6996572; doi:10.15252/embj.2019102817)

Figure EV5B – Anti-NDUFAF2

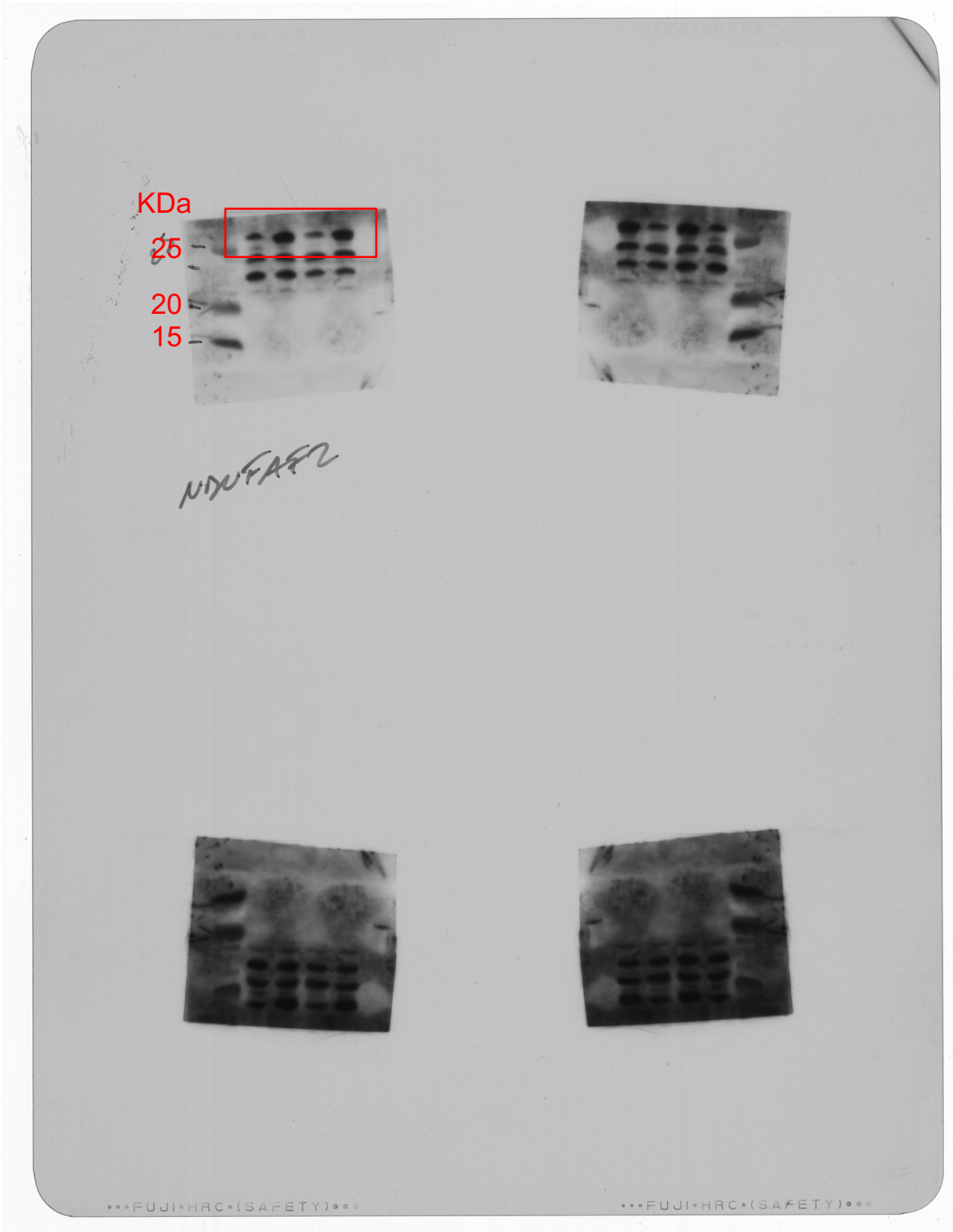

### Figure EV5B – Anti-FLAG & Anti-β-Tubulin

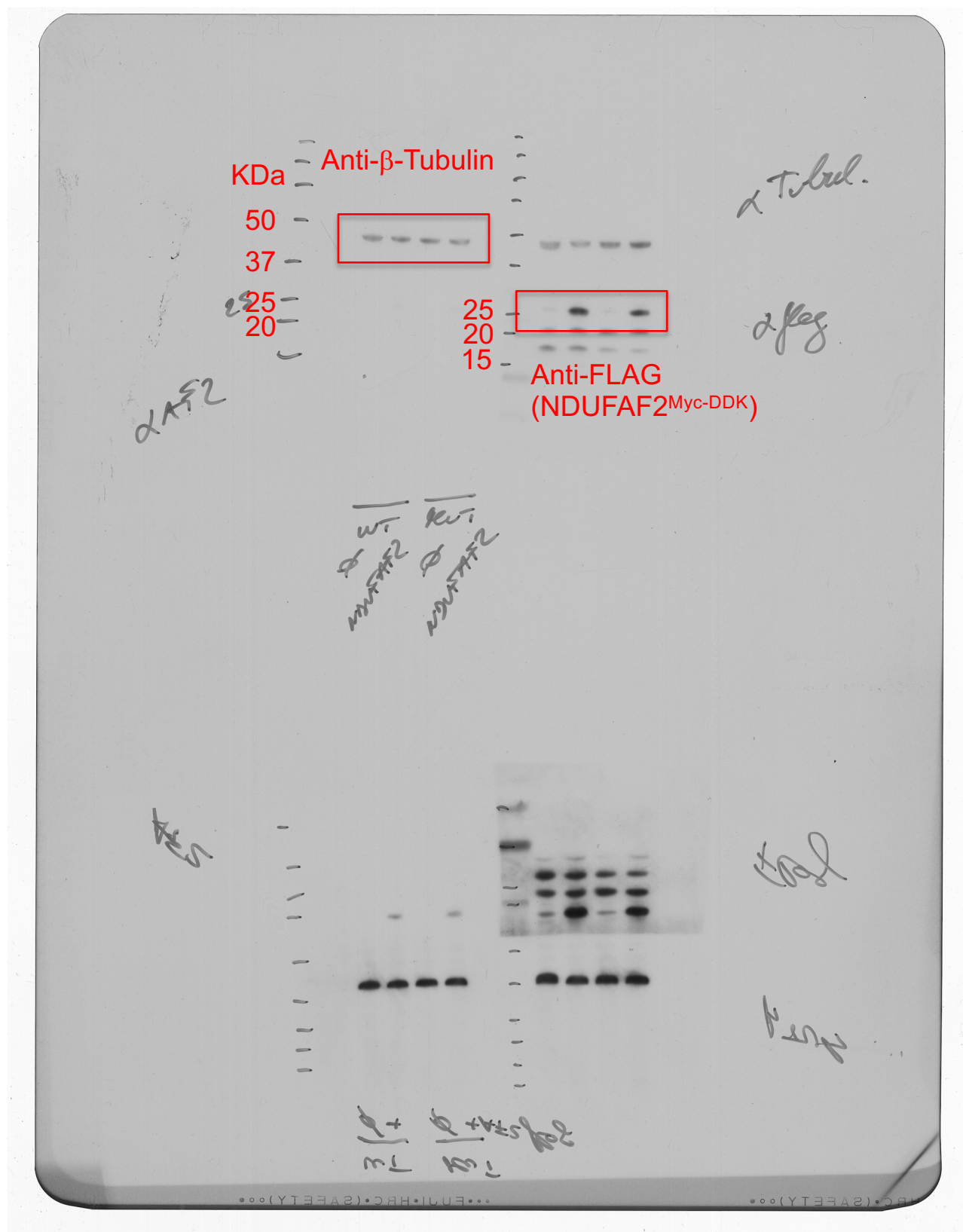

Figure EV5C

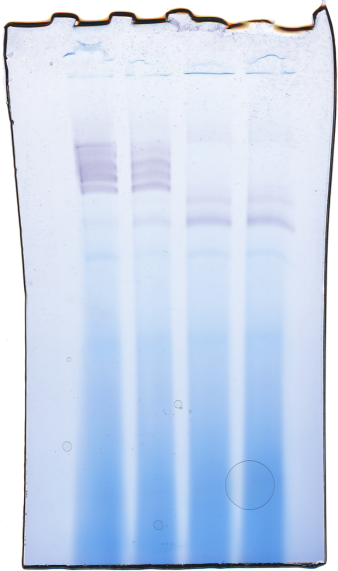

Figure EV5E

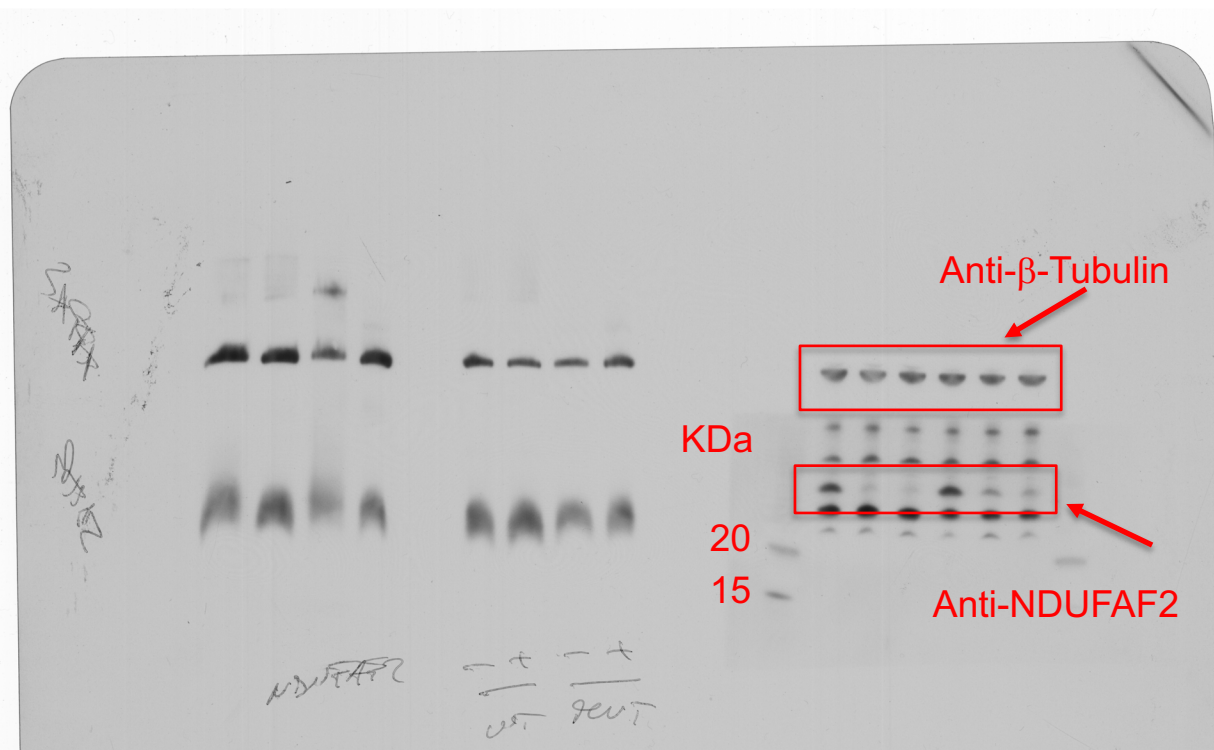

Figure EV5F

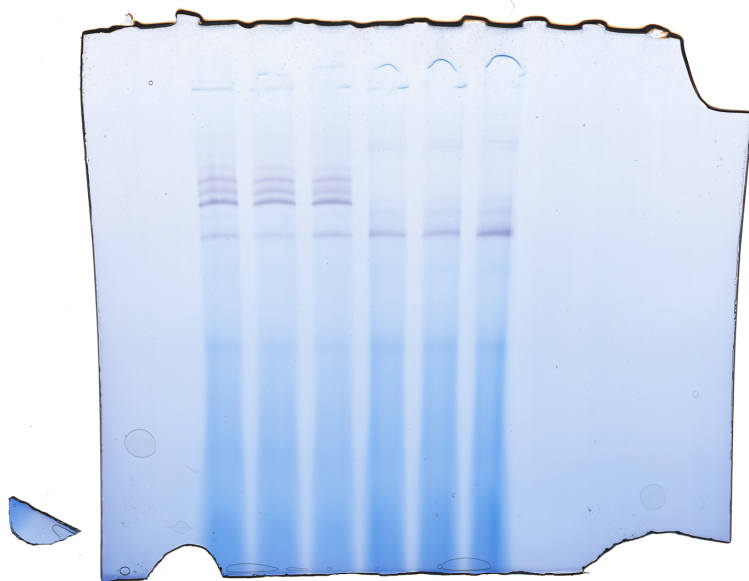

Supplement: Supplementary file 2 — Source Data for Expanded View [file EMBJ-39-e102817-s009.zip › Figure_EV5_Source_Data.pdf]

Figure 1B

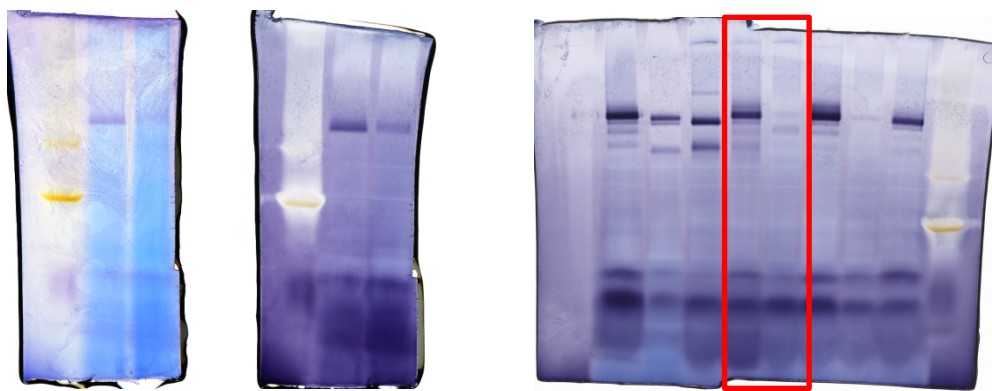

Figure 1C

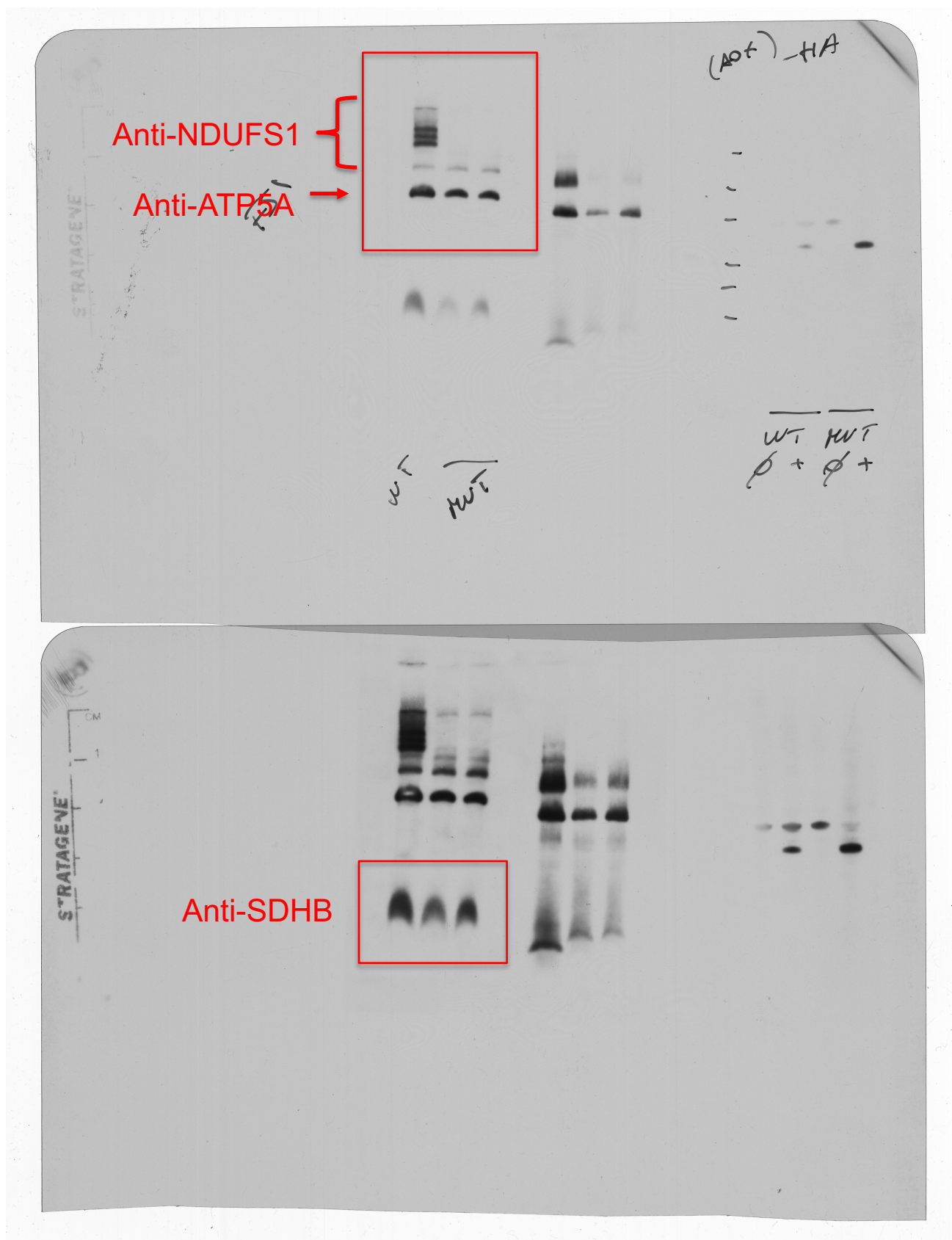

Supplement: Supplementary file 4 — Source Data for Figure 1 [file EMBJ-39-e102817-s002.pdf]

Figure 3B

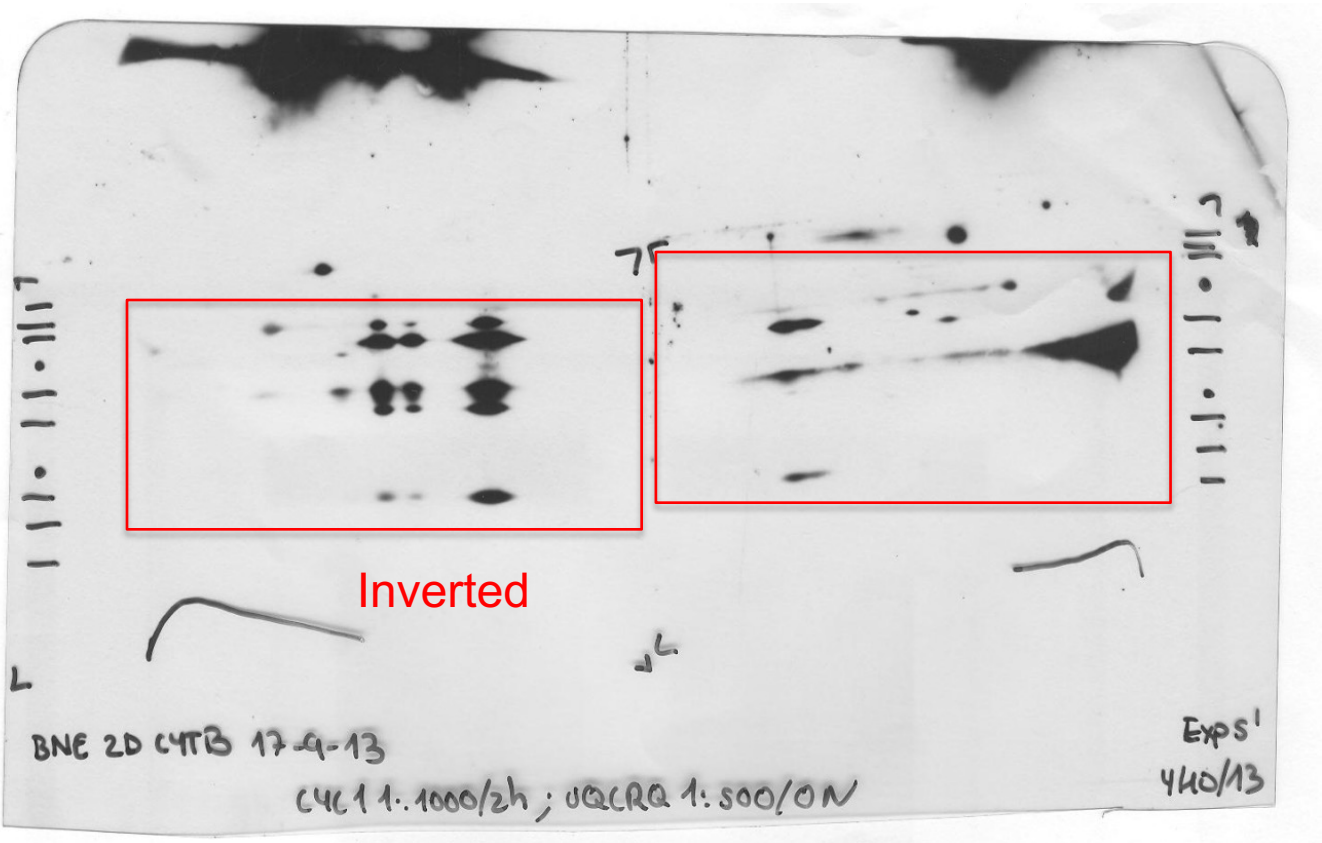

Supplement: Supplementary file 6 — Source Data for Figure 3 [file EMBJ-39-e102817-s004.pdf]

Figure 8D

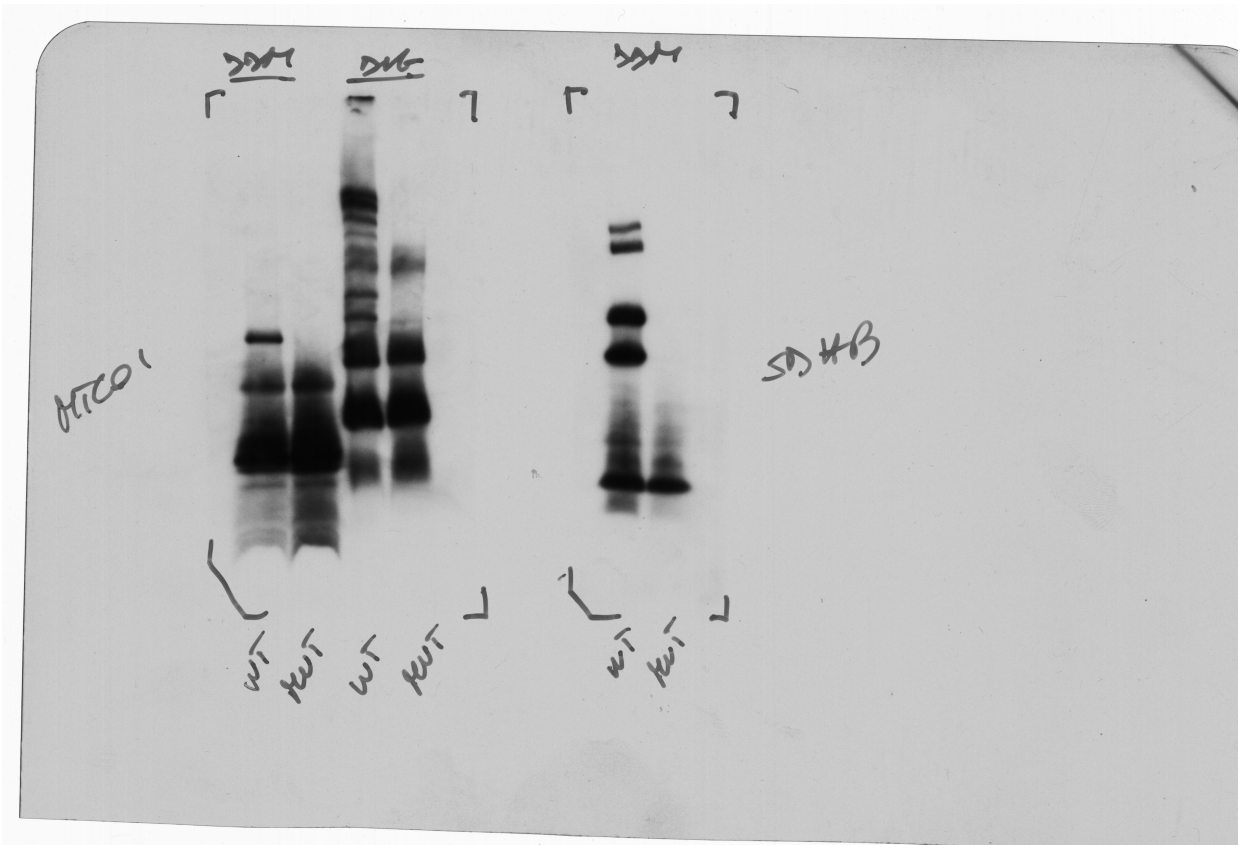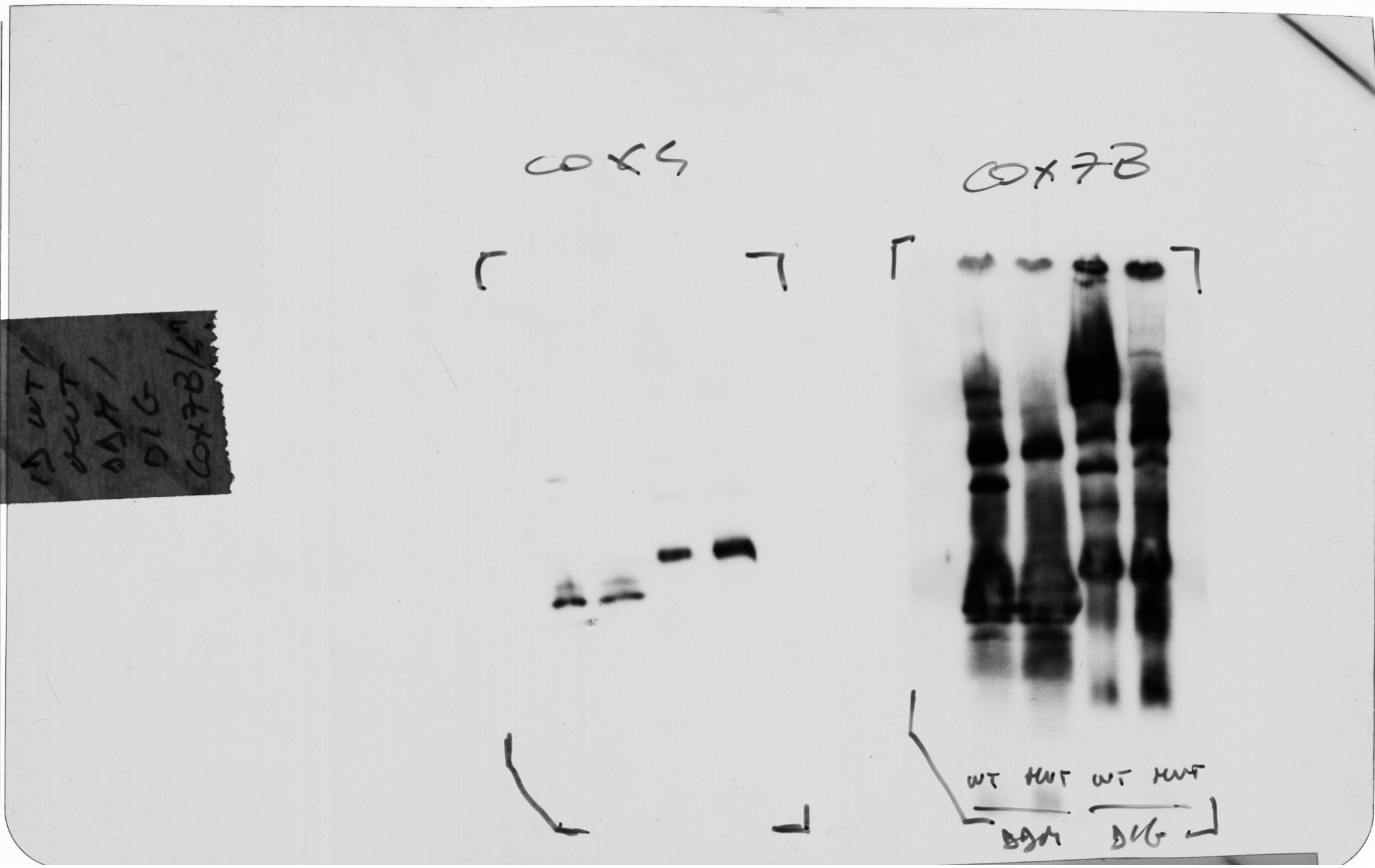

Supplement: Supplementary file 10 — Source Data for Figure 8 [file EMBJ-39-e102817-s008.pdf]
