# Supplementary material for: Respiratory supercomplexes act as a platform for complex III‐mediated maturation of human mitochondrial complexes I and IV
Source: EMBO J. 2020 Jan 8;39(3):e102817. doi: 10.15252/embj.2019102817 (PMC6996572; doi:10.15252/embj.2019102817)
Supplement: Supplementary file 5 — Source Data for Figure 2 [file EMBJ-39-e102817-s003.pdf]

Figure 2B

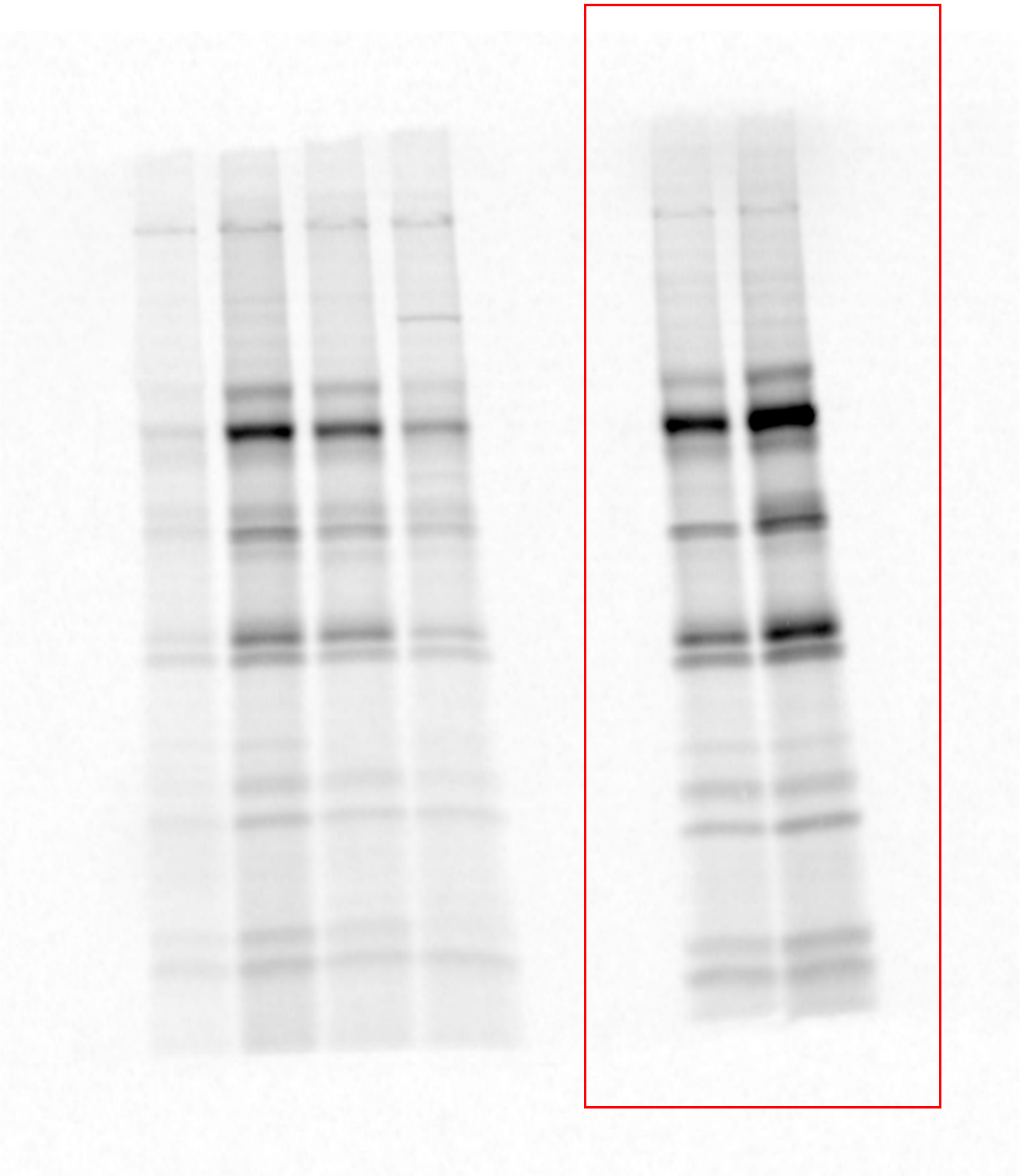

FIGURE 2C

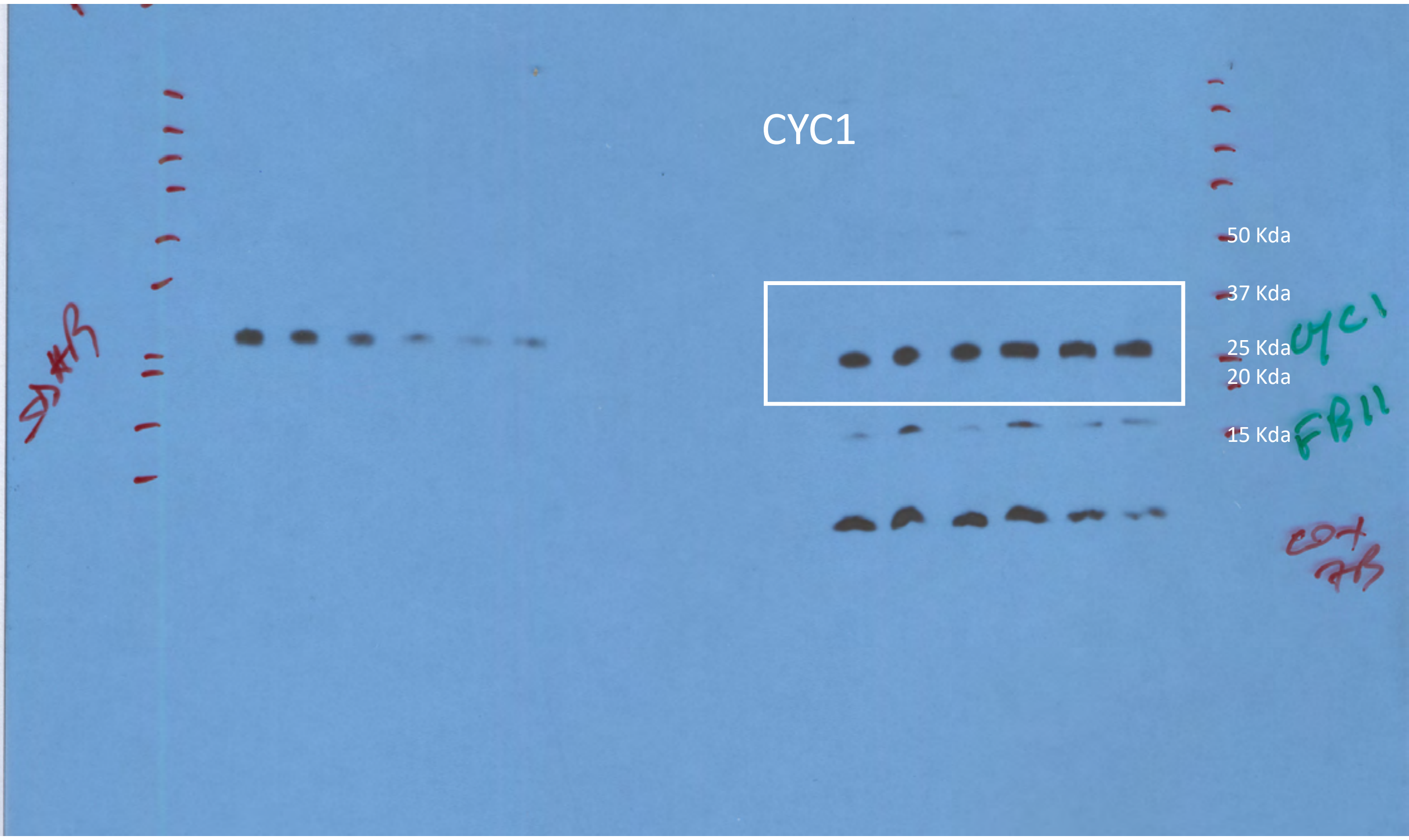

FIGURE 2C

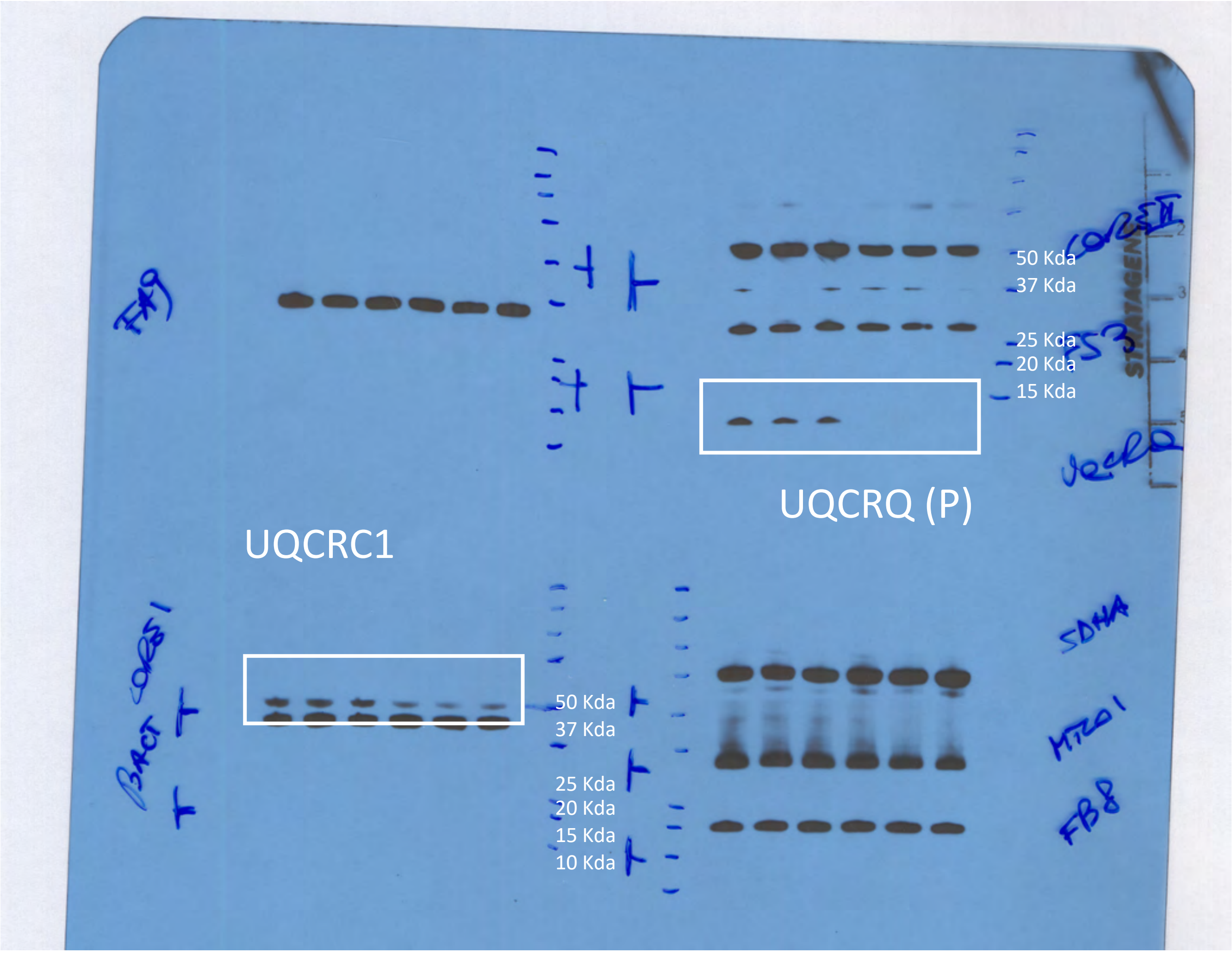
50 Kda
37 Kda
25 Kda
20 Kda
15 Kda
UQCRQ (P)
UQCRC1
50 Kda
37 Kda
25 Kda
20 Kda
15 Kda
10 Kda

FIGURE 2C

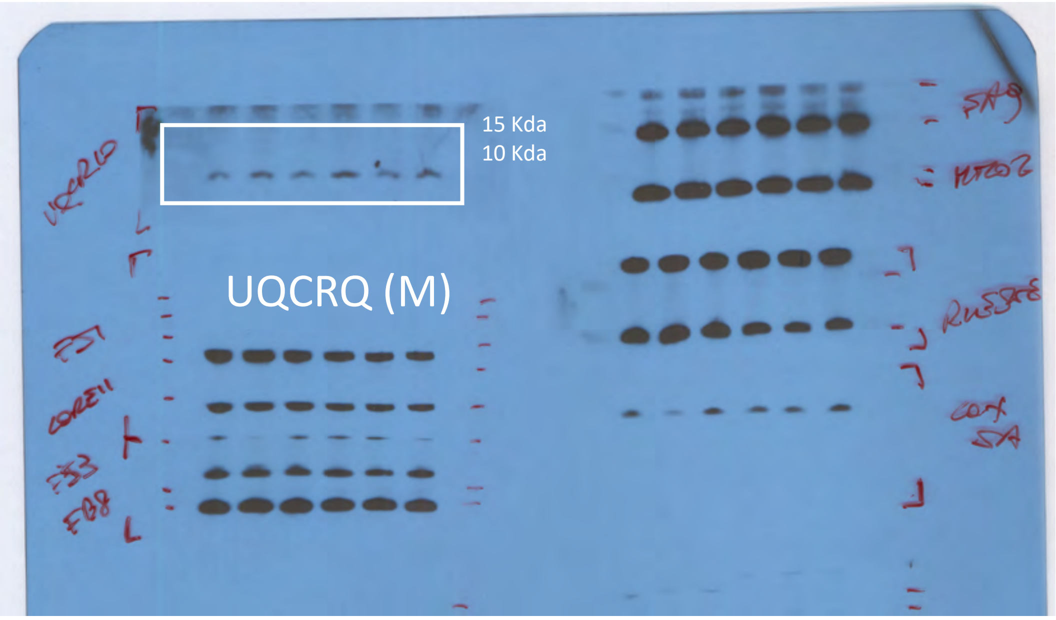

15 Kda
10 Kda
UQCRQ (M)

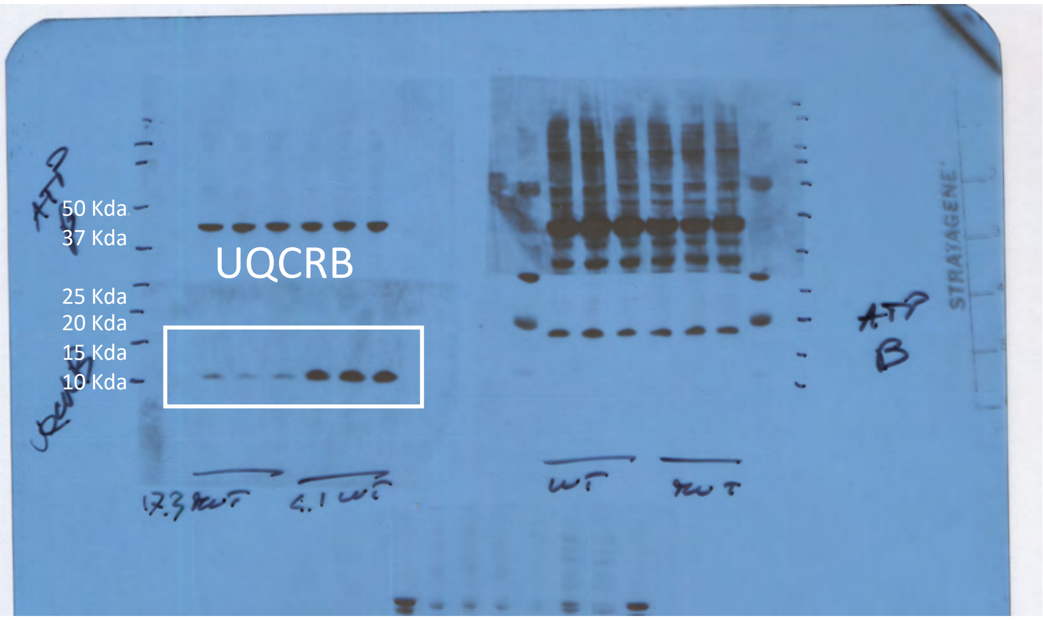

50 Kda
37 Kda
UQCRB
25 Kda
20 Kda
15 Kda
10 Kda
STRATAGENE

FIGURE 2C

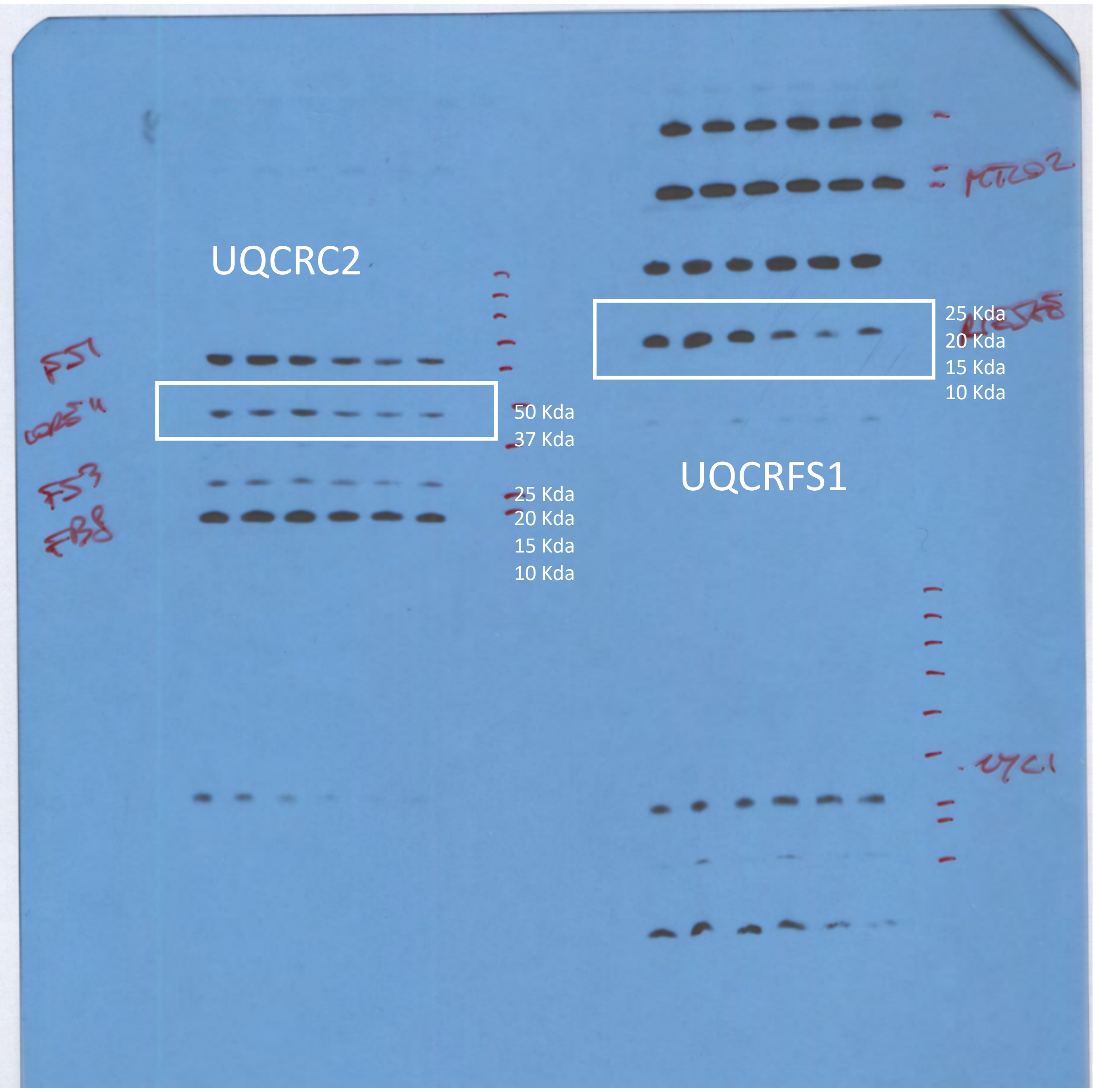
UQCRC2
50 Kda
37 Kda
25 Kda
20 Kda
15 Kda
10 Kda
UQCRFS1
25 Kda
20 Kda
15 Kda
10 Kda

FIGURE 2C

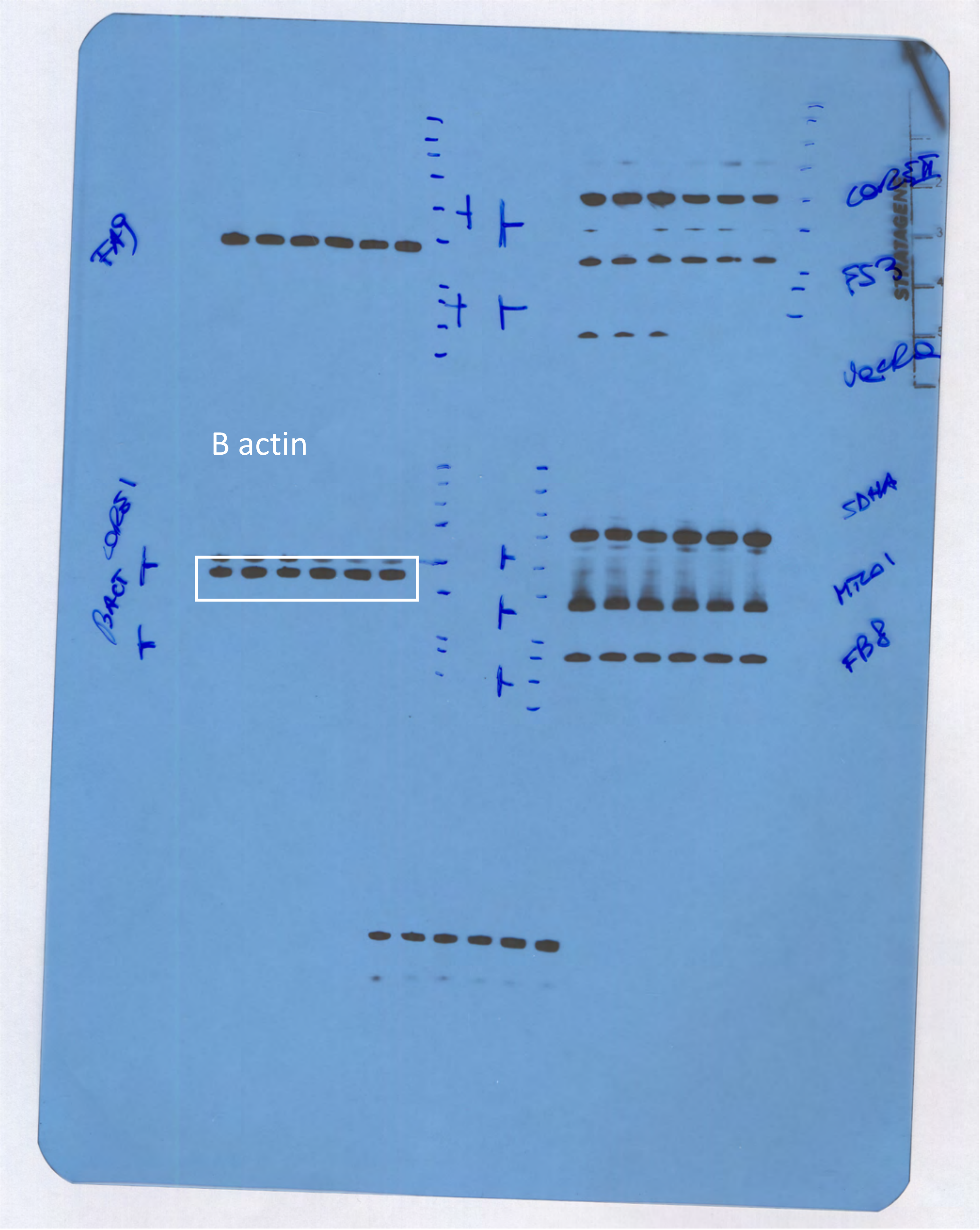

FIGURE 2D

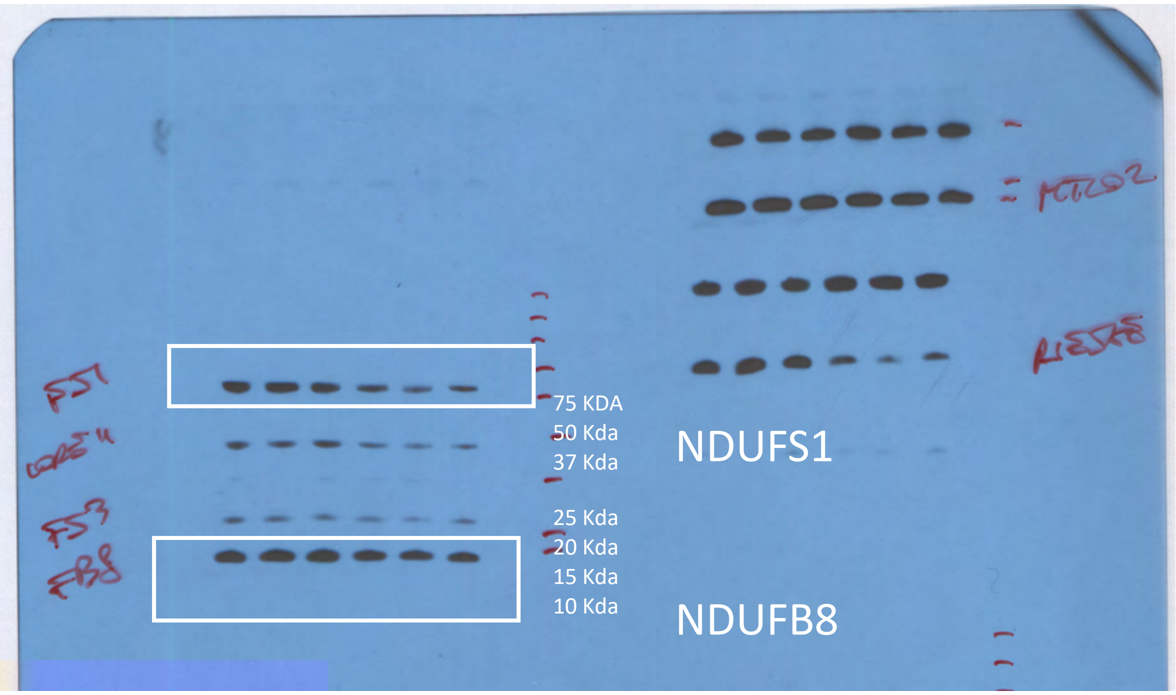
75 KDA
50 Kda
37 Kda
25 Kda
20 Kda
15 Kda
10 Kda
NDUFS1
NDUFB8

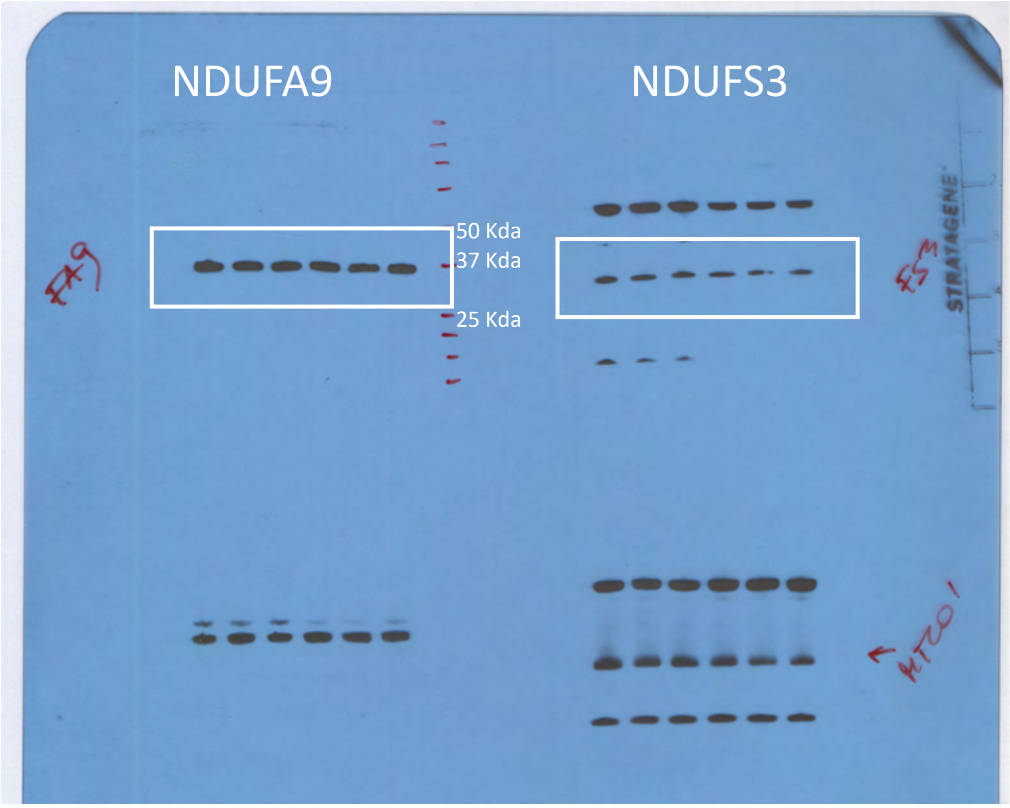
NDUFA9
NDUFS3
50 Kda
37 Kda
25 Kda
STRATAGENE

FIGURE 2D

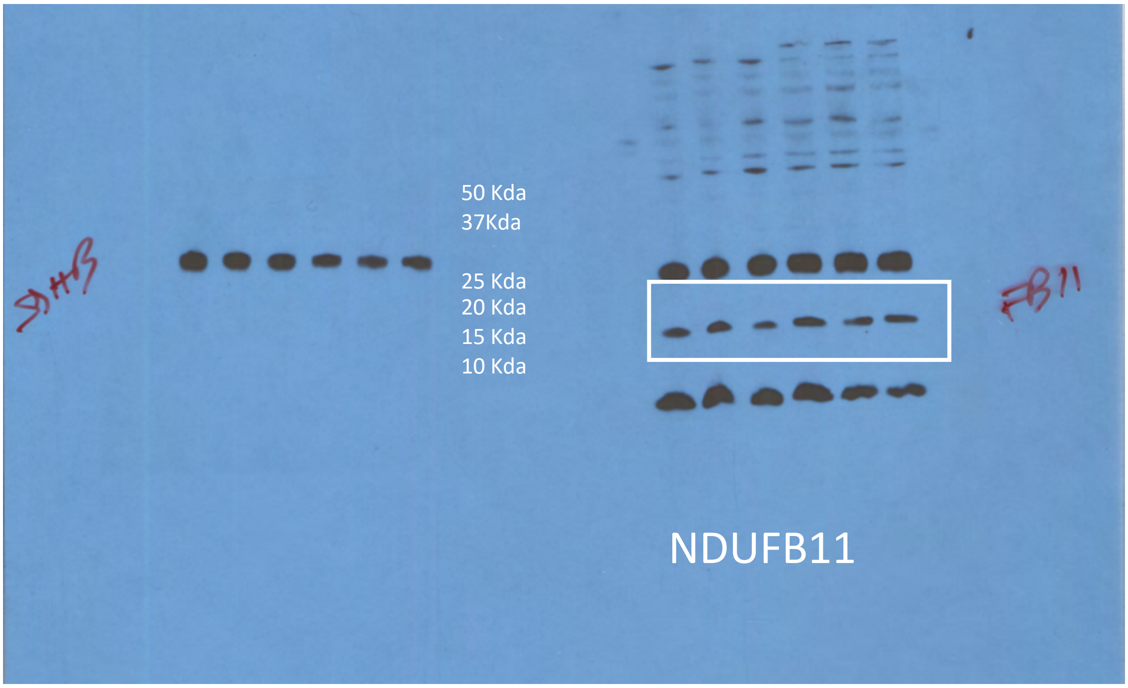
50 Kda
37Kda
25 Kda
20 Kda
15 Kda
10 Kda
NDUFB11

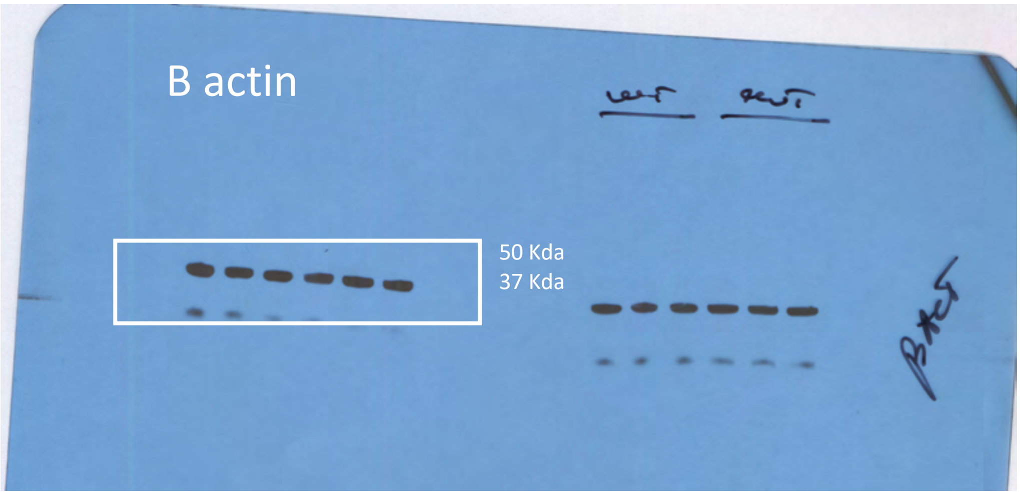
B actin
50 Kda
37 Kda

FIGURE 2E

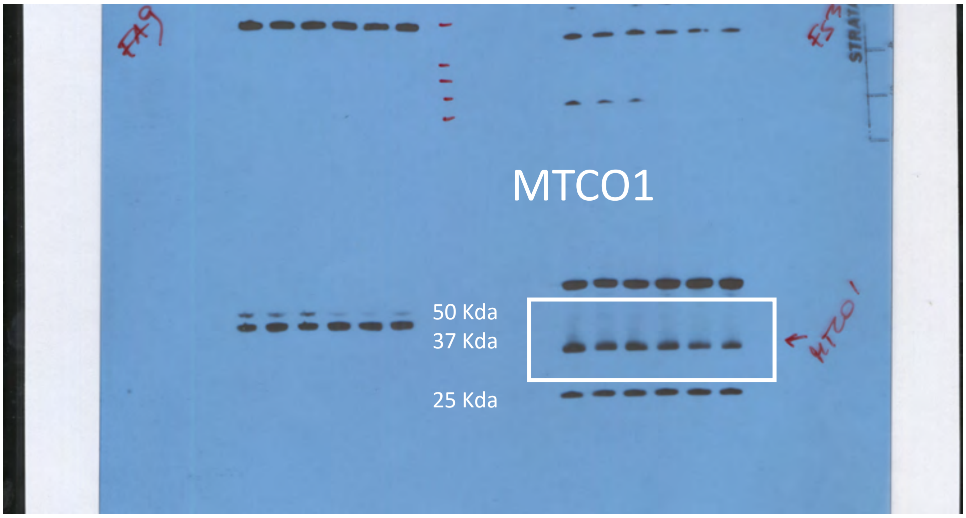
MTCO1
50 Kda
37 Kda
25 Kda

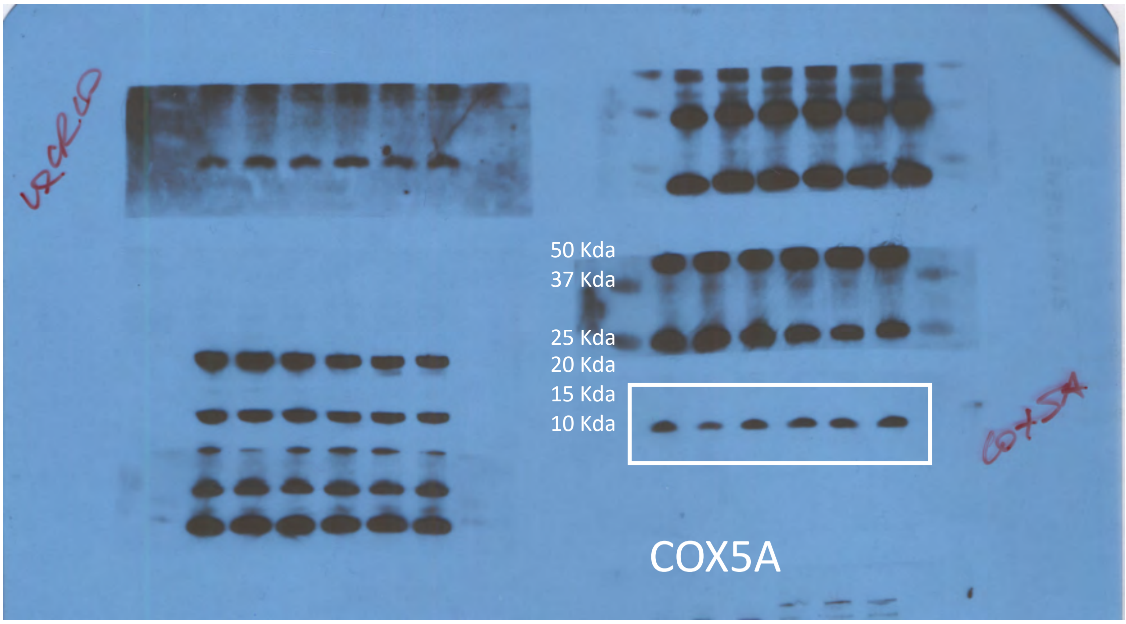
50 Kda
37 Kda
25 Kda
20 Kda
15 Kda
10 Kda
COX5A

FIGURE 2E

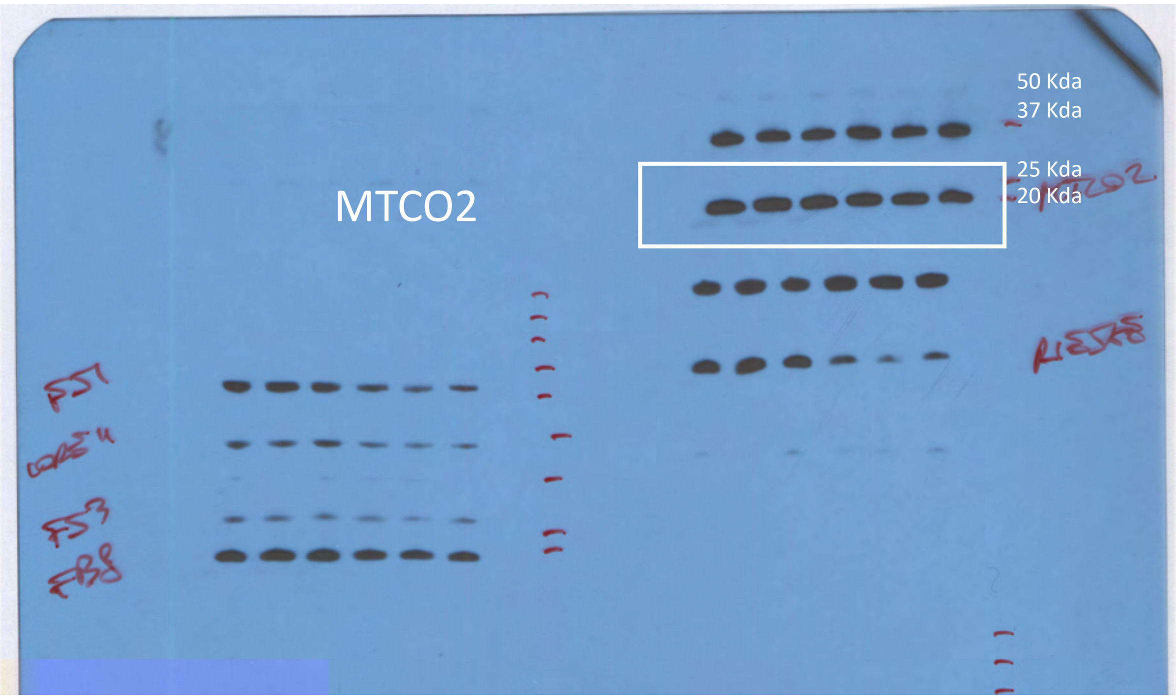

MTCO2
50 Kda
37 Kda
25 Kda
20 Kda

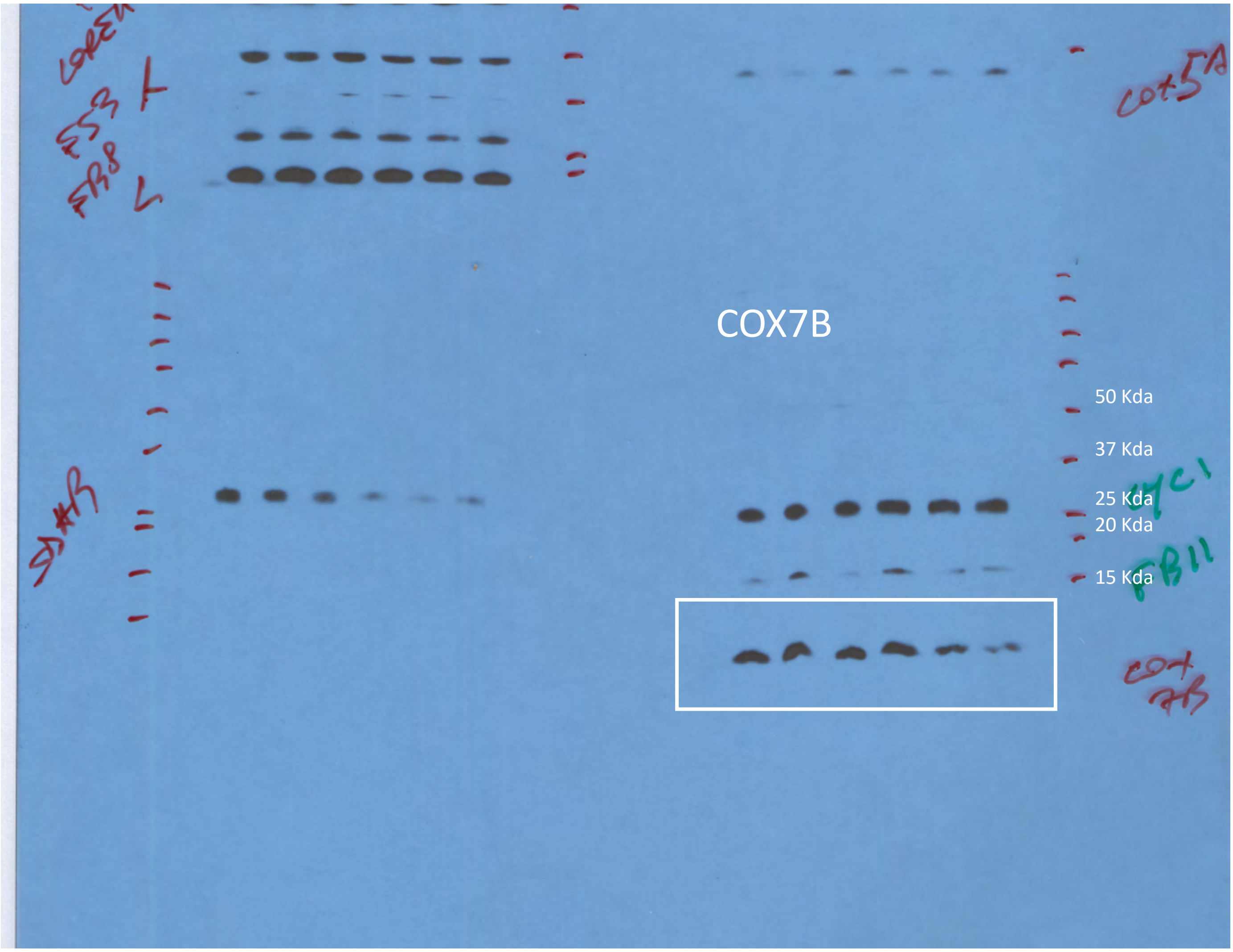

COX7B
50 Kda
37 Kda
25 Kda
20 Kda
15 Kda

FIGURE 2E

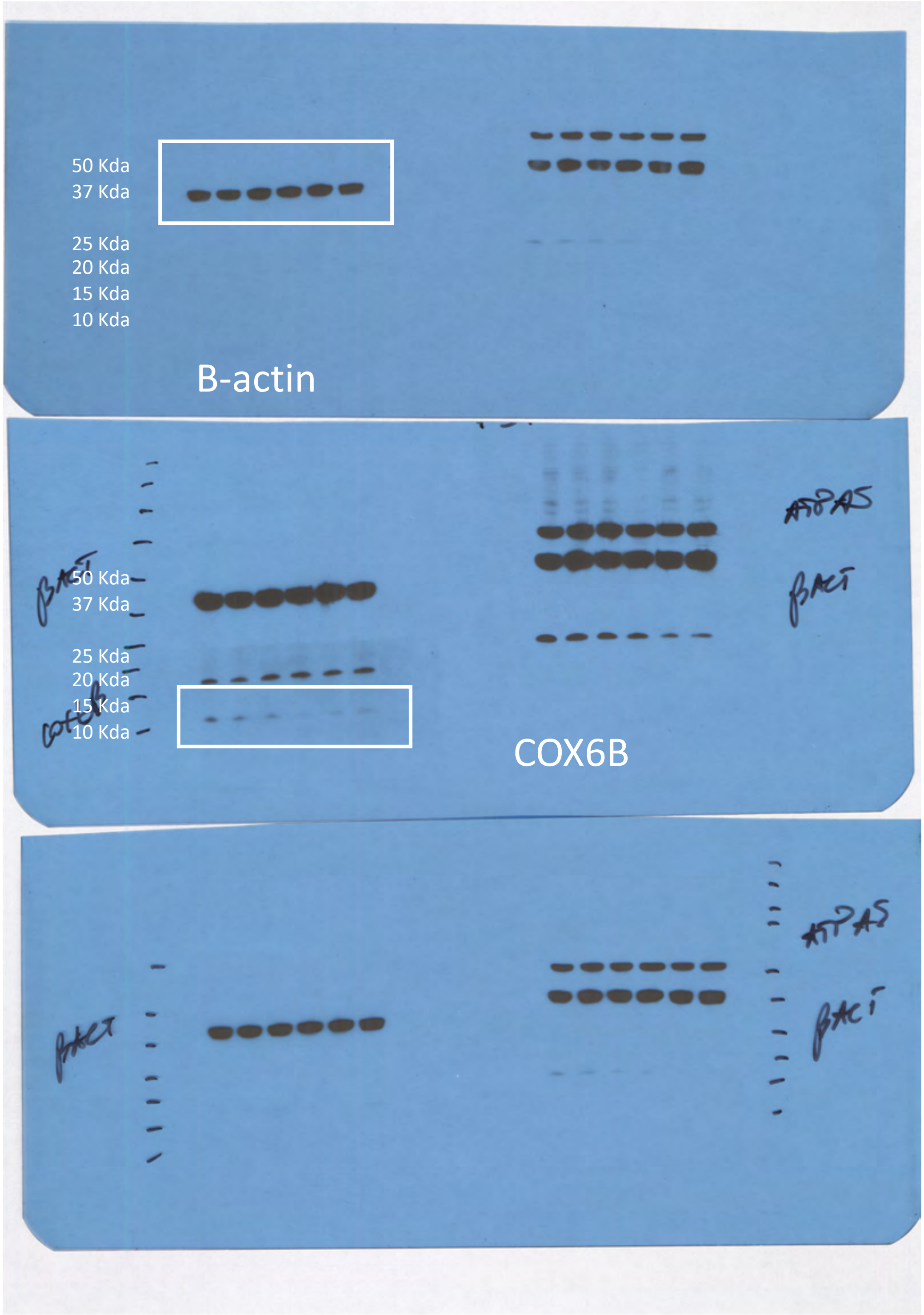
50 Kda
37 Kda
25 Kda
20 Kda
15 Kda
10 Kda
B-actin
50 Kda
37 Kda
25 Kda
20 Kda
15 Kda
10 Kda
COX6B

FIGURE 2F

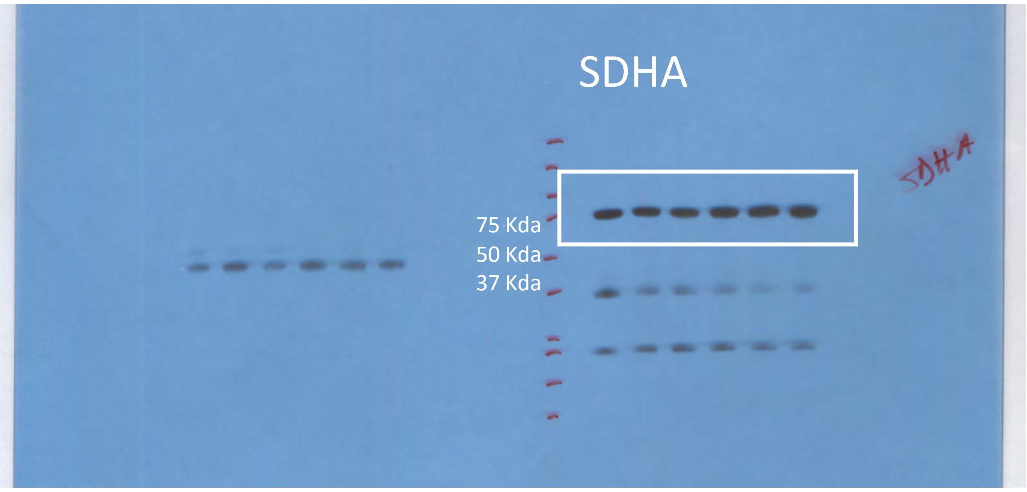
SDHA
75 Kda
50 Kda
37 Kda

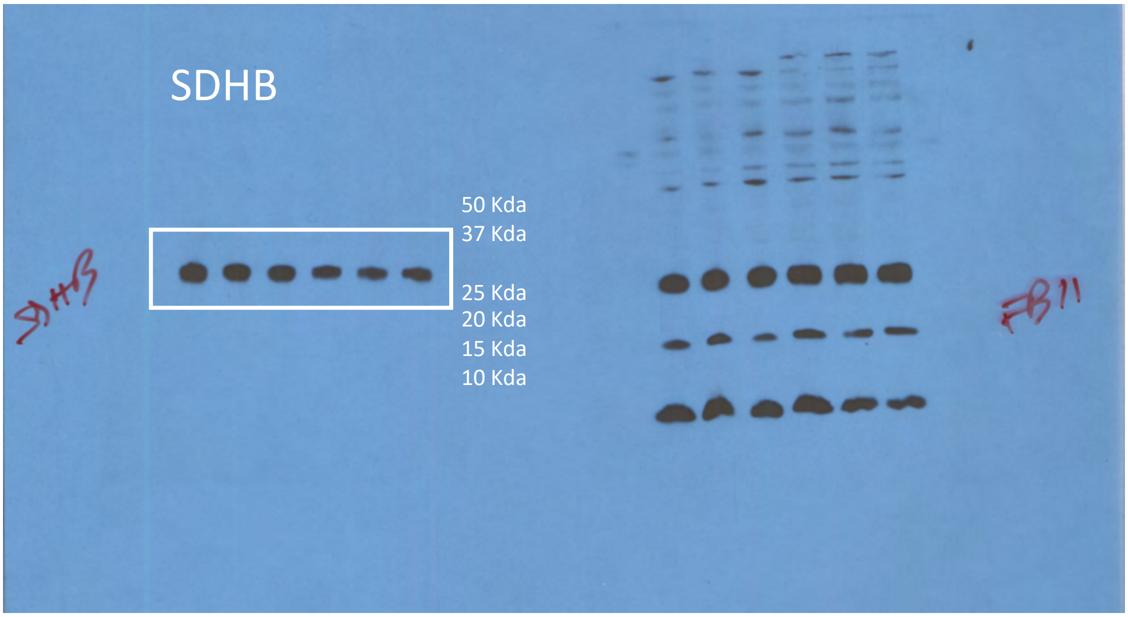
SDHB
50 Kda
37 Kda
25 Kda
20 Kda
15 Kda
10 Kda

FIGURE 2G

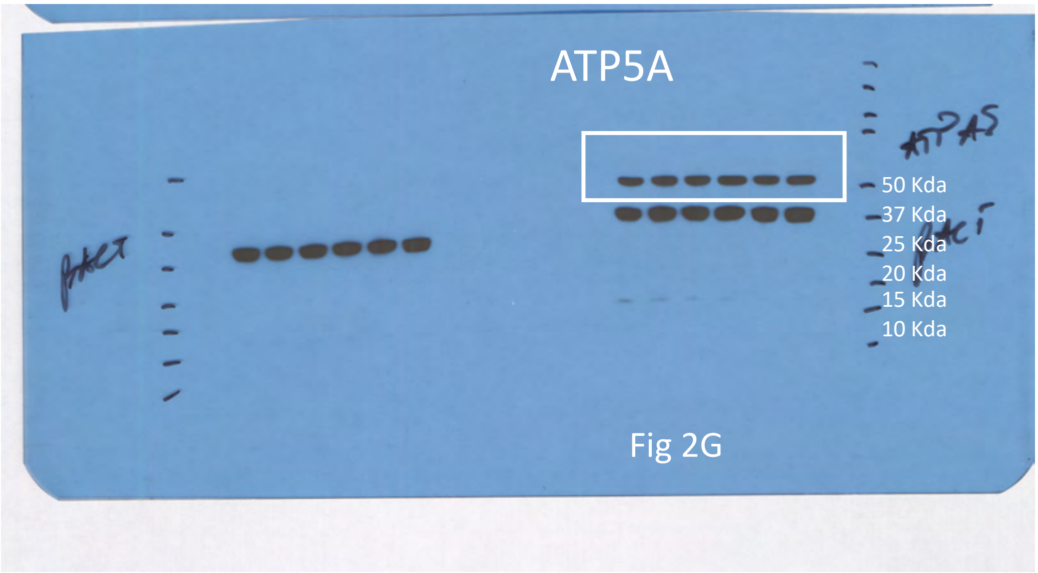
ATP5A
50 Kda
37 Kda
25 Kda
20 Kda
15 Kda
10 Kda
Fig 2G

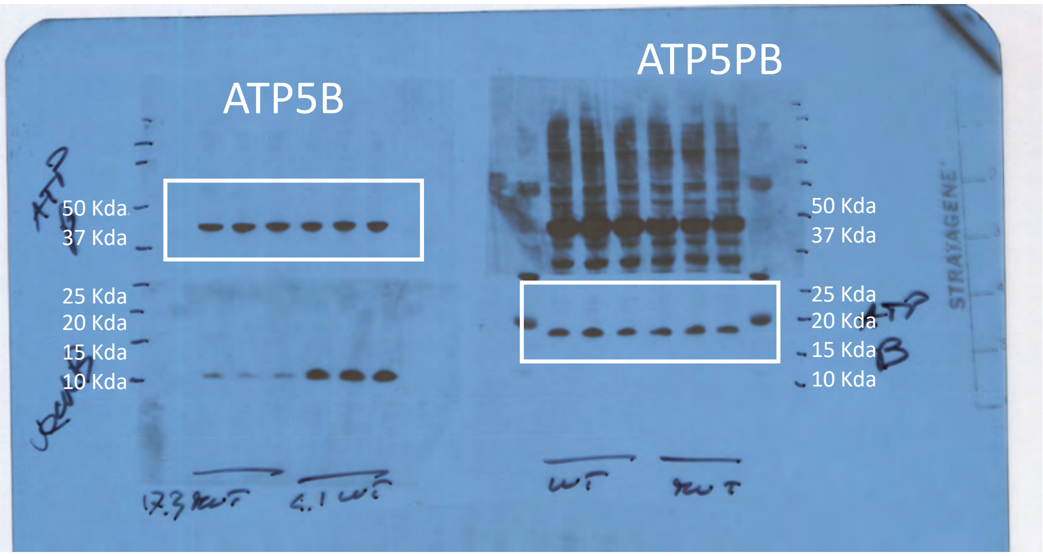
ATP5B
ATP5PB
50 Kda
37 Kda
25 Kda
20 Kda
15 Kda
10 Kda
50 Kda
37 Kda
25 Kda
20 Kda
15 Kda
10 Kda
STRATAGENE

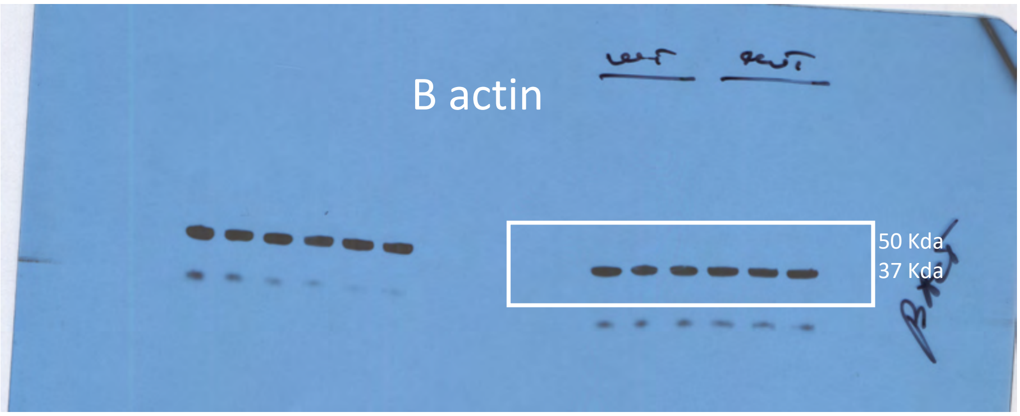
B actin
50 Kda
37 Kda
