# Supplementary material for: Respiratory supercomplexes act as a platform for complex III‐mediated maturation of human mitochondrial complexes I and IV
Source: EMBO J. 2020 Jan 8;39(3):e102817. doi: 10.15252/embj.2019102817 (PMC6996572; doi:10.15252/embj.2019102817)

Figure 4A – Anti-HA

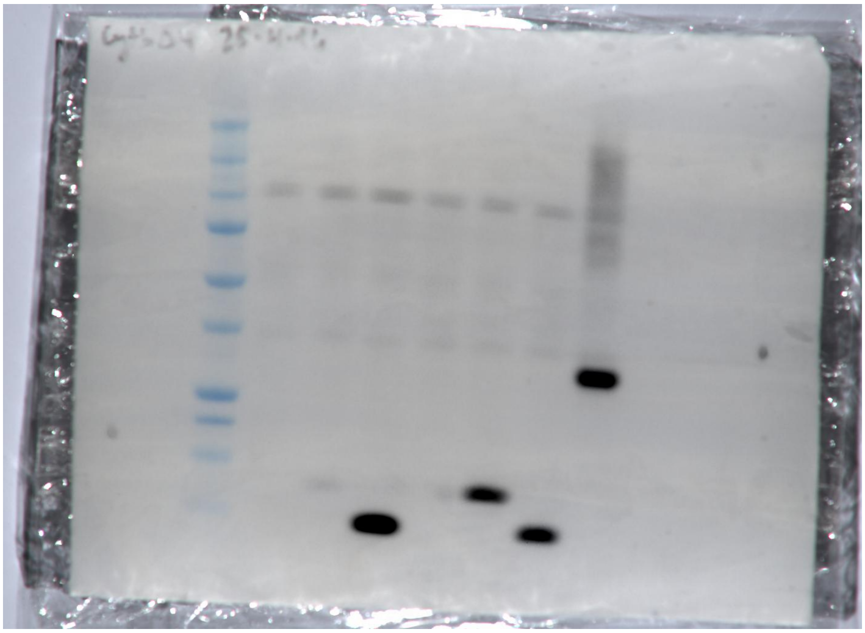

Figure 4A – Anti-CYC1

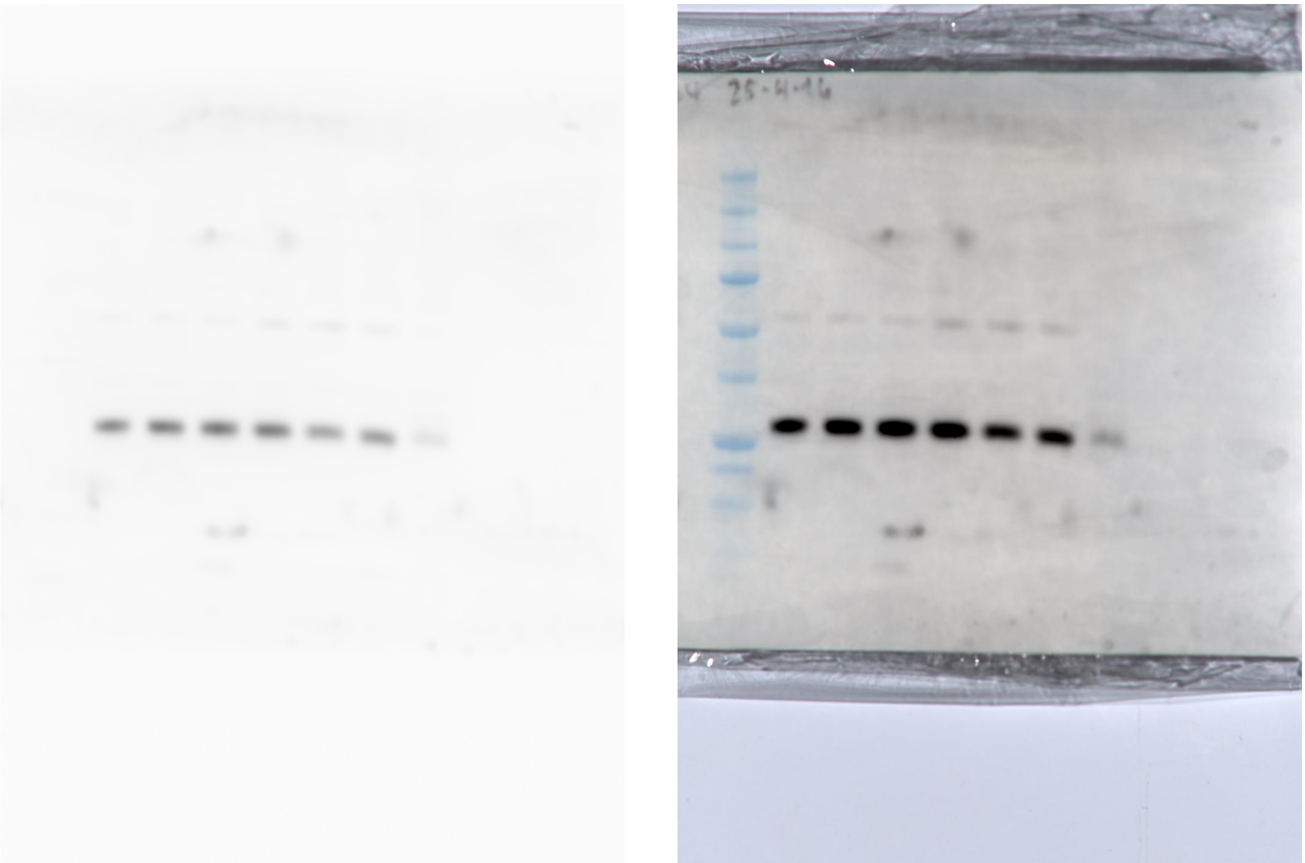

Figure 4A – Anti-Tubulin

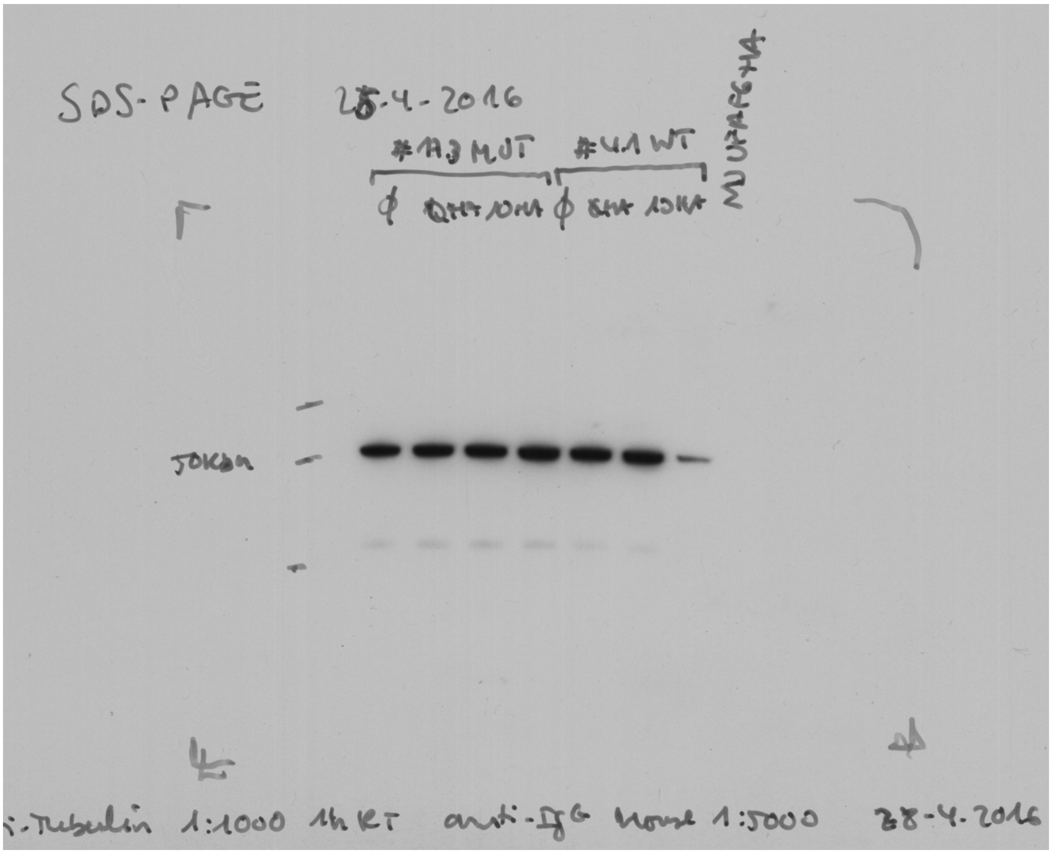

Figure 4B

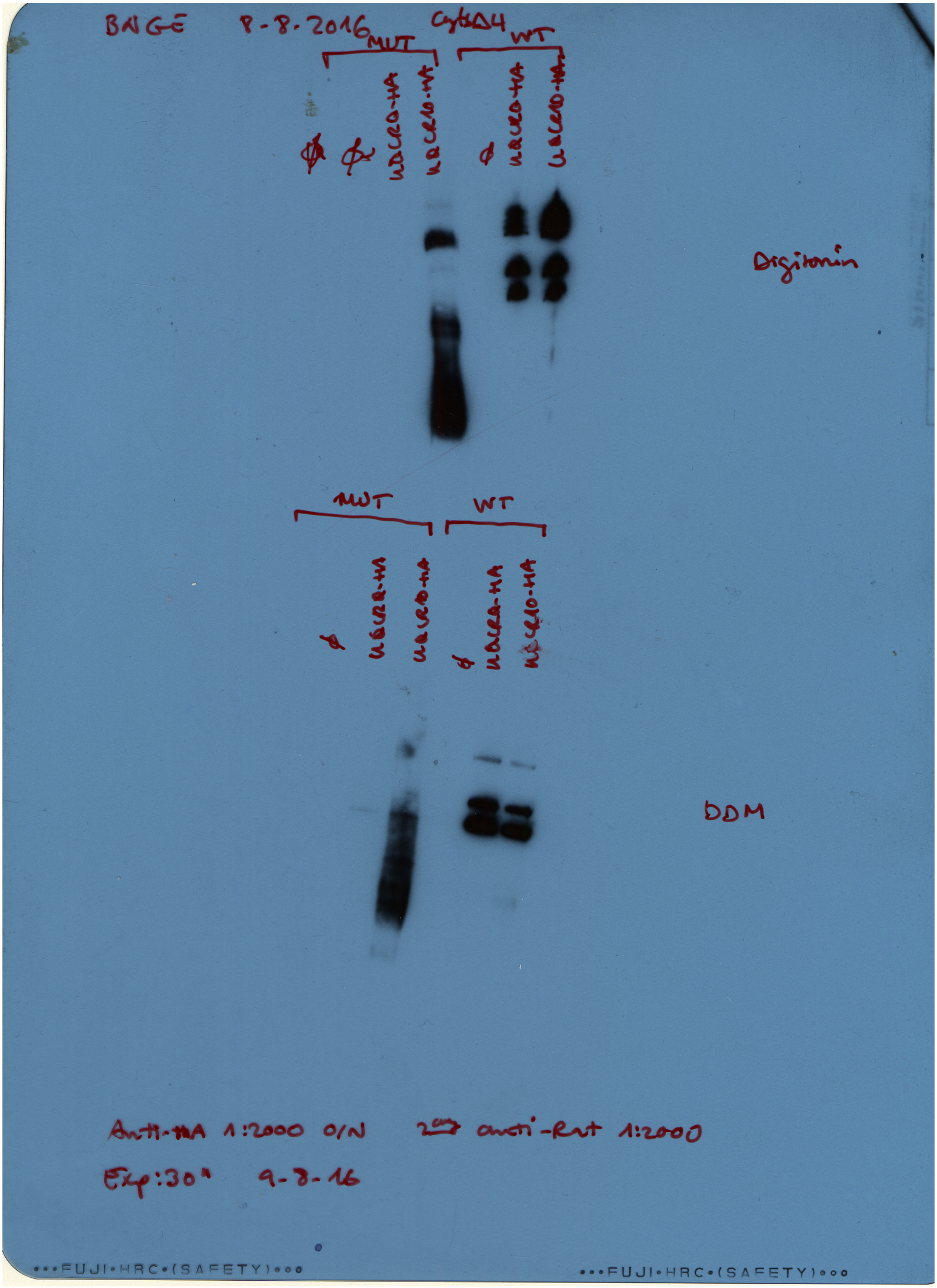
BNGE 8-8-2016
MUT
WT
Digitonin
MUT
WT
DDM
Anti-HA 1:2000 O/N
anti-Rat 1:2000
Exp: 30"
9-8-16
•••FUJI•HRC•(SAFETY)•••
•••FUJI•HRC•(SAFETY)•••

Figure 4C

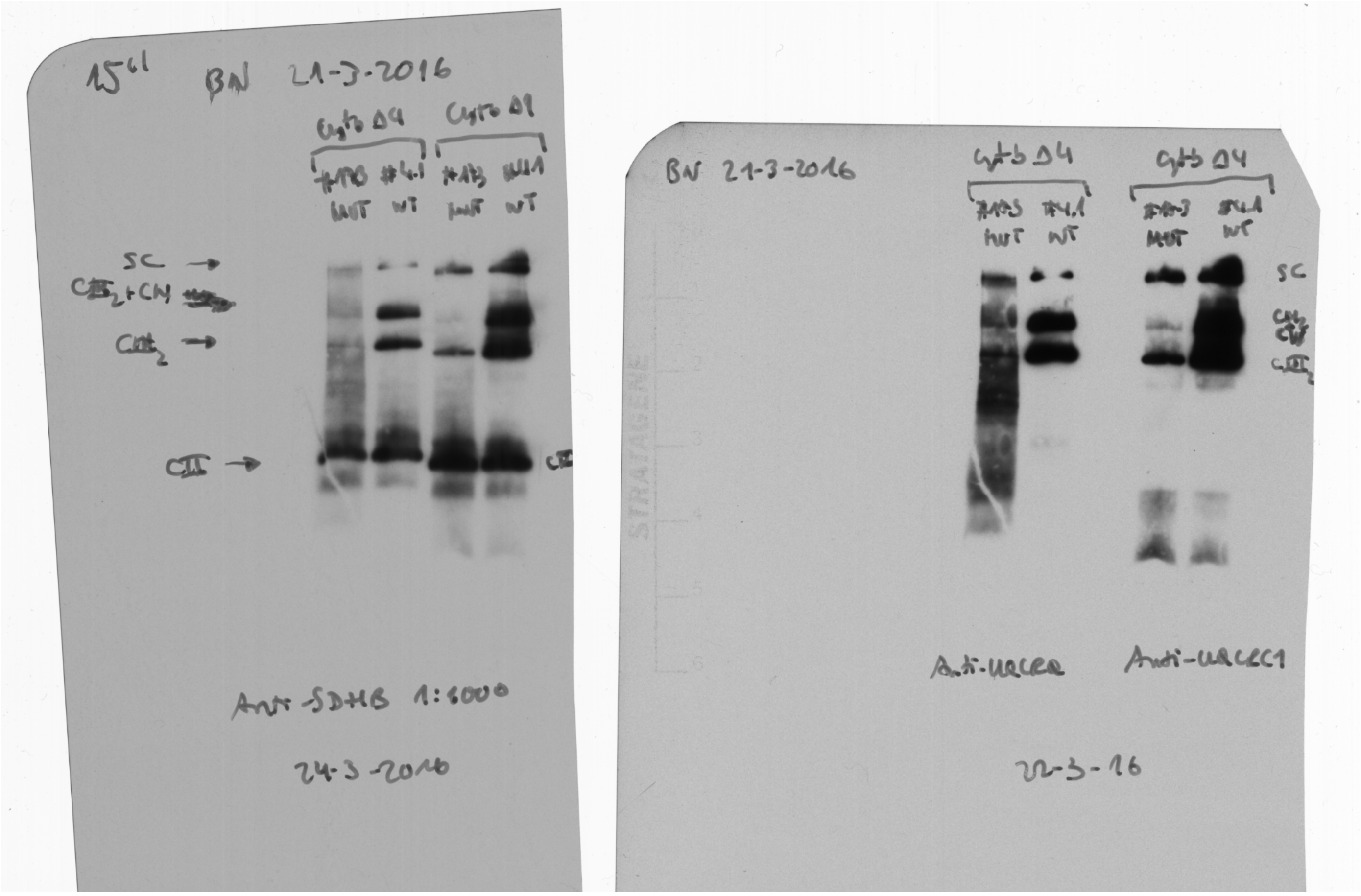

Figure 4F

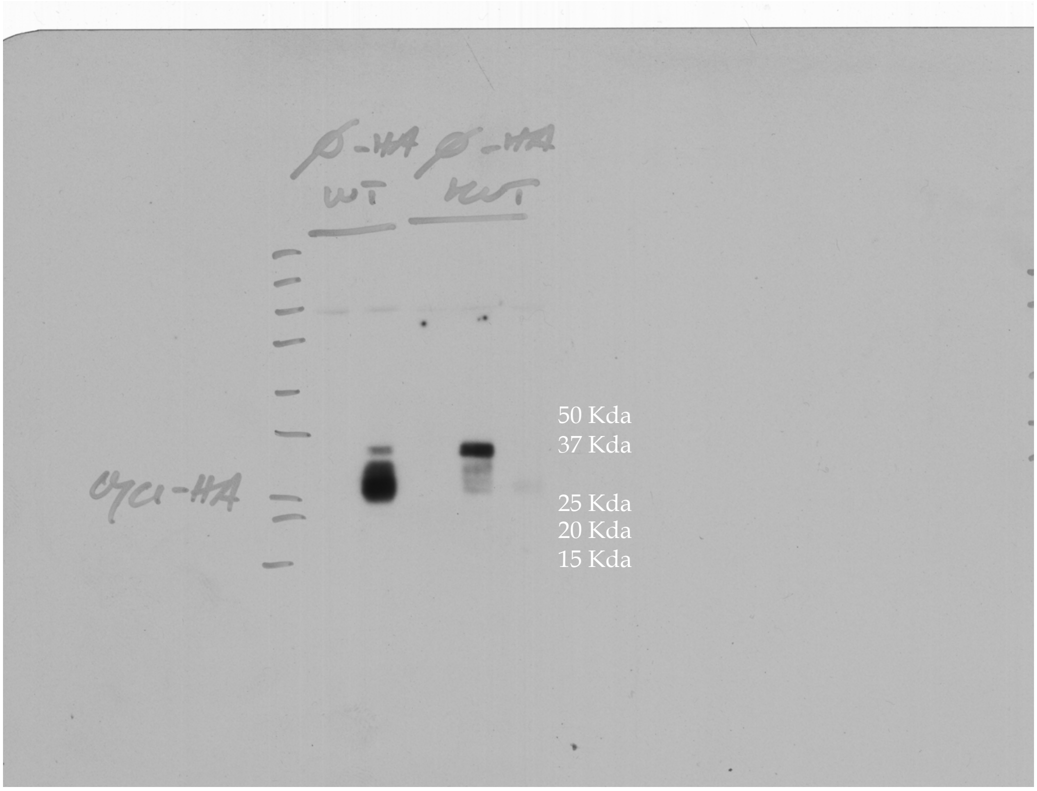

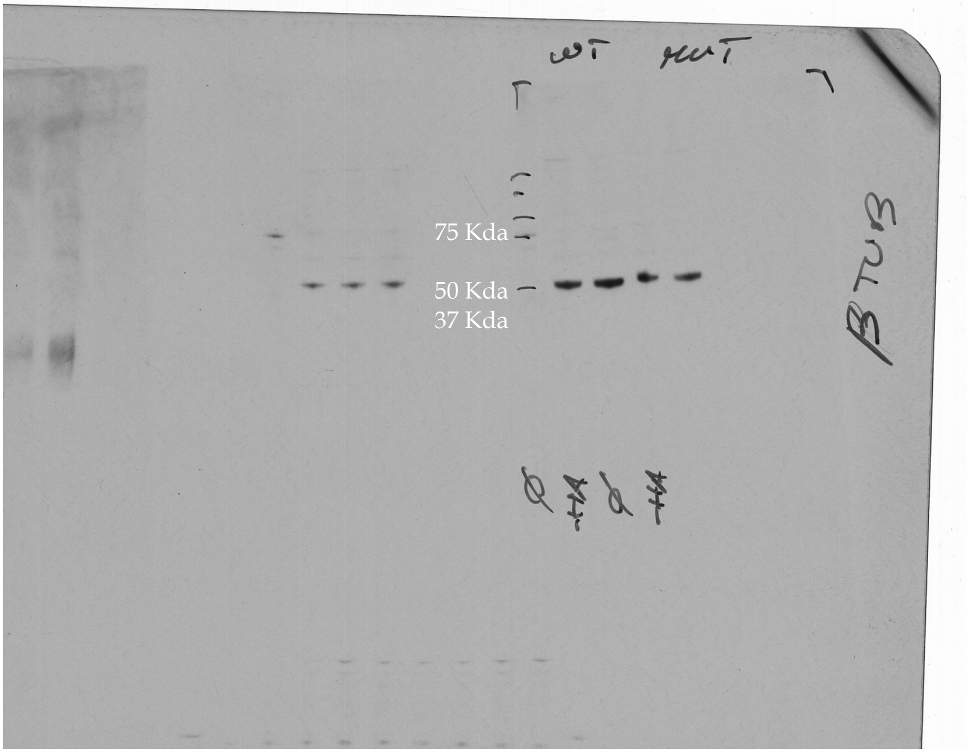

Figure 4G – Anti-CYC1

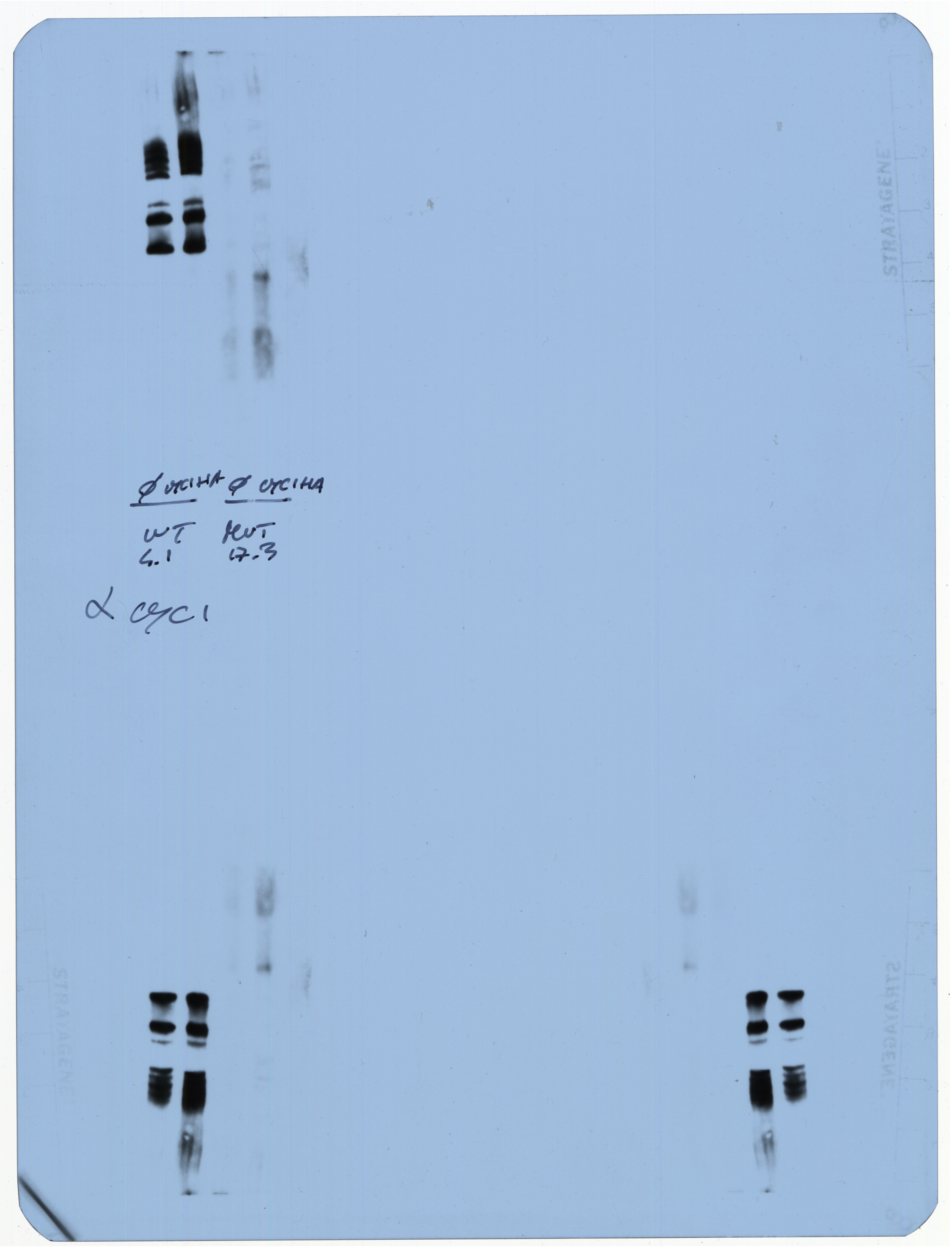

Figure 4G – Anti-HA

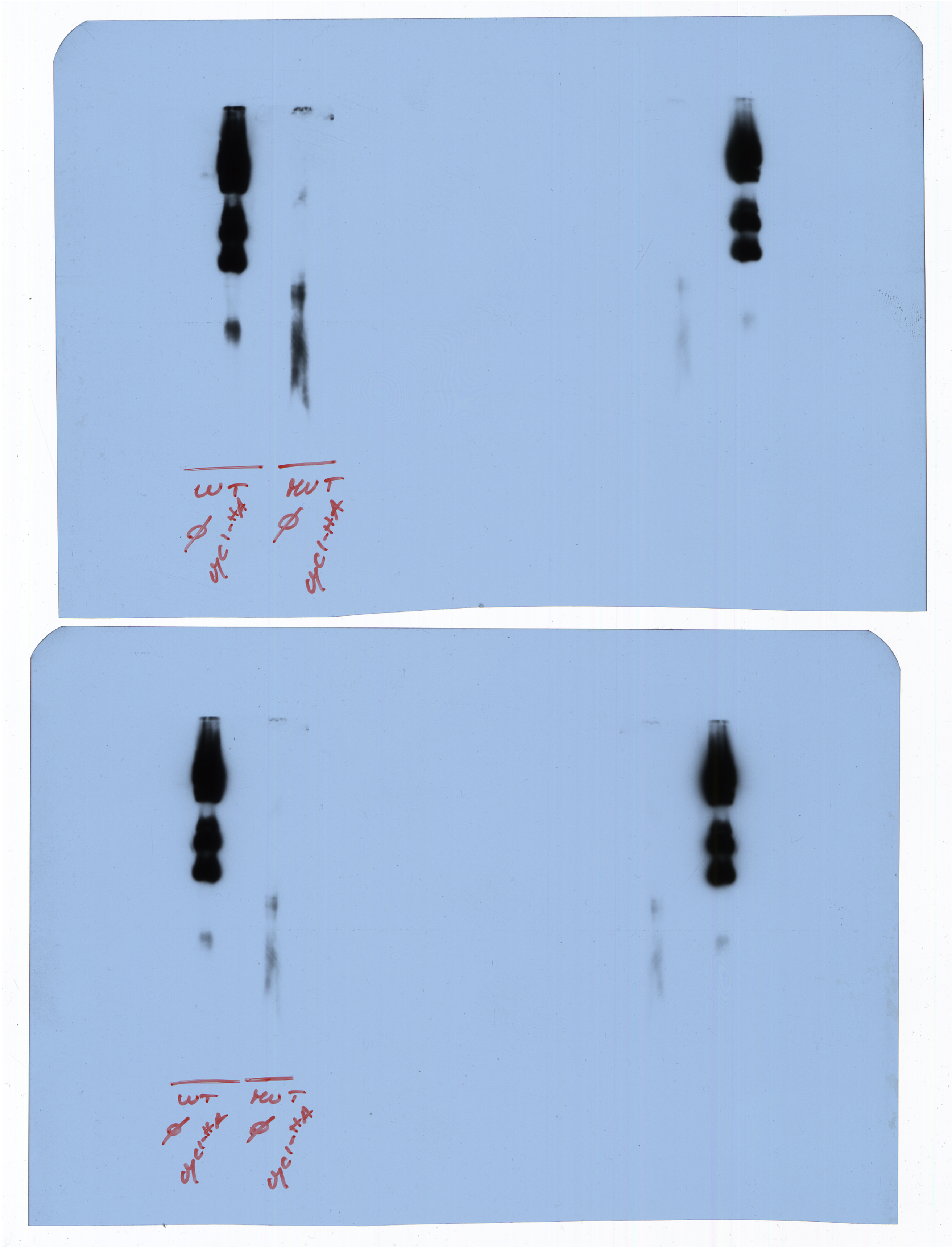

Supplement: Supplementary file 7 — Source Data for Figure 4 [file EMBJ-39-e102817-s005.pdf]
