# Supplementary material for: Respiratory supercomplexes act as a platform for complex III‐mediated maturation of human mitochondrial complexes I and IV
Source: EMBO J. 2020 Jan 8;39(3):e102817. doi: 10.15252/embj.2019102817 (PMC6996572; doi:10.15252/embj.2019102817)

Figure 6B

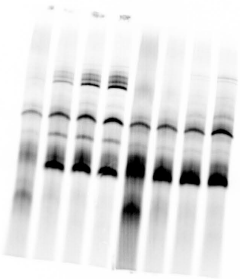

Figure 6A

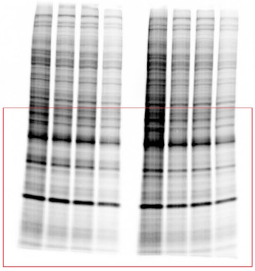

Figure 6C

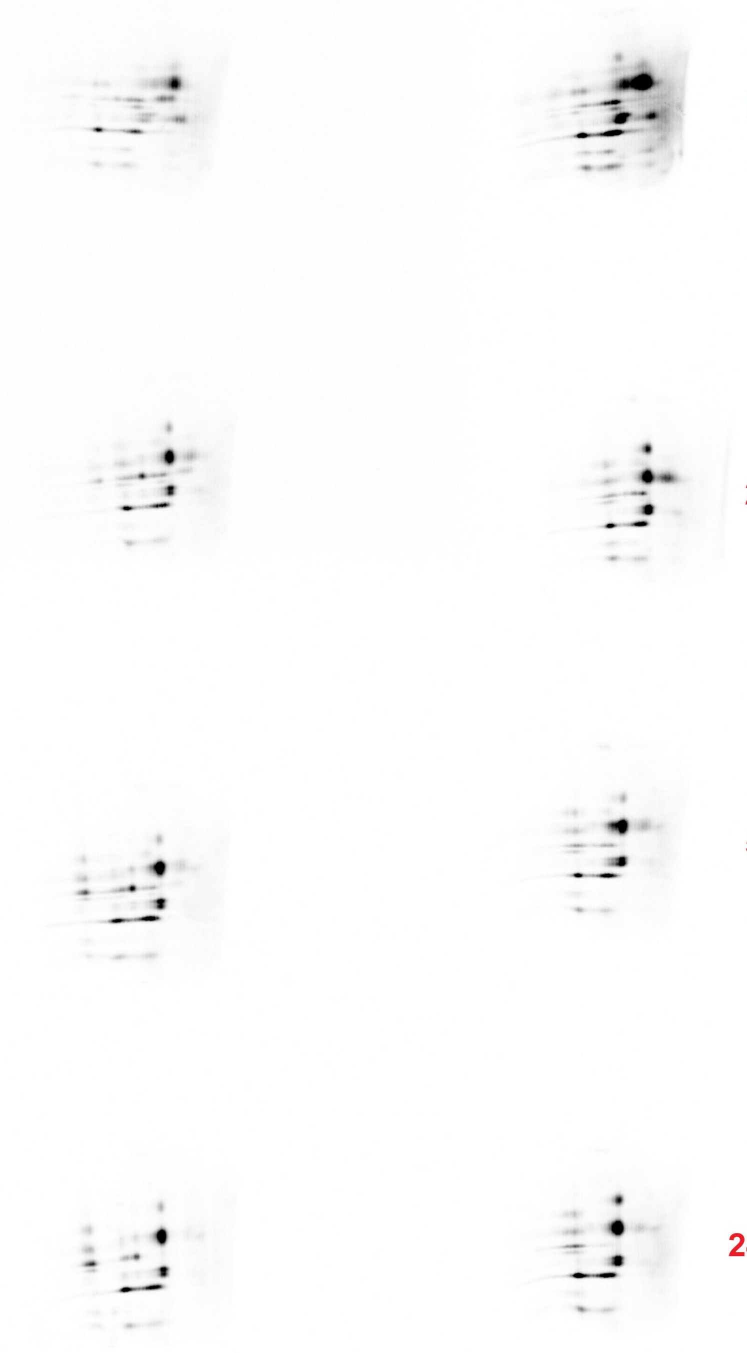

Figure 6D

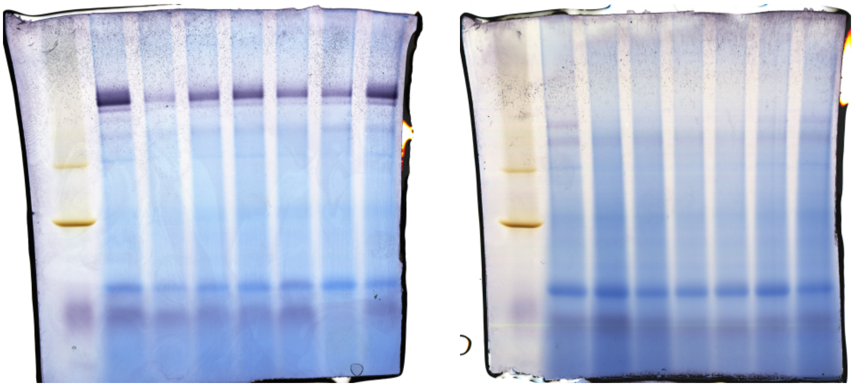

Figure 6E_1 – WT Low exposure

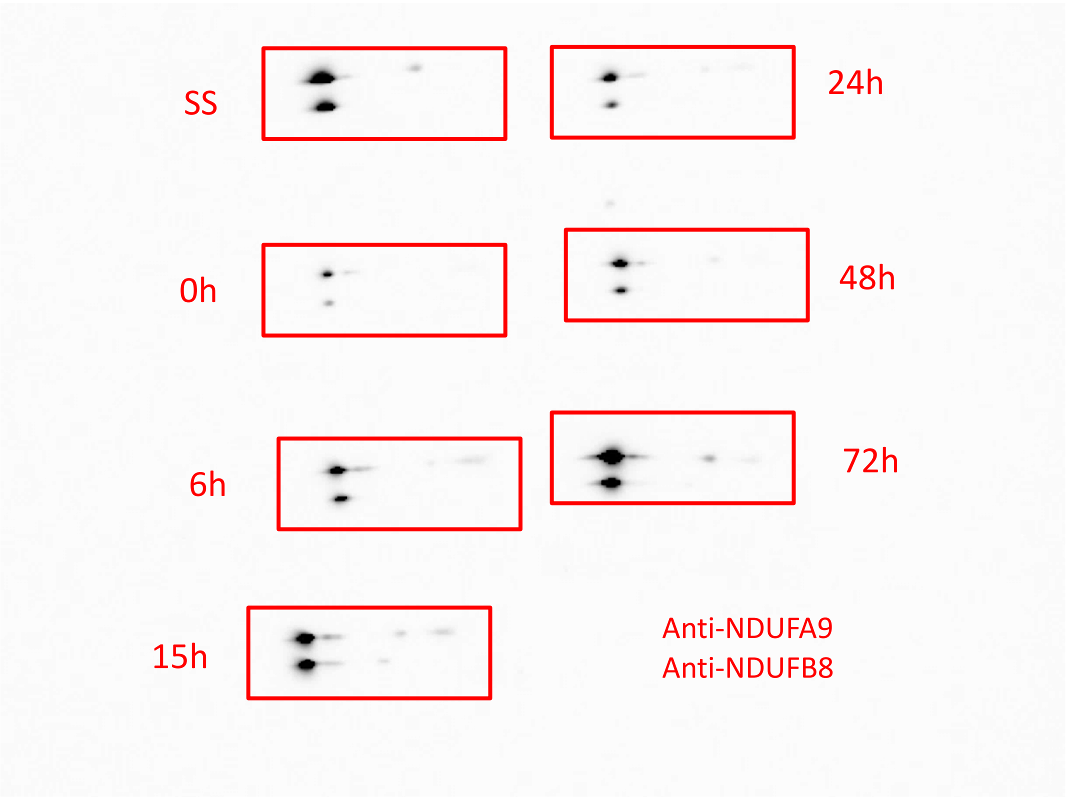

Figure 6E_2 -- Δ4-CYB Low exposure

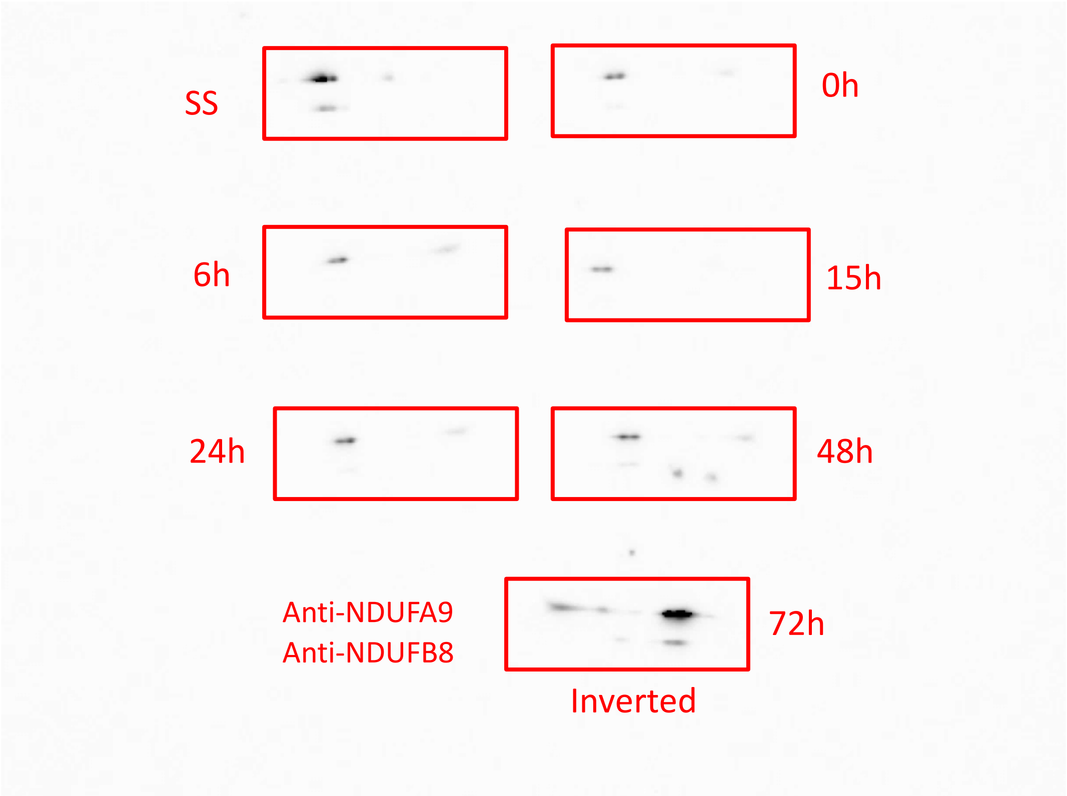

Figure 6E_3 -- Δ4-CYB High exposure

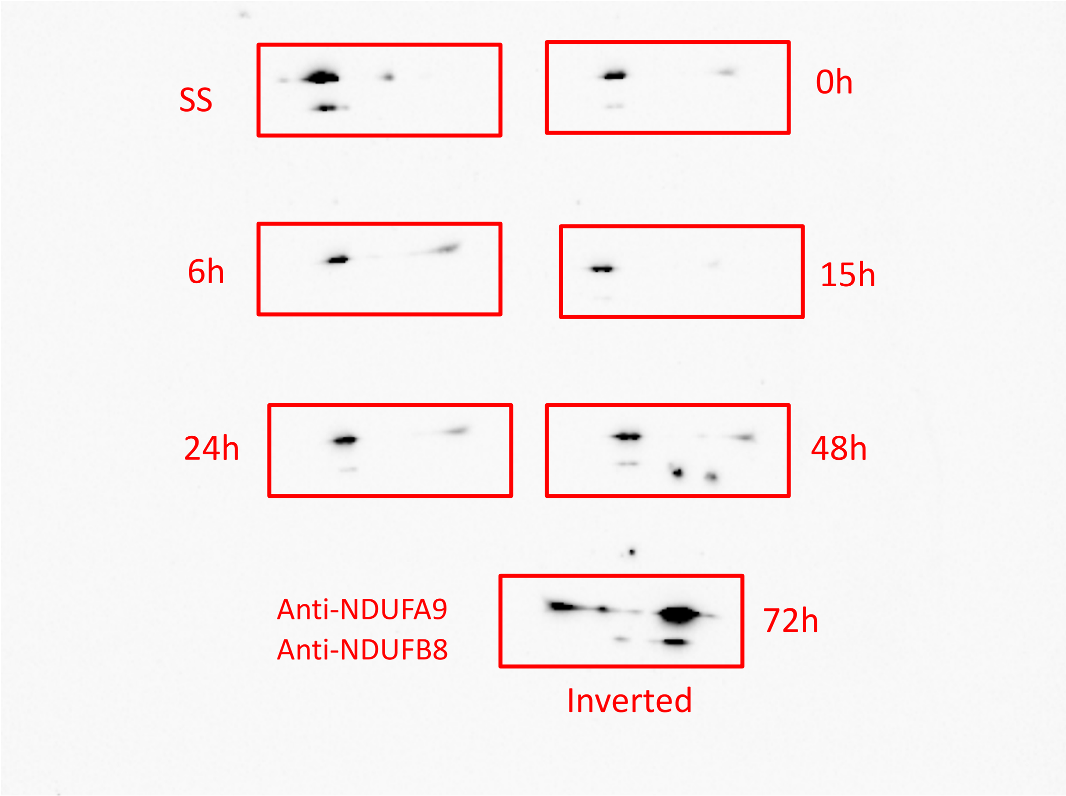

Figure 6E_4—WT Low exposure

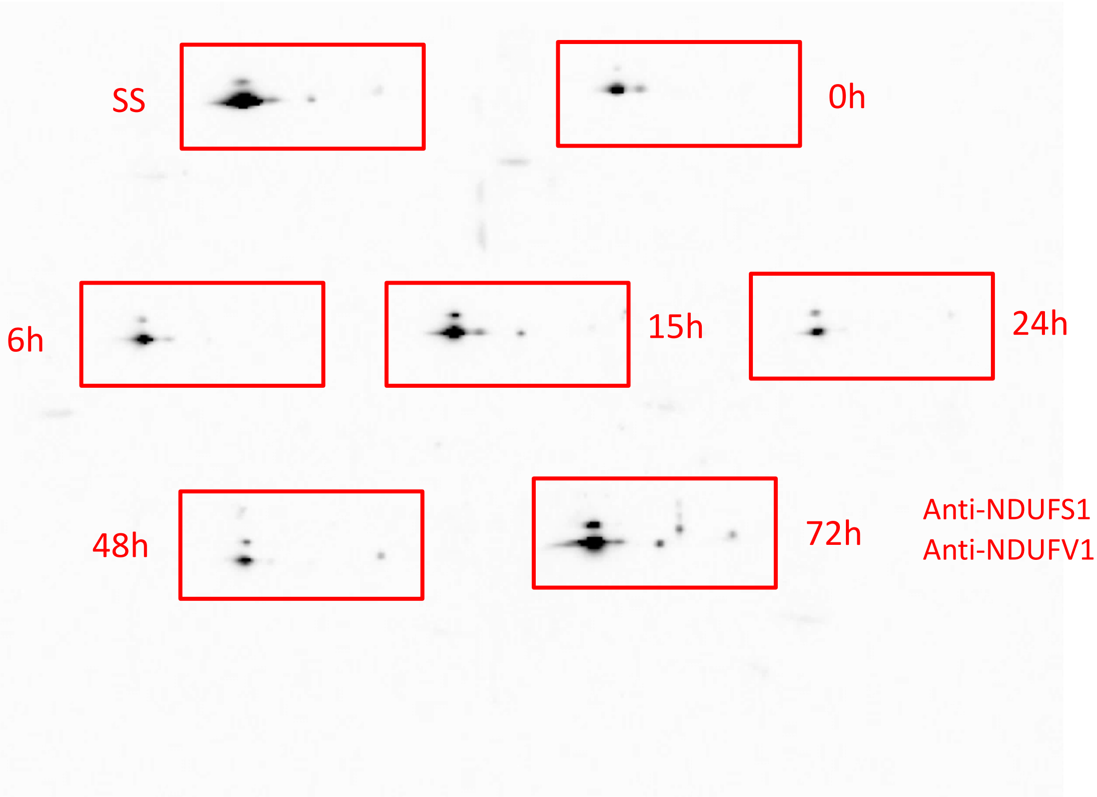

Figure 6E_5-- Δ4-CYB Low exposure

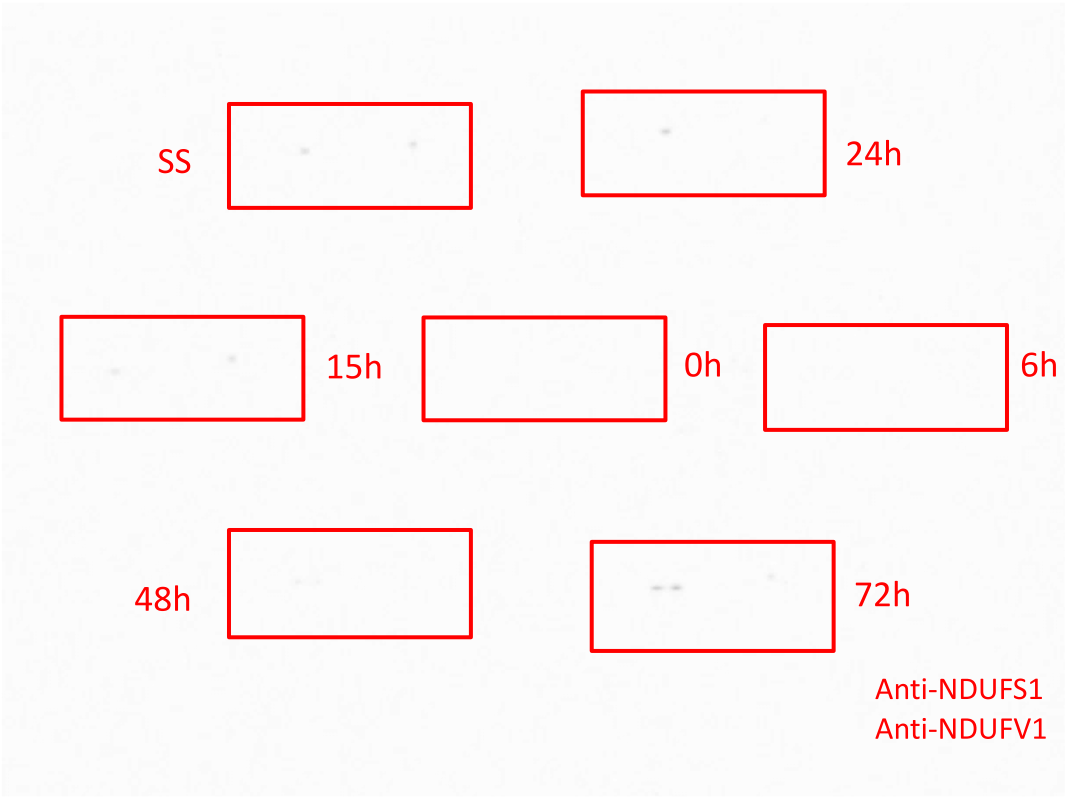

Figure 6E_5+Merge with membranes

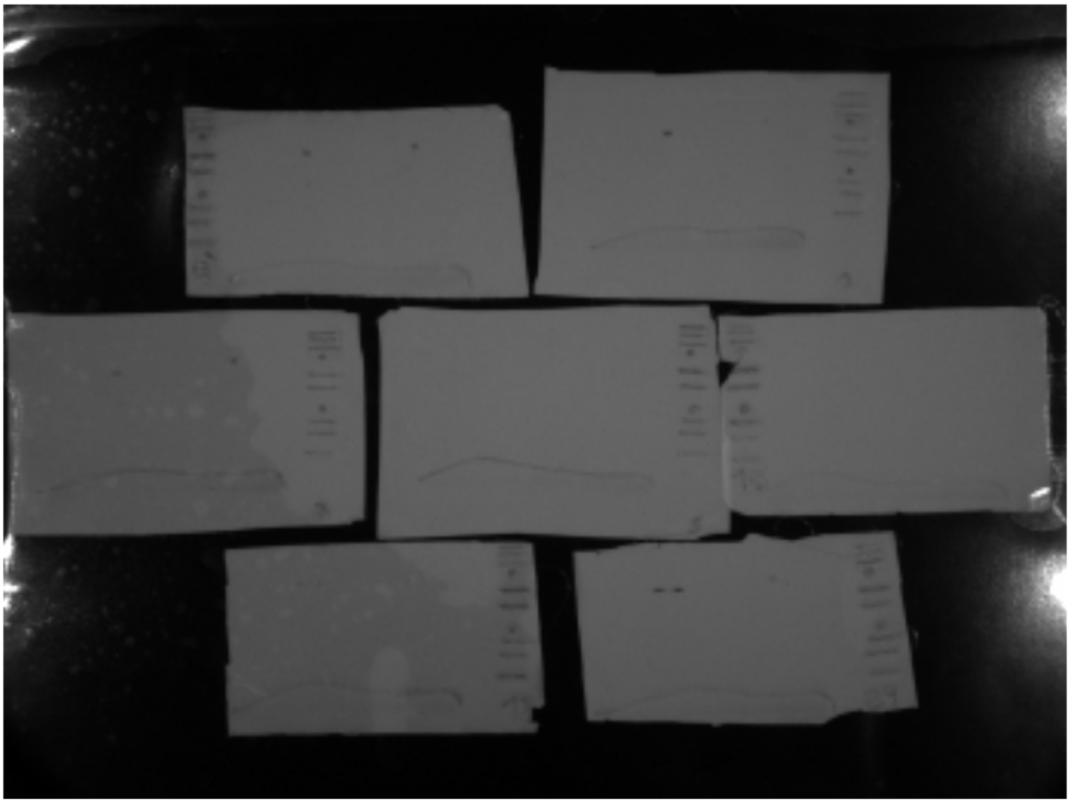

Figure 6E_6

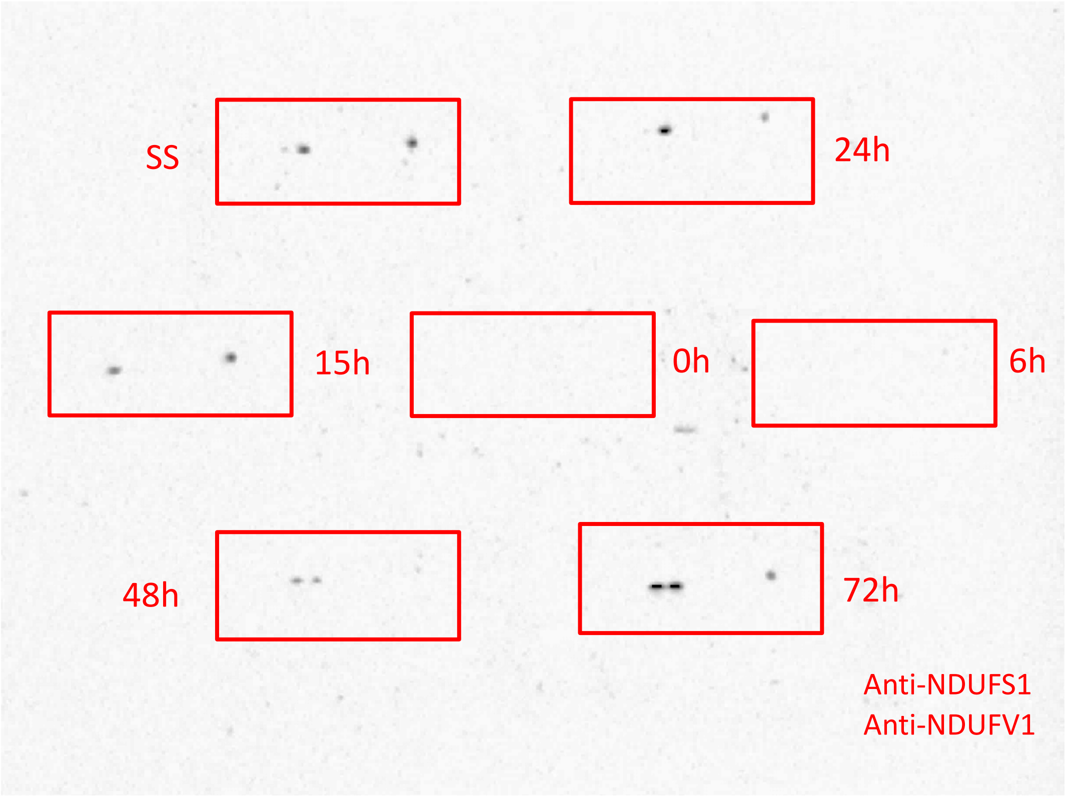

Figure 6E_6+Merge with membranes

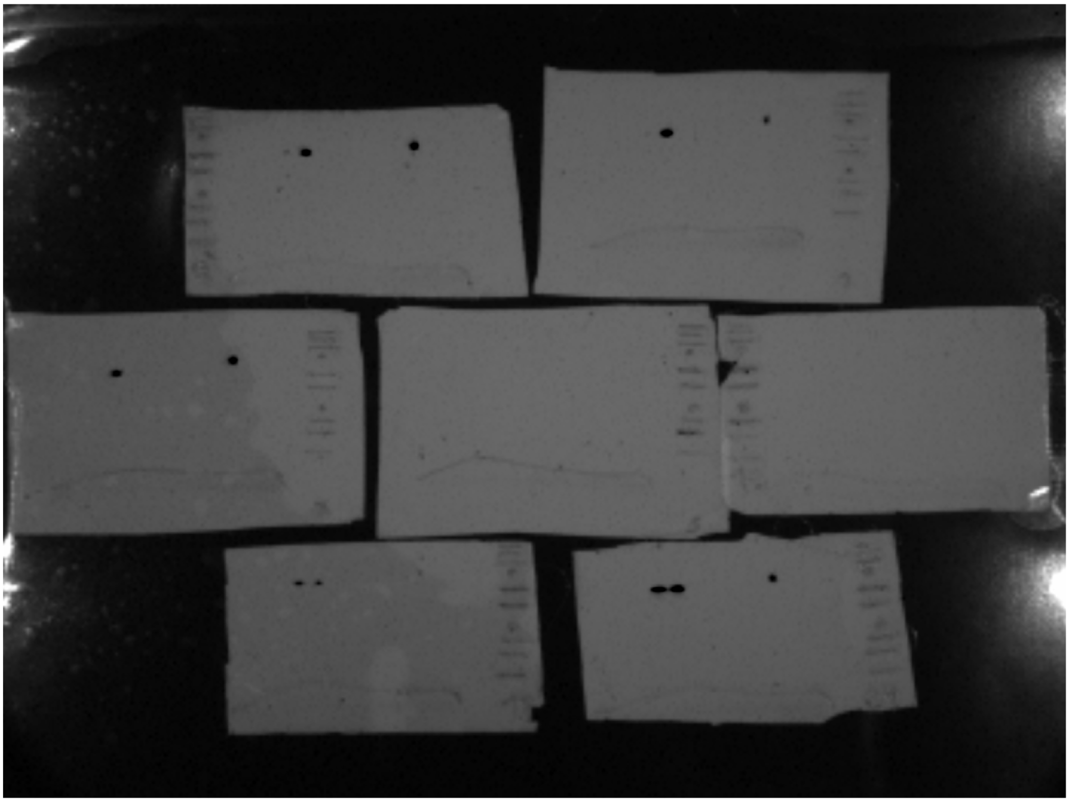

Supplement: Supplementary file 8 — Source Data for Figure 6 [file EMBJ-39-e102817-s006.pdf]
