# Supplementary material for: Respiratory supercomplexes act as a platform for complex III‐mediated maturation of human mitochondrial complexes I and IV
Source: EMBO J. 2020 Jan 8;39(3):e102817. doi: 10.15252/embj.2019102817 (PMC6996572; doi:10.15252/embj.2019102817)

# Figure 7A – Anti-β-Tubulin and Anti-HA

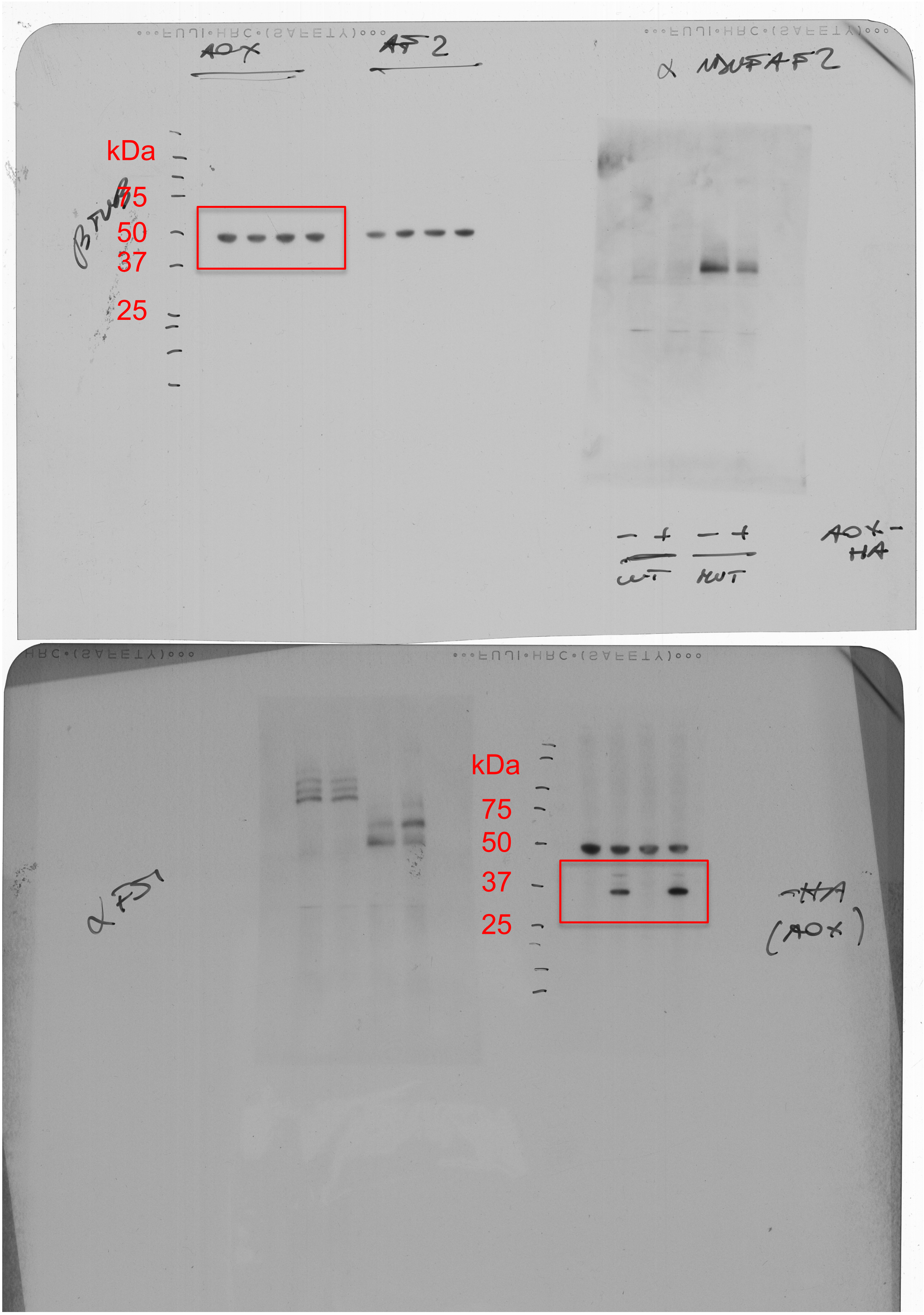

# Figure 7C – Anti-NDUFAF2

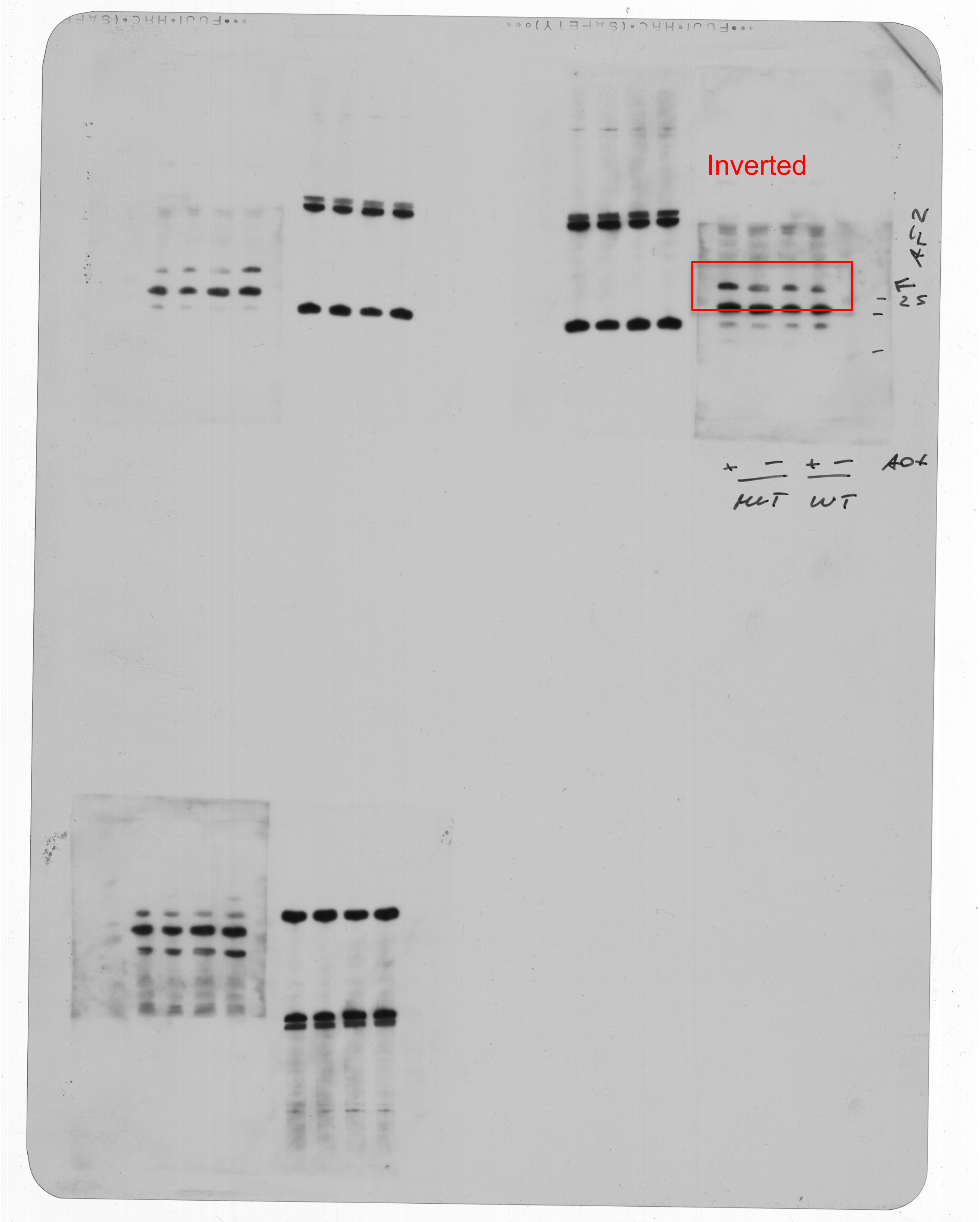

# Figure 7C – Anti-β-Tubulin and Anti-NDUFB8

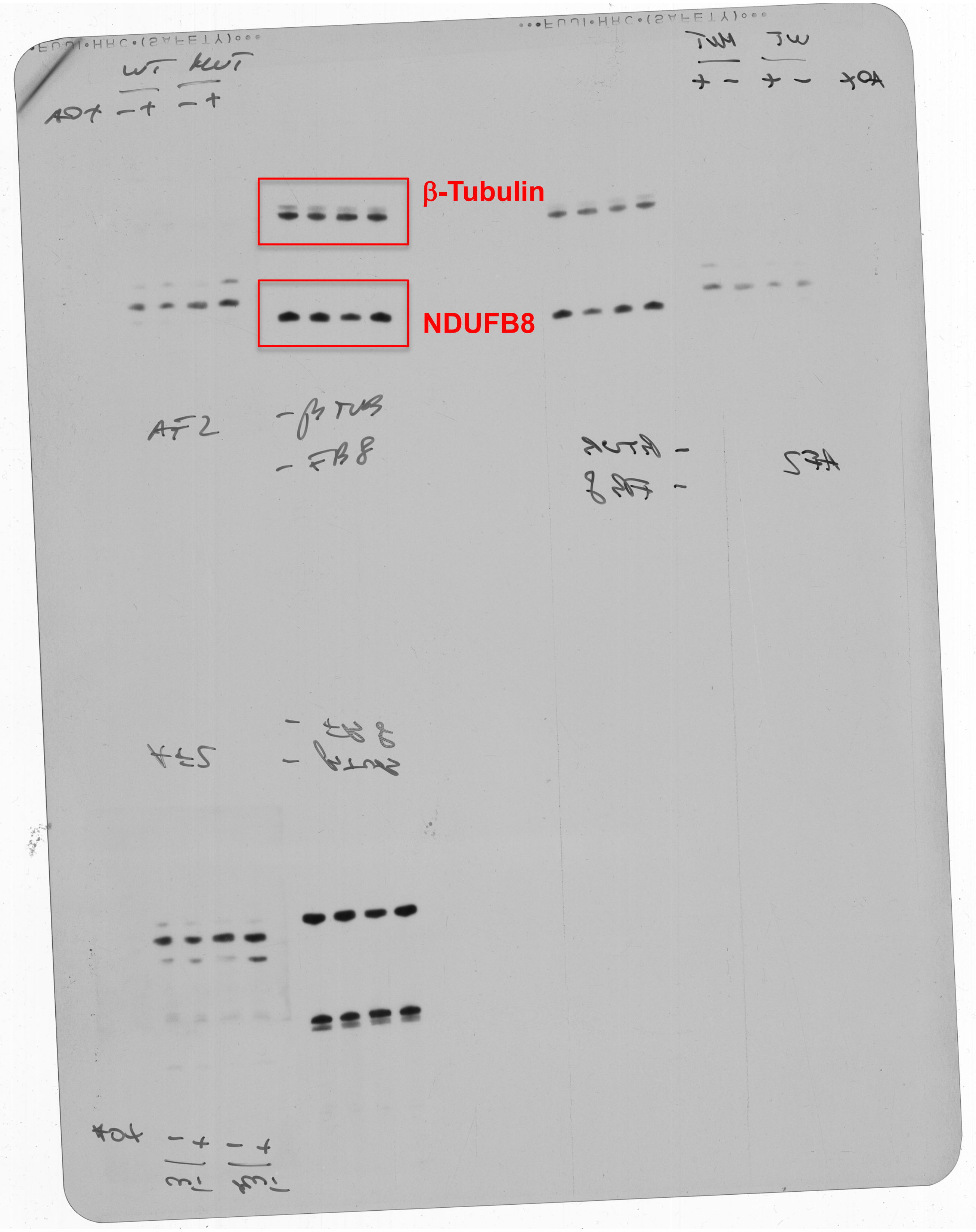

Figure 7C – Anti-NDUFS1

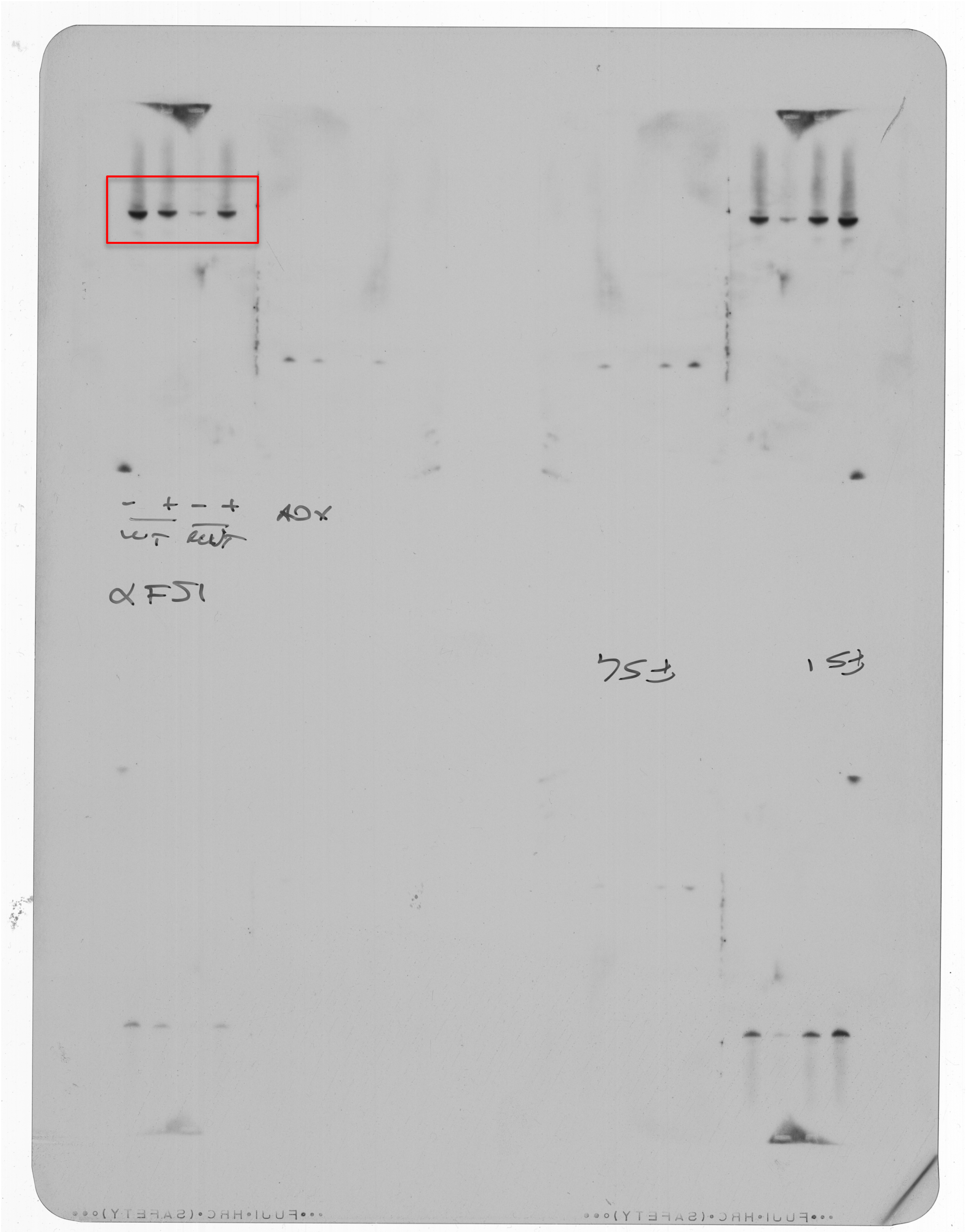

Figure 7D – Anti-NDUFAF2 & Anti-NDUFS1

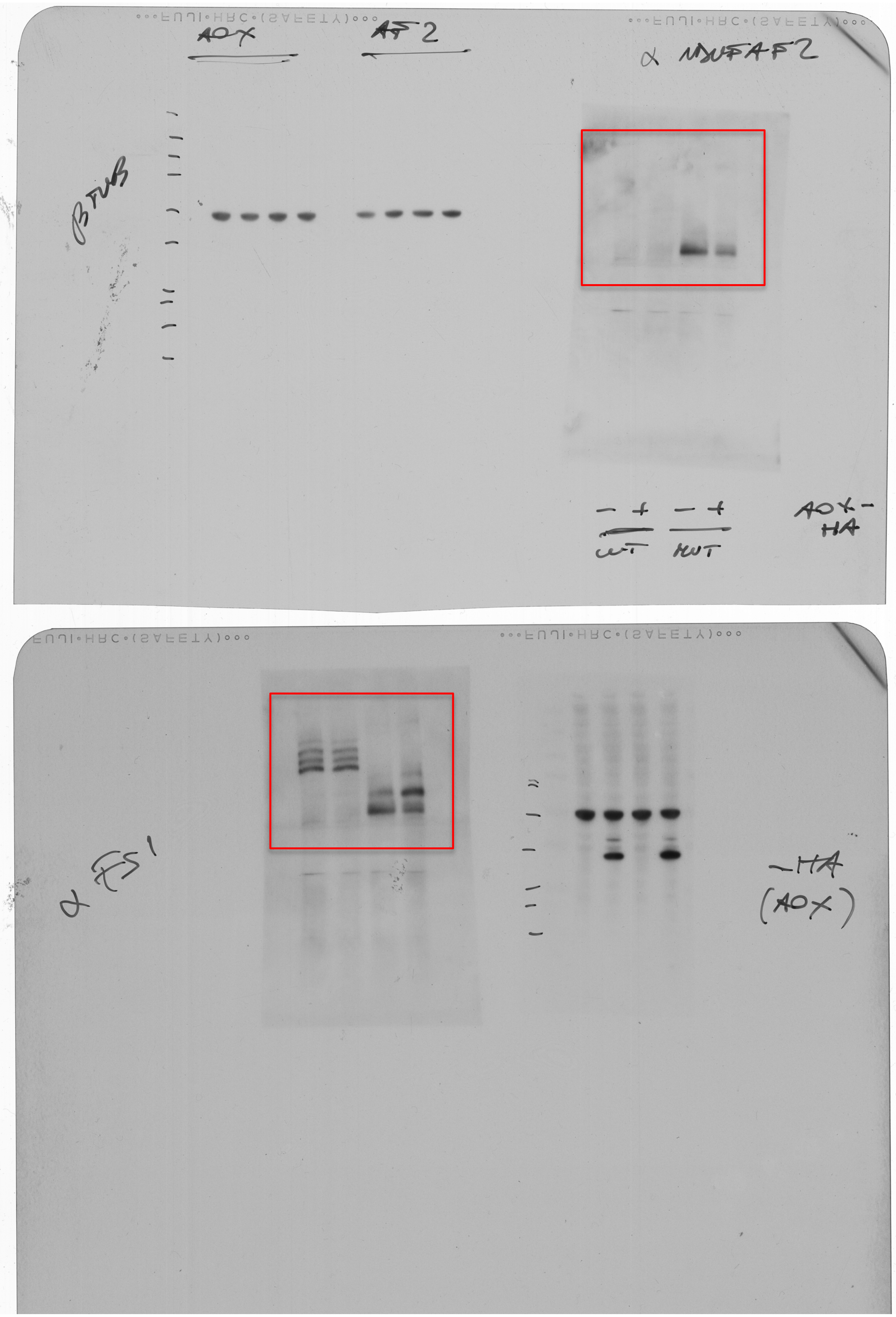

# Figure 7D – Anti-SDHB & Anti-HA

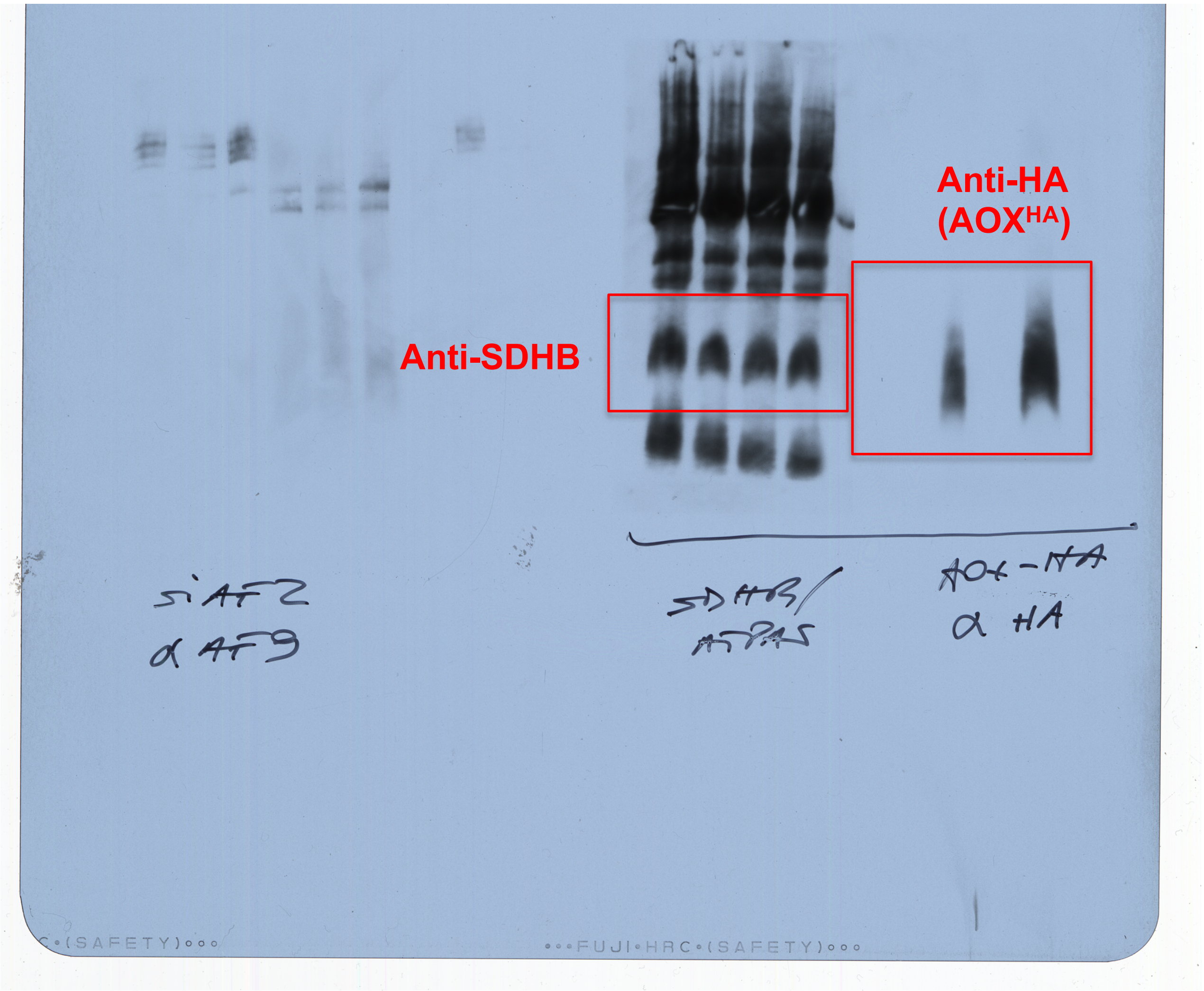

Figure 7E

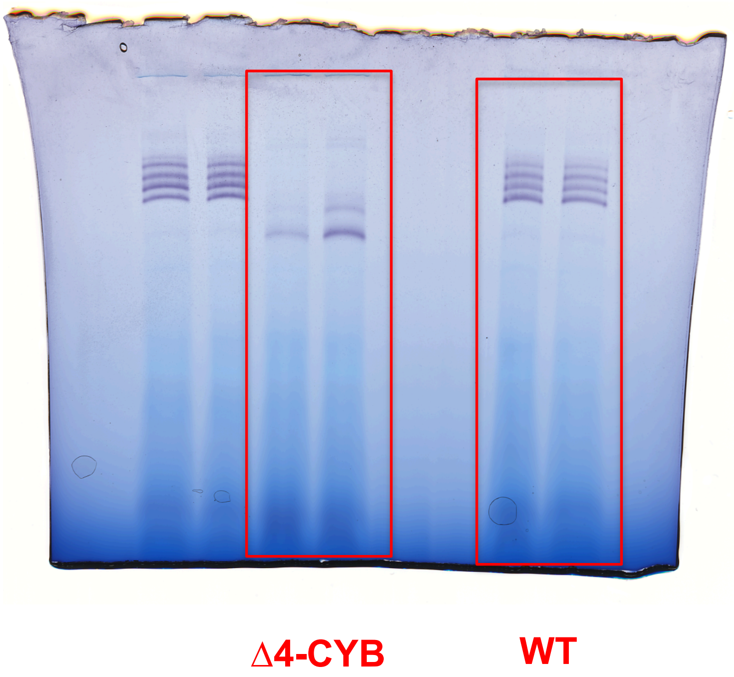


Figure 7I -- CI-In Gel Activity

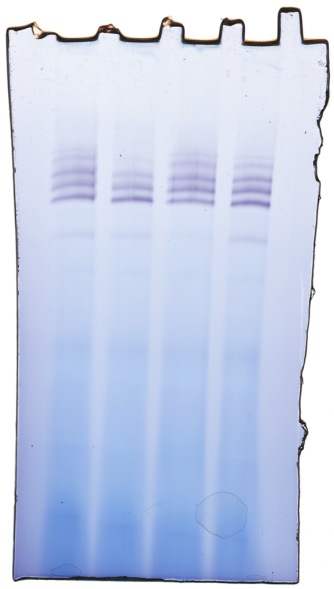

Figure 7I -- Anti-CYC1

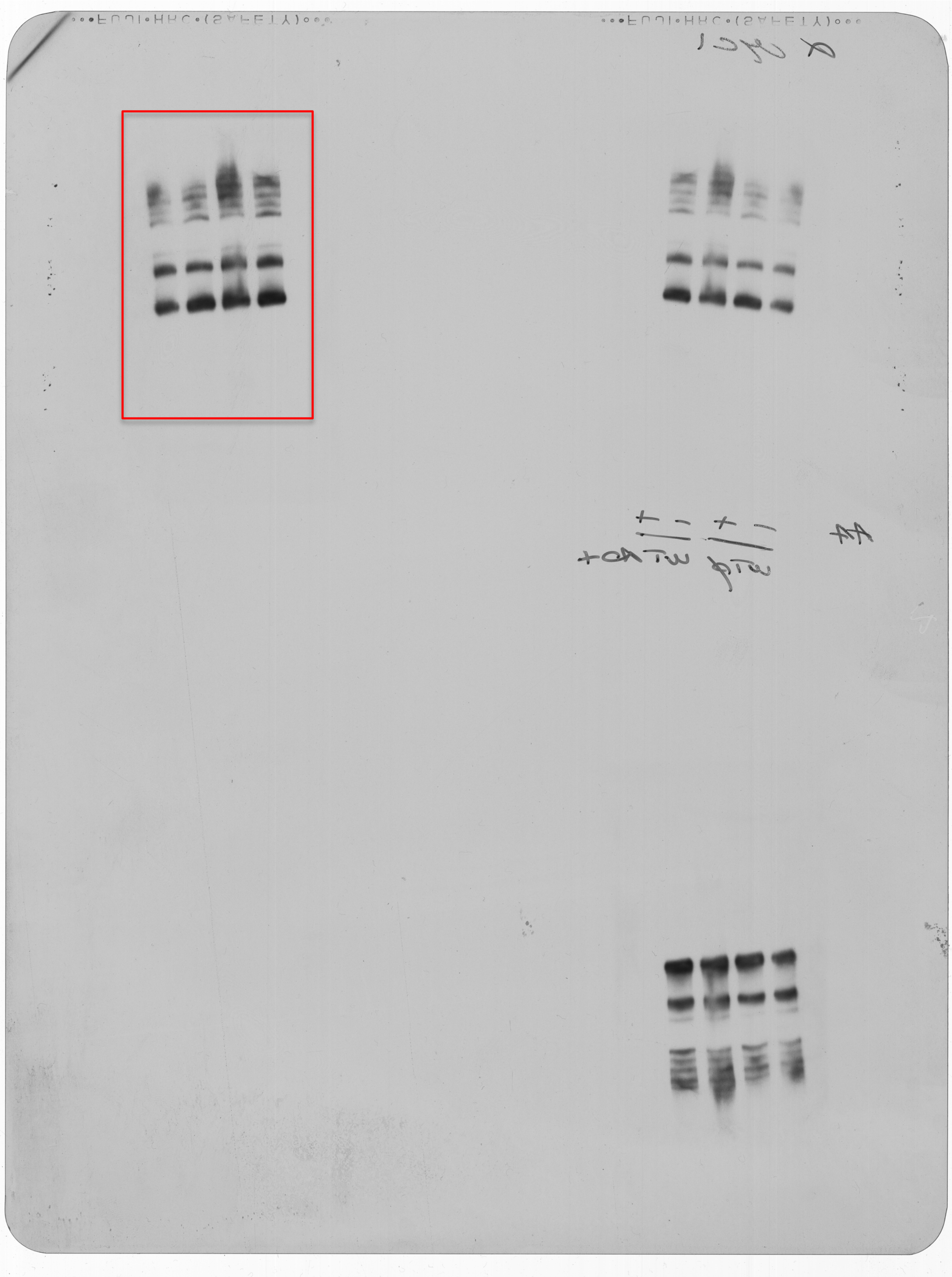

Supplement: Supplementary file 9 — Source Data for Figure 7 [file EMBJ-39-e102817-s007.pdf]
